# Exploring the relationship between pain and markers of disease activity in juvenile idiopathic arthritis: a scoping review

**DOI:** 10.1093/rheumatology/keag544

**Published:** 2026-09-29

**Authors:** Marelle K Wilson, Tasha R Stanton, Amy Rees, Hannah G Jones, Micah D J Peters, Grainne M Murray, Rachel S Tattersall, Rebecca R Lee, Stephen Colagiuri, Sarah B Wallwork

**Affiliations:** Innovation, IMPlementation And Clinical Translation (IIMPACT) in Health, Adelaide University, Adelaide, Australia; Innovation, IMPlementation And Clinical Translation (IIMPACT) in Health, Adelaide University, Adelaide, Australia; Persistent Pain Research Group, Hopwood Centre for Neurobiology, Lifelong Health Theme, South Australian Health and Medical Research Institute (SAHMRI), Adelaide, Australia; Innovation, IMPlementation And Clinical Translation (IIMPACT) in Health, Adelaide University, Adelaide, Australia; Persistent Pain Research Group, Hopwood Centre for Neurobiology, Lifelong Health Theme, South Australian Health and Medical Research Institute (SAHMRI), Adelaide, Australia; Innovation, IMPlementation And Clinical Translation (IIMPACT) in Health, Adelaide University, Adelaide, Australia; Persistent Pain Research Group, Hopwood Centre for Neurobiology, Lifelong Health Theme, South Australian Health and Medical Research Institute (SAHMRI), Adelaide, Australia; Rosemary Bryant AO Research Centre, College of Health, Adelaide University, Adelaide, Australia; National Policy Research Unit, Australian Nursing and Midwifery Federation (ANMF) Federal Office, Melbourne, Victoria, Australia; Health Evidence Synthesis, Recommendations and Impact (HESRI), School of Public Health, College of Health, Adelaide University, Adelaide, South Australia, Australia; The Danish Centre of Systematic Reviews: A Joanna Briggs Institute Centre of Excellence, Department of Clinical Medicine, Aalborg University, Aalborg, Denmark; Office of the Pro-Vice Chancellor (First Nations Strategy), University of Southern Queensland, Ipswich, Queensland, Australia; School of Nursing and Midwifery, Edith Cowan University, Joondalup, Western Australia, Australia; Rheumatology Department, Women’s and Children’s Health Network, Adelaide, Australia; Paediatric & Adolescent Rheumatology, Sheffield Teaching Hospitals and Sheffield Children’s Hospitals NHS Foundation Trusts, Sheffield, UK; The Centre for Epidemiology, The Centre for Musculoskeletal Research, Division of Musculoskeletal and Dermatological Sciences, Faculty of Biology, Medicine and Health, University of Manchester, Manchester, UK; Manchester Centre for Health Psychology, Division of Psychology and Mental Health, University of Manchester, Manchester, UK; Faculty of Medicine and Health, University of Sydney, Sydney, Australia; Juvenile Arthritis Foundation Australia, Sydney, Australia; Innovation, IMPlementation And Clinical Translation (IIMPACT) in Health, Adelaide University, Adelaide, Australia; Robinson Research Institute, Adelaide University, Adelaide, Australia

**Keywords:** juvenile idiopathic arthritis, pain, disease activity, clinical markers, imaging, biomarkers, discordance, scoping review

## Abstract

**Objectives:**

Pain is common in JIA and may be challenging to treat. Clinical observation has highlighted that pain and markers of disease activity may not align, with important implications for clinical management. This scoping review aimed to map and describe the extent, nature and findings of studies examining relationships between pain and objective or clinical disease activity markers in children and young people with JIA.

**Methods:**

Joanna Briggs Institute methodology for scoping reviews was followed and the review reported against the Preferred Reporting Items for Systematic Reviews and Meta-Analyses extension for Scoping Reviews checklist reporting guideline. MEDLINE, EMBASE and Scopus were searched from inception to 12 June 2025. Studies examining the relationship between self- or proxy-reported pain and at least one objective or clinical disease activity marker in individuals with JIA aged ≤24 years were included. Data were charted capturing study characteristics, pain measures, disease activity markers, analytic methods and reported relationships. Findings were synthesized descriptively and presented in tabular, visual and narrative formats.

**Results:**

One hundred and twenty-two studies were included. Overall concordance between pain and disease activity markers was uncommon. Pain aligned more consistently with physician-assessed clinical measures and composite DAS than with blood biomarkers or imaging, although the clinical measures and composite DAS may have included subjective or pain-related components.

**Conclusion:**

Relationships between pain and markers of disease activity in JIA are complex and frequently discordant. Divergence between pain and disease activity markers should be anticipated in clinical care and interpreted in parallel rather than as interchangeable indicators.

*Rheumatology* key messages

- Pain and disease activity markers frequently diverge in children and young people with JIA.
- Uncertainty about what pain means in relation to disease activity may be a source of anxiety for children and young people with JIA.
- Pain and disease activity markers should be interpreted in parallel rather than as interchangeable indicators.

## Introduction

Pain is the most common and distressing symptom reported by children and young people (CYP) with JIA [1, 2]. In a national survey of Australian CYP with JIA, 50% reported experiencing moderate to severe pain in the past week, with only 15% reporting no pain [1]. Earlier studies also report a substantial burden of pain, with around one-third of children experiencing moderate to high pain [3].

Pain can persist despite aggressive disease-focused treatments and when standard clinical or laboratory findings suggest low disease activity [4–7]. For CYP and their carers, pain in the absence of disease activity can lead to uncertainty about its cause and may result in pain being disbelieved, dismissed or minimized [8, 9], contributing to distress and self-doubt, and undermining trust in healthcare providers [10, 11]. Pain assessment in paediatric rheumatology may also be limited by clinician related factors, including limited training, low confidence, reluctance to engage in pain discussions and low prioritization of pain relative to disease activity [8]. In contrast, the absence of pain despite indications of inflammatory disease activity has also been reported [7, 12–14], raising concerns about missed or delayed recognition of active disease.

Clarifying the relationship between pain and disease activity markers in JIA is needed to guide the interpretation of pain in relation to underlying disease activity, including the risk of missed active disease, and to inform pain-specific management when pain persists despite markers indicating low or inactive disease.

Therefore, this scoping review aimed to map and describe the extent and nature of studies that explored the relationship between pain and objective or clinical disease activity markers in children and young people with JIA. The review described approaches used to assess subjective pain, the types of objective or clinical disease activity markers examined, and whether consistent patterns of concordance or discordance between pain and disease activity markers were identified.

## Methods

This scoping review was conducted between May 2025 and December 2025 in accordance with the Joanna Briggs Institute (JBI) methodology for scoping reviews [15, 16] and reported following the Preferred Reporting Items for Systematic Reviews and Meta-Analyses extension for Scoping Reviews checklist (PRISMA-ScR) [17]. The completed PRISMA-ScR checklist is provided in Appendix 2. A protocol was registered on the Open Science Framework on 29 May 2025 (10.17605/OSF.IO/RD5AK). An amendment was made to exclude studies that used quantitative sensory testing or brain imaging as objective markers as they do not represent disease activity in JIA. The term ‘clinical’ was added alongside ‘objective’ to the review question(s), and the ‘concept’ component of the eligibility criteria, to better reflect composite disease activity scores (DAS) that include a subjective patient or parent global assessment of well-being in addition to objective findings.

### Eligibility criteria

#### Participants

CYP with any JIA subtype, aged up to 24 years of age, consistent with international definitions of adolescence and emerging adulthood [18].

#### Concept

Pain refers to the subjective, personal experience of pain, as reported by the child or caregiver via validated measure or narrative history [19]. Studies that used pain elicited during clinical examination (pain or tenderness on palpation or movement), or evoked or experimentally induced pain (quantitative sensory testing or algometry) as substitutes for self-reported pain were excluded (unless self-reported pain was also measured), as these represent distinct pain constructs [20, 21].

Objective or clinical disease activity markers were defined as physician-assessed clinical measures, laboratory, imaging or composite indicators used to approximate inflammatory disease activity in JIA. Physician-assessed clinical measures included physician global assessment (PhGA; the clinician’s overall judgement of disease activity) and joint-based counts, including active joint count (the number of joints with clinically active arthritis), as well as swollen, tender, limited range of motion or enthesitis counts [22]. Laboratory measures included inflammatory or other disease-related biomarkers. Imaging measures included US, MRI and other radiographic modalities. Composite measures included validated DAS that integrate multiple clinical components and subjective patient or parent reported elements [e.g. Juvenile Arthritis Disease Activity Score (JADAS)] [23], as well as multi-criterion disease state definitions based on clinical and laboratory criteria alone (e.g. Wallace criteria) [24]. Collectively, these measures serve as proxies for underlying inflammatory pathology, as no single measure provides a definitive gold standard for disease activity in JIA, which is inferred clinically rather than directly measured.

Studies that investigated the relationship between pain and at least one objective or clinical marker of disease activity were included. Studies that examined pain only in relation to psychosocial or contextual variables were excluded.

#### Context

Studies were not restricted based on treatment status, setting or geographic location; however, searches were limited to studies published in English due to practical constraints related to translation resources.

### Types of sources

Quantitative studies of any design were included. Reviews were excluded, but reference lists were screened to identify potentially eligible primary studies. Grey literature (e.g. theses, conference abstracts) were included if sufficient data were available to assess eligibility and extract findings.

#### Search

The search strategy was developed in consultation with an academic librarian. An initial limited search of Google Scholar and MEDLINE (Ovid) was undertaken to identify a range of relevant studies. Words contained in study titles and abstracts were analysed to develop a comprehensive search strategy. The search was undertaken in MEDLINE (Ovid), EMBASE (Ovid) and Scopus on 12 June 2025 (Appendix 1). Reference lists of included studies and relevant reviews were screened for additional eligible studies. No date limits were applied to maximize coverage of the literature. A search of Google Scholar (first two pages of results) using relevant keywords was undertaken on 12 June 2025 from Adelaide, Australia to supplement formal database searches.

#### Study selection

Search results were uploaded into Endnote™ reference manager (Version 20.6, 2023, Clarivate Analytics, Philadelphia, PA, USA) and then exported to Covidence™ (Veritas Health Innovation Ltd, Melbourne, Australia), where duplicates were removed.

Following piloting, titles and abstracts were screened by two independent reviewers (M.K.W. and A.R.) for assessment against the inclusion criteria before full text versions were assessed by two independent reviewers (M.K.W. and A.R.). Reasons for exclusion were recorded (Appendix 3). Any disagreements between reviewers at each stage of the selection process were resolved through discussion, or with an additional reviewer.

### Data extraction

A draft data extraction tool (Appendix 4) was developed within Covidence™ (Veritas Health Innovation Ltd, Melbourne, Australia). To reduce variation in reporting of the relationship between pain and disease activity and enable consistent classification, we collapsed relationship findings into three categories: ‘concordant’, ‘discordant’ or ‘mixed’. Concordant included statistically significant positive correlations reported by study authors, predictive relationships, or narrative descriptions in which pain increased or decreased in line with disease activity as would be hypothesized, where inflammatory activity and pain move in the same direction. Discordant included directional discordance (pain greater or lower than markers of disease activity) and weak or non-significant statistical associations reported by study authors. Mixed included cases where both concordant and discordant findings were reported, either within a single pain-disease activity marker analysis or across multiple relationships within a study. The tool was piloted and refined by two reviewers during extraction from an initial selection of sources. Data were extracted from studies included in the scoping review by one reviewer (M.K.W.). Extracted data from all studies were verified by a second reviewer (H.G.J.). NotebookLM (Google, 2024) was used to cross-check extracted data from 50 of the 122 studies (41%), selected alphabetically by first author. The number was limited by the platform’s maximum of 50 source documents at the time. Any disagreements were resolved through discussion between the two reviewers.

### Critical appraisal of individual sources of evidence

Consistent with JBI guidance for scoping reviews, no formal critical appraisal of individual studies was undertaken due to the diversity of study designs and levels of evidence [15, 16].

### Data synthesis

Extracted data were synthesized in accordance with JBI guidance for scoping reviews [15, 25]. Study design, aims, participant characteristics, pain measures, and objective or clinical markers were summarized directly from extraction tables. Analysis of the relationship between pain and objective or clinical markers was iterative, beginning with synthesis of concordance findings at the study level to identify early patterns. For marker-specific syntheses, individual pain-marker analyses within studies were treated as the unit of analysis. For the purposes of synthesis, disease activity markers were grouped at the level of marker domain. Multiple measures within the same domain (e.g. different imaging approaches or multiple blood biomarkers) were treated as a single disease activity construct, whereas measures from distinct domains (e.g. active joint count and PhGA) were treated as separate constructs when analysed independently. Composite DAS (e.g. JADAS) and multi-criterion disease state definitions (e.g. Wallace criteria) were treated as a single construct when pain was analysed only in relation to the overall score or disease state. Exploratory subgroup analyses were undertaken using coded study characteristics such as pain measurement, participant age, sex distribution, JIA subtype, disease status or duration, and anatomical region. Patterns were summarized narratively and visually using a proportional bar chart by disease activity marker category. Individual study findings were detailed in the supplementary tables.

## Results

Electronic searches retrieved 18 231 citations, with 18 additional records identified from manual citation searching. Following full-text screening, data were extracted from 122 studies representing 57 245 participants. Figure 1 presents the PRISMA flowchart of the screening process.

**Figure 1:**
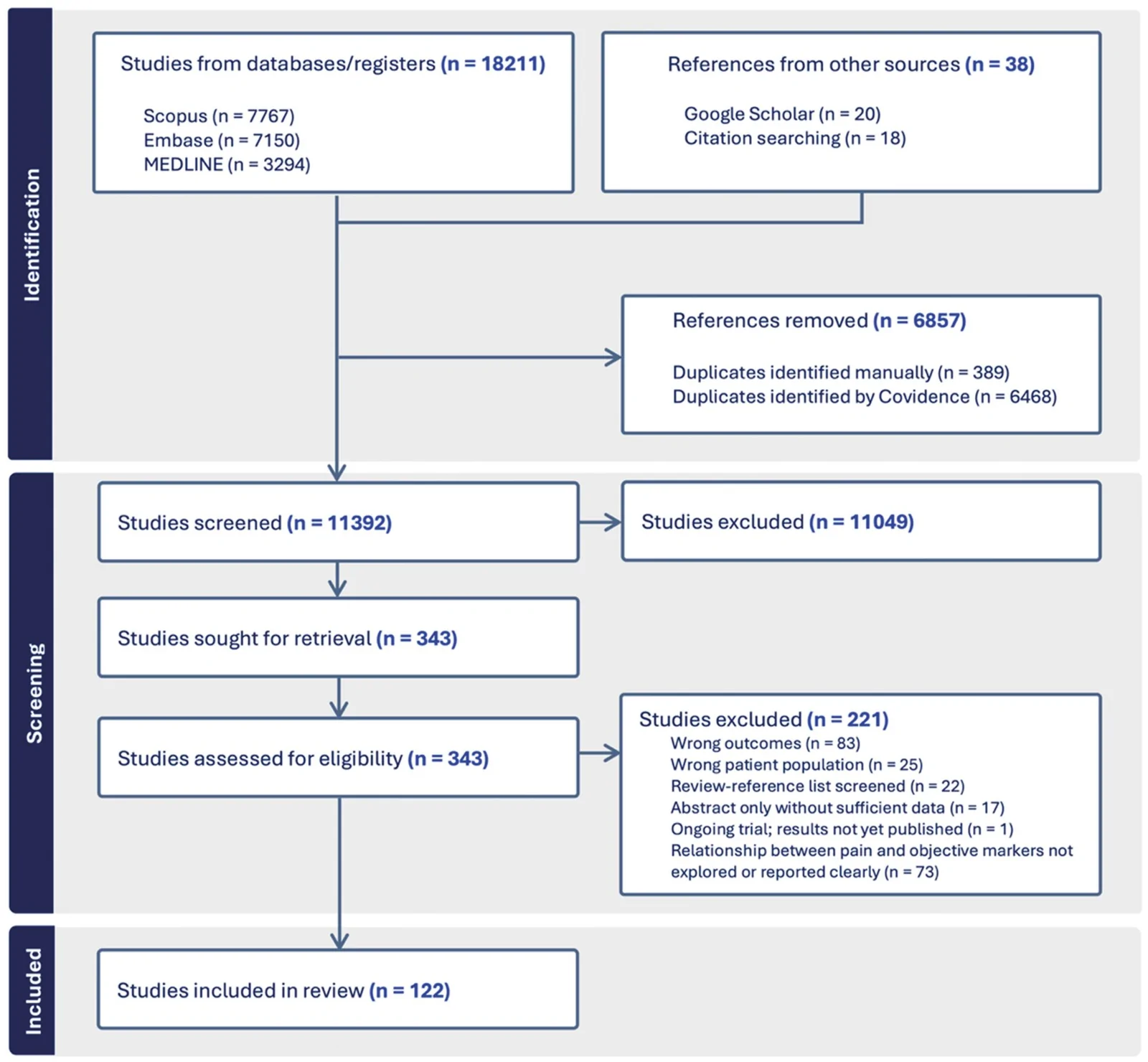
Preferred Reporting Items for Systematic Reviews and Meta-Analyses (PRISMA) flowchart illustrating the screening process

### Study and participant characteristics

Included studies were conducted in 27 countries, although concentrated in Europe (including the UK) and North America. The literature was dominated by observational designs, mainly cross-sectional and longitudinal studies. Sample sizes were typically between 10 and 100 participants, although several large registry and inception cohort studies contributed much larger samples, with some exceeding 5000 participants. Overall study characteristics are summarized in Table 1.

**Table 1:** Study characteristics.

First author/year	Title	Source type	Country	Study design	Sample size
Abu-Saad 1995 [37]	Pain in children with juvenile rheumatoid arthritis: a descriptive study	Journal article	Netherlands	Cross-sectional, descriptive	33
Algergawy 2011 [38]	Clinical, laboratory, and ultrasound assessment of the knee in juvenile rheumatoid arthritis	Journal article	Egypt	Cross-sectional	20, 20 controls
Alongi 2022 [28]	Drivers of non-zero physician global scores during periods of inactive disease in juvenile idiopathic arthritis	Journal article	Italy	Cross-sectional (pooled data from 3 datasets, including EPOCA)	9966
Alqanatish 2011 [39]	Infrapatellar bursitis in children with juvenile idiopathic arthritis: a case series	Journal article	Canada	Case series	5
Armbrust 2013 [40]	Assessment of disease activity by patients with juvenile idiopathic arthritis and the parents compared with the assessment by pediatric rheumatologists	Journal article	Netherlands	Cross-sectional (Nordic JIA cohort)	113
Arnstad 2019 [41]	Early self-reported pain in juvenile idiopathic arthritis as related to long-term outcomes: results from the Nordic Juvenile Idiopathic Arthritis Cohort Study	Journal article	Norway; Sweden; Finland; Denmark	Prospective cohort (Nordic JIA cohort)	243
Arnstad 2020 [21]	Pain sensitivity in young adults with juvenile idiopathic arthritis: a quantitative sensory testing study	Journal article	Norway	Prospective cohort (Nordic JIA cohort)	96
Bandeira 2006 [42]	Weighting improves the information provided by joint counts on the severity of arthritis and its impact on patients’ well-being in juvenile idiopathic arthritis	Journal article	Italy	Cross-sectional (two cohorts from Italian national database)	121
Biancardi 2015 [43]	Which is the clinical relevance of ultrasound examination in the assessment of established juvenile idiopathic arthritis?	Conference abstract	Not reported	Cross-sectional	28
Bloom 1995 [44]	Bicipital synovial cysts in juvenile rheumatoid arthritis: clinical description and sonographic correlation	Journal article	USA	Case series	6
Bou Antoun 2017 [45]	Clinical predictors of magnetic resonance imaging-detected sacroiliitis in children with enthesitis related arthritis	Journal article	France	Retrospective longitudinal cohort	20, 25 controls
Brandon 2017 [46]	Patient-reported outcomes measurement information system tools for collecting patient-reported outcomes in children with juvenile arthritis	Journal article	USA	Cross-sectional	265
Brandon 2021 [47]	Changes over time in inflammatory and structural lesions at the sacroiliac joint in children with spondyloarthritis exposed and unexposed to tumor necrosis factor inhibitor	Journal article	USA and Canada	Retrospective longitudinal cohort	46
Carlsson 2023 [48]	Juvenile idiopathic arthritis and the temporomandibular joint: a case–control study of magnetic resonance imaging findings in relation to clinical and psychosocial factors	Journal article	Sweden	Case–control	45, 16 controls
Chaplin 2018 [49]	Exploring a biopsychosocial approach to pain in adolescents and young adults with juvenile idiopathic arthritis (young investigator award winner 2018)	Conference abstract	UK	Cross-sectional	85
Collin 2024 [50]	Temporomandibular involvement in children and adolescents with juvenile idiopathic arthritis: a 2-year prospective cohort study	Journal article	Sweden	Prospective longitudinal cohort study	54
Coulton 1987 [51]	Assessment of the reliability and validity of the arthritis impact measurement scales for children with juvenile arthritis	Journal article	USA	Validation	77
de Oliveira Sato 2011 [52]	Progression of articular and extraarticular damage in oligoarticular juvenile idiopathic arthritis	Journal article	Brazil	Cross-sectional and retrospective	75
de Vos 2023 [53]	Does patient reported absence of swollen and painful joints really mean that the active joint count is zero?	Conference abstract	Netherlands	Retrospective	778
Dekker 2010 [54]	Strong relationships between disease activity, foot-related impairments, activity limitations and participation restrictions in children with juvenile idiopathic arthritis	Journal article	Netherlands	Cross-sectional	34
Eboli 2022 [55]	Understanding the role of ultrasound in detecting temporomandibular joint involvement in juvenile idiopathic arthritis: a pilot study	Conference abstract/poster	Italy	Cross-sectional pilot study	23
Elfving 2023 [56]	An explorative study on proteomic analyses related to inflammation and pain in children with juvenile idiopathic arthritis	Journal article	Sweden	Clinical explorative study; cross-sectional plus longitudinal	51, 18 controls
Elliott 1997 [57]	Suppression of fever and the acute-phase response in a patient with juvenile chronic arthritis treated with monoclonal antibody to tumour necrosis factor-alpha (cA2)	Journal article	UK	Case report	1
Esbersen 2018 [58]	Daily physical activity and sleep in children with juvenile idiopathic arthritis and healthy children in relation to pain	Conference abstract/poster	Denmark	Prospective observational	93, 188 controls
Ferrazzo 2019 [59]	Juvenile idiopathic arthritis with severe unilateral temporomandibular joint involvement	Conference abstract	Brazil	Case report	1
Flatø 2002 [60]	The influence of patient characteristics, disease variables, and HLA alleles on the development of radiographically evident sacroiliitis in juvenile idiopathic arthritis	Journal article	Norway	Longitudinal retrospective cohort	314
Gestler 2022 [61]	Residual complaints in juvenile idiopathic arthritis patients with inactive disease	Conference abstract/poster	Brazil	Retrospective	117
Gorczyca 2017 [62]	Serum levels of myeloid-related proteins 8 and 14 in oligoarticular and polyarticular onset of juvenile idiopathic arthritis	Conference abstract/poster	Poland	Cross-sectional	44, 25 controls
Guzman 2017 [63]	Predicting which children with juvenile idiopathic arthritis will have a severe disease course: results from the ReACCh-Out cohort	Journal article	Canada	Prospective multicentre inception cohort (ReACCh-Out)	609
Hara 2017 [64]	Usefulness of joint ultrasonography in the treatment of juvenile idiopathic arthritis	Conference abstract/poster	Japan	Case series	3
Hogewe 1995 [65]	The pain threshold in juvenile chronic arthritis	Journal article	Netherlands	Cross-sectional	57, 69 controls
Hospach 2014 [66]	Cervical spine involvement in patients with juvenile idiopathic arthritis—MRI follow-up study	Journal article	Germany	Retrospective	13
Hu 2019 [67]	The role of extracellular histones in systemic-onset juvenile idiopathic arthritis	Journal article	China	Longitudinal, laboratory-based	26, 30 controls
Ilowite 1992 [6]	Assessment of pain in patients with juvenile rheumatoid arthritis: relation between pain intensity and degree of joint inflammation	Journal article	USA	Cross-sectional	18
Katsicas 2017 [68]	Enthesitis-related arthritis: non-peripheral pattern is associated with an expansion of peripheral Th17 populations	Conference abstract/poster	Argentina	Cross-sectional	29
Katsicas 2018 [69]	Spondyloarthritis research consortium of Canada sacroiliac joint inflammation score in children with enthesitis-related arthritis	Conference abstract/poster	Argentina	Cross-sectional	30
Khanom 2022 [70]	Exploring the relationship between symptoms commonly associated with flares and disease activity	Thesis chapter	UK	Cross-sectional analysis of secondary data (CAPS)	97
Kovalchuk 2018 [71]	The difference in pain and overall well-being assessment between patients with juvenile idiopathic arthritis, their parents, and physicians in Ukraine	Journal article	Ukraine	Cross-sectional	60, 60 controls
Küseler 2005 [30]	A 2 year follow-up study of enhanced magnetic resonance imaging and clinical examination of the temporomandibular joint in children with juvenile idiopathic arthritis	Journal article	Denmark	Prospective longitudinal cohort	15
Learoyd 2019 [72]	The pain trajectory of juvenile idiopathic arthritis (JIA): translating from adolescent patient report to behavioural sensitivity in a juvenile animal model	Journal article	UK	Retrospective longitudinal, observational cohort	97
Leksell 2012 [73]	Orofacial pain and dysfunction in children with juvenile idiopathic arthritis: a case–control study	Journal article	Sweden	Cross-sectional, case–control	41, 41 controls
Lomholt 2013 [74]	Pain experience in children with juvenile idiopathic arthritis treated with anti-TNF agents compared with non-biologic standard treatment	Journal article	Denmark	Cross-sectional	91
Magni-Manzoni 2005 [75]	Responsiveness of clinical measures to flare of disease activity in juvenile idiopathic arthritis	Journal article	Italy	Retrospective	115
Magni-Manzoni 2009 [76]	Comparison of clinical *vs* ultrasound-determined synovitis in juvenile idiopathic arthritis	Journal article	Italy	Cross-sectional	32
Mahmood 2014 [77]	Cervical spine involvement in JIA: a clinical and radiological correlation	Conference abstract/poster	UK	Retrospective	43
Malattia 2010 [78]	Dynamic contrast-enhanced magnetic resonance imaging in the assessment of disease activity in patients with juvenile idiopathic arthritis	Journal article	Italy	Cross-sectional	22
Malleson 2004 [79]	Predictors of pain in children with established juvenile rheumatoid arthritis	Journal article	Canada	Secondary analysis of RCT or cohort study	388
Mannion 2019 [80]	Investigation of inactive disease activity states among JIA patients in the Carra registry	Conference abstract/poster	USA; Canada	Secondary analysis of cohort (CARRA Registry)	2164
Mannion 2022 [81]	Short term outcomes following tolerated disease activity level for individuals with juvenile idiopathic arthritis in the childhood arthritis and rheumatology research alliance registry	Conference abstract/poster	USA	Registry based (CARRA Registry)	6235
McGrath 2023 [26]	Predictors of persisting pain in children with juvenile idiopathic arthritis: a case control study nested in the ReACCh-Out cohort	Journal article	Canada	Case–control study nested in a prospective cohort (ReACCh-Out)	31 (persisting pain) 118 controls (decreasing pain)
McLellan 2023 [82]	Management of temporomandibular joint involvement (TMJ) in juvenile idiopathic arthritis (JIA) in our centre	Conference abstract/poster	UK	Retrospective	57
Melchiorre 2010 [83]	Sonographic evaluation of the temporomandibular joints in juvenile idiopathic arthritis	Journal article	Italy	Prospective cohort	68, 40 controls
Miller 2011 [84]	Physician assessment of disease activity in JIA subtypes. Analysis of data extracted from electronic medical records	Journal article	USA	Cross-sectional, retrospective	160
Min 2020 [85]	Establishment of a registry for juvenile idiopathic arthritis patients in South Australia: focus on patient-reported outcome and experience measures (PROMS/PREMS)	Conference abstract/poster	Australia	Registry establishment	112
Minden 2014 [86]	The health status of patients with juvenile idiopathic arthritis (JIA) significantly worsens after transfer from pediatric to adult care	Conference abstract/poster	Germany	Registry based, prospective cohort longitudinal	654
Miyamae 2019 [87]	Juvenile idiopathic arthritis with no complaints of joint pain in early childhood	Journal article	Japan	Case series report	3
Mohammed 2012 [88]	Juvenile idiopathic arthritis and the temporomandibular joint	Journal article	Egypt	Cross-sectional, case–control	40, 10 controls
Mosa 2021 [89]	Bicipital synovial cyst as the sole musculoskeletal presentation of systemic juvenile idiopathic arthritis case report	Journal article	Egypt	Case–control	1
Mountain 2025 [7]	The interplay between pain and disease activity: personal models of pain beliefs and emotional representations in children and young people with juvenile idiopathic arthritis in a UK nationwide prospective inception cohort	Journal article	UK	Cross-sectional (CAPS dataset)	187
Naddei 2023 [90]	Determinants of parent/patient overall well-being poor ratings in juvenile idiopathic arthritis patients with inactive disease	Conference abstract/poster	Italy; Ecuador; Colombia; Peru; Costa Rica	Secondary analysis (EPOCA)	3537, 3391 for multivariable analysis
Nguyen 2024 [91]	A decade of progress in juvenile idiopathic arthritis treatments and outcomes in Canada: results from ReACCh-Out and the CAPRI registry	Journal article	Canada	Prospective inception cohort comparison (ReACCh-Out 2005–10 *vs* CAPRI 2017–21)	1849 ReACCh-Out: 1128, CAPRI: 721
Nistala 2007 [92]	Clinical assessment and core outcome variables are poor predictors of hip arthritis diagnosed by MRI in juvenile idiopathic arthritis	Journal article	UK	Retrospective	34
Oen 2009 [93]	Predictors of early inactive disease in a juvenile idiopathic arthritis cohort: results of a Canadian multicentre, prospective inception cohort study	Journal article	Canada	Prospective cohort (ReACCh-Out)	356
Oen 2018 [94]	Health-related quality of life in an inception cohort of children with juvenile idiopathic arthritis: a longitudinal analysis	Journal article	Canada	Prospective cohort (ReACCh-Out)	1249
Oen 2021 [95]	Causal pathways to health-related quality of life in children with juvenile idiopathic arthritis: results from the ReACCh-Out cohort	Journal article	Canada	Longitudinal secondary analysis (ReACCh-Out)	1125
Palmisani 2006 [96]	Correlation between juvenile idiopathic arthritis activity and damage measures in early, advanced, and longstanding disease	Journal article	Italy	Cross-sectional	223
Papsdorf 2011 [97]	Complete control of disease activity and remission induced by treatment with etanercept in juvenile idiopathic arthritis	Journal article	Germany	Prospective, multicentre, open-label observational registry-based cohort study (German Etanercept Registry)	787 (ID), 623 (CRM)
Pedersen 2008 [98]	A prospective study of magnetic resonance and radiographic imaging in relation to symptoms and clinical findings of the temporomandibular joint in children with juvenile idiopathic arthritis	Journal article	Denmark	Prospective longitudinal cohort	15, 10 controls
Pinto 2018 [99]	Temporomandibular joint disorders as the only manifestation of juvenile idiopathic arthritis: a case report	Journal article	Brazil	Case report	1
Portelli 2014 [100]	Temporomandibular joint involvement in a cohort of patients with juvenile idiopathic arthritis and evaluation of the effect induced by functional orthodontic appliance: clinical and radiographic investigation	Journal article	Italy	Cross-sectional and interventional	53, 15 TM joint
Quilis 2024 [101]	Peripheral blood regulatory T cells and disease activity, quality of life, and outcomes in children with juvenile idiopathic arthritis	Journal article	Spain	Two-phase study: cross-sectional and prospective follow-up	87
Radziszewska 2019 [102]	Cytokine signature does not correlate with pain or disease activity in well controlled ERA	Conference abstract/poster	UK	Cross-sectional	14 with ERA, 12 controls
Randell 2024 [103]	Pain interference in juvenile idiopathic arthritis	Journal article	USA	Cross-sectional, observational, part of a multicentre cohort study (CARRA)	355
Rapoff 1991 [104]	The validity and reliability of parental ratings of disease activity in juvenile rheumatoid arthritis	Journal article	USA	Validation	31
Rashid 2018 [105]	Patterns of pain over time among children with juvenile idiopathic arthritis	Journal article	UK	Longitudinal cohort (prospective inception cohort—CAPS)	851
Ravelli 1997 [106]	Correlation between conventional disease activity measures in juvenile chronic arthritis	Journal article	Italy	Cross-sectional	55
Ross 1989 [107]	Validity of reported pain as a measure of clinical state in juvenile rheumatoid arthritis	Journal article	USA	Cross-sectional validation study	101
Rumsey 2020 [108]	Worse quality of life, function, and pain in children with enthesitis, irrespective of their juvenile arthritis category	Journal article	Canada	Secondary analysis of prospective cohort data (ReACCh-Out), longitudinal	1371, 214 ERA
Rypdal 2024 [109]	Disease activity trajectories from childhood to adulthood in the population-based Nordic juvenile idiopathic arthritis cohort	Journal article	Denmark; Finland; Norway; Sweden	Prospective longitudinal cohort study; 18-year follow-up (Nordic JIA cohort)	427
Schanberg 1997 [3]	Pain coping and the pain experience in children with juvenile chronic arthritis	Journal article	USA	Cross-sectional	56
Schanberg 2003 [5]	Daily pain and symptoms in children with polyarticular arthritis	Journal article	USA	Longitudinal	41
Scott 1977 [110]	Measurement of pain in juvenile chronic polyarthritis	Journal article	Canada	Cross-sectional, measurement-focused validation	100
Sgarlat 2011 [111]	Physician *vs* parent/patient assessment of disease activity in a large cohort of patients with juvenile idiopathic arthritis	Conference abstract/poster	USA	Cross-sectional secondary analysis of registry data (CARRAnet)	2618
Sherry 1990 [112]	Painless juvenile rheumatoid arthritis	Journal article	USA	Retrospective case–control cohort study	41
Shiff 2018 [113]	Trajectories of pain severity in juvenile idiopathic arthritis: results from the Research in Arthritis in Canadian Children Emphasizing Outcomes cohort	Journal article	Canada	Prospective longitudinal cohort study with latent trajectory analysis up to 5 years	1062
Shoop-Worrall 2018 [114]	Long-term outcomes following achievement of clinically inactive disease in juvenile idiopathic arthritis: the importance of definition	Journal article	UK	Longitudinal inception cohort analysis (CAPS)	832
Shoop-Worrall 2021 [115]	Patient-reported wellbeing and clinical disease measures over time captured by multivariate trajectories of disease activity in individuals with juvenile idiopathic arthritis in the UK: a multicentre prospective longitudinal study	Journal article	UK	Multicentre prospective longitudinal study	1184
Spadaro 1996 [116]	Interleukin-6 and soluble interleukin-2 receptor in juvenile chronic arthritis: correlations with clinical and laboratory parameters	Journal article	Italy	Cross-sectional	24, 15 controls
Spekking 2023 [117]	Significant pain decrease in children with non-systemic juvenile idiopathic arthritis treated to target: results over 24 months of follow up	Journal article	Netherlands	RCT with longitudinal follow-up over 24 months, multicentre, single-blind, three-arm RCT	92
Stoll 2010 [118]	Spondyloarthritis in a pediatric population: risk factors for sacroiliitis	Journal article	USA	Retrospective	143
Stoustrup 2016 [119]	Temporomandibular pain in patients with juvenile idiopathic arthritis	Conference abstract/poster	Denmark	Cross-sectional	172
Sudhakar 2022 [120]	The reliability of juvenile arthritis magnetic resonance imaging scoring system in the evaluation of the shoulder joint in juvenile idiopathic arthritis	Journal article	India	Cross-sectional observational	20
Svensson 2000 [121]	Temporomandibular disorders in juvenile chronic arthritis patients. A clinical study	Journal article	Sweden	Cross-sectional	105
Svensson 2001 [122]	The mandibular condyle in juvenile chronic arthritis patients with mandibular hypoplasia: a clinical and histological study	Journal article	Sweden	Clinical and histological	12
Tarakci 2011 [123]	The relationship between physical activity level, anxiety, depression, and functional ability in children and adolescents with juvenile idiopathic arthritis	Journal article	Turkey	Cross-sectional	52, 48 controls
Tarkiainen 2018 [124]	Early aggressive treatment decreases pain in patients with juvenile idiopathic arthritis	Conference abstract/poster	Finland	Part of RCT	60
Thastum 2011 [125]	Pain-specific beliefs and pain experience in children with juvenile idiopathic arthritis: a longitudinal study	Journal article	Denmark	Longitudinal	47
Thompson 1987 [126]	Comprehensive assessment of pain in juvenile rheumatoid arthritis: an empirical model	Journal article	USA	Cross-sectional validation	23
Tirelli 2020 [127]	Determinants of disease activity change over time in enthesitis related arthritis: effect of structured outcome monitoring and clinical decision support	Journal article	Canada	Quasi-experimental pre–post cohort (retrospective analysis, ReACCh-Out)	152
Trachtman 2021 [128]	PROMIS computer adaptive tests and their correlation with disease activity in juvenile idiopathic arthritis	Journal article	USA	Cross-sectional	44
Trachtman 2021 [129]	The value of the patient global health assessment in polyarticular juvenile idiopathic arthritis: a nested cohort study	Journal article	USA	Secondary analysis (nested cohort study within a clinical trial)	120
Tripalo Batoš 2020 [130]	Is axial magnetic resonance imaging useful in early juvenile spondyloarthritis—preliminary report	Journal article	Croatia	Cross-sectional	27
Tucker 2009 [131]	Correlates of pain in an inception cohort of children with juvenile idiopathic arthritis (JIA): research in arthritis in Canadian children	Conference abstract/poster	Canada	Prospective longitudinal inception cohort study (ReACCh-Out)	321
Tupper 2013 [132]	Pain intensity variability and its relationship with quality of life in youths with juvenile idiopathic arthritis	Journal article	Canada	Secondary analysis of prospective observational studies	85
Ünal 2018 [133]	Biopsychosocial status of jia patients: perspectives of daily living activities, disease activity and family impact	Conference abstract/poster	Turkey	Cross-sectional	196
Upadhyay 2021 [134]	A multidisciplinary assessment of pain in juvenile idiopathic arthritis	Journal article	USA	Pilot observational	16
Van Der Heijden 2022 [135]	Proteomics based markers of clinical pain severity in juvenile idiopathic arthritis	Journal article	USA	Preliminary and exploratory cross-sectional pilot	15
van Dijkhuizen 2023 [136]	Validity and reliability of four parent/patient-reported outcome measures for juvenile idiopathic arthritis remote monitoring	Journal article	Italy	Validation (EPOCA)	6060, 8643 parents
Vandvik 1990 [137]	Relationship between pain, disease severity and psychosocial function in patients with juvenile chronic arthritis (JCA)	Journal article	Norway	Cross-sectional	57
Varni 1987 [138]	The Varni/Thompson Pediatric Pain Questionnaire. I: Chronic musculoskeletal pain in juvenile rheumatoid arthritis	Journal article	USA	Validation	25
Vega-Fernandez 2023 [12]	Musculoskeletal ultrasound and the assessment of disease activity in juvenile idiopathic arthritis	Journal article	USA; Canada	Prospective cohort (CARRA)	30
Verstappen 2015 [139]	The association between low socioeconomic status with high physical limitations and low illness self-perception in patients with juvenile idiopathic arthritis: results from the Childhood Arthritis Prospective Study	Journal article	UK	Secondary analysis (CAPS), cross-sectional analysis at baseline	923
Viswanath 2011 [140]	Levels of serum matrix metalloproteinase-3 correlate with disease activity in the enthesitis-related arthritis category of juvenile idiopathic arthritis	Journal article	India	Prospective cohort longitudinal	54
von Schuckmann 2020 [141]	Temporomandibular joint involvement in patients with juvenile idiopathic arthritis: a retrospective chart review	Journal article	Germany	Retrospective	2413
Vuorimaa 2008 [142]	Empirical classification of children with JIA: a multidimensional approach to pain and well-being	Journal article	Finland	Cross-sectional with cluster classification	145
Weiss 2012 [143]	Enthesitis-related arthritis is associated with higher pain intensity and poorer health status in comparison with other categories of juvenile idiopathic arthritis: The Childhood Arthritis and Rheumatology Research Alliance Registry	Journal article	USA	Retrospective cross-sectional analysis (CARRA Registry)	2571
Weiss 2016 [31]	Assessment of sacroiliitis at diagnosis of juvenile spondyloarthritis by radiography, magnetic resonance imaging, and clinical examination	Journal article	USA	Prospective cross-sectional	40, 14 controls
Xu 2016 [144]	Potential effects of interleukins on the pathogenesis of systemic onset juvenile idiopathic arthritis	Journal article	China	Cross-sectional	60, 20 controls
Yamazaki 2022 [145]	MRI evaluation of active sacroiliitis in juvenile spondyloarthritis treated with TNF inhibitors	Conference abstract/poster	Japan	Retrospective	7
Yilmaz 2010 [146]	Pelvic MRI findings of juvenile-onset ankylosing spondylitis	Journal article	Turkey	Retrospective	11
Zhang 2025 [147]	Latent class analysis identifies distinct pain phenotypes in newly diagnosed systemic juvenile idiopathic arthritis	Journal article	China	Retrospective cohort	165
Zwir 2012 [148]	Does intense synovial enhancement in temporomandibular joints of juvenile idiopathic arthritis patients correlate with disease activity?	Conference abstract/poster	Brazil	Longitudinal	75

CAPRI: Canadian Alliance of Pediatric Rheumatology Investigators; CAPS: Childhood Arthritis Prospective Study; CARRA: Childhood Arthritis and Rheumatology Research Alliance; CARRAnet: Childhood Arthritis and Rheumatology Research Alliance network; CRM: clinical remission on medication; ERA: enthesitis-related arthritis; EPOCA: Epidemiology, treatment and Outcome of Childhood Arthritis; ID: inactive disease; JCA: juvenile chronic arthritis; PROMS: patient-reported outcome measures; PREMS: patient-reported experience measures; RCT: randomized controlled trial; ReACCh-Out: Research in Arthritis in Canadian Children Emphasizing Outcomes; TMJ: temporomandibular joint.

Most studies examined pain and objective or clinical markers concurrently and analysed relationships using statistical methods, except for imaging-based studies, of which 40% were analysed and reported narratively. Statistical approaches most commonly used group-level association measures (Supplementary Tables S2–S9).

Most studies included mixed JIA subtypes, usually reflecting the full ILAR spectrum. Around one-quarter focused on predominantly single subtype cohorts. Some studies examined specific anatomical regions, most often the temporomandibular or sacroiliac joints. Studies most often included mixed age groups and balanced sex or gender distributions. Race and ethnicity were largely unreported. Detailed participant characteristics are provided in Supplementary Table S1, with study level participant and analytic details presented in Supplementary Table S2.

### Pain measures

Pain intensity was the most commonly assessed pain related construct, most often assessed using a visual analogue scale, with fewer studies using pain interference, location or multidimensional paediatric pain tools. Pain reporters were frequently mixed, whereby children aged ∼8–10 years or older provided self-report and parents reported for younger children. Where specified, recall periods most often reflected current or the previous week, although many studies did not report a recall period. Table 2 summarizes pain constructs, recall periods and reporters across included studies, while study-level pain-marker comparisons and analytic details are presented in Supplementary Table S2.

**Table 2:** Summary of pain measures, recall periods and reporters across included studies.

Category	Subcategory/examples	Count (studies)
Construct		
Intensity		86
	Visual analogue scales (VAS), *n* = 73	
	Numeric rating scale (NRS), *n* = 10	
	Faces Pain Scales (FPS, FPS-R), Oucher, *n* = 3	
Narrative/clinical notes	Binary present/absent, clinician case notes	29
Interference/functional impact	PROMIS-PI, CHAQ-PI, SF-36 PI, GCPS disability	10
Location/distribution	Body map, drawings, specific questionnaires	9
Frequency	Daily diaries, Rapoff 0–2 scale, week/month recall	7
Multidimensional	Pediatric Pain Assessment Tool/Questionnaire (PPAT/PPQ)	5
Variability	AAC	1
Recall period		
Not reported	Recall period unclear or missing	56
Past week	Past week, previous week (average, worst), CHAQ weekly recall	37
Current/present	Current pain during examination/assessment	23
Other	Worst ever, longer recalls periods, diaries over varied time periods	10
Measurement frequency		
Single assessment	Pain recorded once at a single visit	95
Repeated assessment	Pain recorded on more than one occasion (diaries, multiple visits, trajectories)	24
Not reported		3
Reporter		
Mixed	CYP + parent OR age-determined proxy OR CYP + case notes	48
CYP	CYP self-report only	33
Case notes	Clinician-recorded pain (binary or descriptive)	23
Parent/proxy	Parent or caregiver report only	10
Not reported		8

FPS: Faces Pain Scale; FPS-R: Faces Pain Scale–Revised; PROMIS-PI: Patient-Reported Outcomes Measurement Information System, Pain Interference; CHAQ-PI: Childhood Health Assessment Questionnaire, Pain Interference; SF-36 PI: 36-Item Short Form Health Survey, Pain Interference; GCPS: Graded Chronic Pain Scale; PPAT: Pediatric Pain Assessment Tool; PPQ: Pediatric Pain Questionnaire; AAC: average absolute change; CYP: child or young person.

### Objective and clinical markers explored in relation to pain in JIA

Most studies analysed pain in relation to a single disease activity construct (*n* = 85; 70%), most commonly imaging (*n* = 35), PhGA (*n* = 17), composite DAS (e.g. JADAS; *n* = 11), blood biomarkers (*n* = 10) or joint counts, including enthesitis counts (*n* = 8) (Supplementary Table S2). A small number of studies (*n* = 4) examined constructs not otherwise captured within the main categories, including Wallace criteria for inactive disease, joint damage measures and thermography (infrared imaging used to detect skin temperature differences as a proxy for local inflammation).

The remaining studies (*n* = 37; 30%) analysed pain in relation to more than one disease activity marker category (i.e. multi-modal combinations of physician-assessed clinical measures, blood biomarkers and/or imaging). These multi-modal studies most frequently examined pain in relation to multiple physician-assessed clinical measures (most commonly both PhGA and active joint count; *n* = 15), or physician-assessed clinical measures in combination with blood biomarkers (*n* = 11). Few studies examined imaging alongside physician-assessed clinical measures or composite DAS (*n* = 4).

### Relationships between pain and objective or clinical markers of disease activity

When pain-disease activity marker relationships were considered at the study level, 21 of 122 studies (17%) reported overall concordance. Forty-six (38%) reported overall discordance, which included weak or author-reported non-significant associations, persistent pain despite markers indicating low disease activity, or low or absent pain despite evidence of active disease. Fifty-five studies (45%) reported mixed relationships, where concordant and discordant findings co-existed across markers, subgroups or time points. The main reason for mixed findings was pain being analysed against multiple disease activity constructs hence aligning with some markers but not others (49%). Mixed findings also reflected heterogeneity across patient subgroups or pain trajectories (27%), variation in how pain was measured, reported or contextualized (e.g. pain recall period or reporter; 15%), and time or outcome-dependent divergence, where pain and markers of disease activity aligned at some assessments but not others (e.g. prior to flare *vs* during flare; 9%).

### Disease activity marker–specific findings

Findings were synthesized for each category of objective or clinical disease activity marker. Analyses from studies that used multiple markers were disaggregated, so the total number of analyses per marker type exceeds the number of single-marker studies. Marker-specific analyses were classified independently of overall study-level concordance, such that studies with mixed overall findings could be classified as concordant or discordant for individual markers. Detailed marker-specific extraction tables are provided in Supplementary Tables S3–S9, which present individual pain–marker analyses, with study-level characteristics retained for context. Patterns of concordance varied substantially by marker category (Fig. 2).

**Figure 2:**
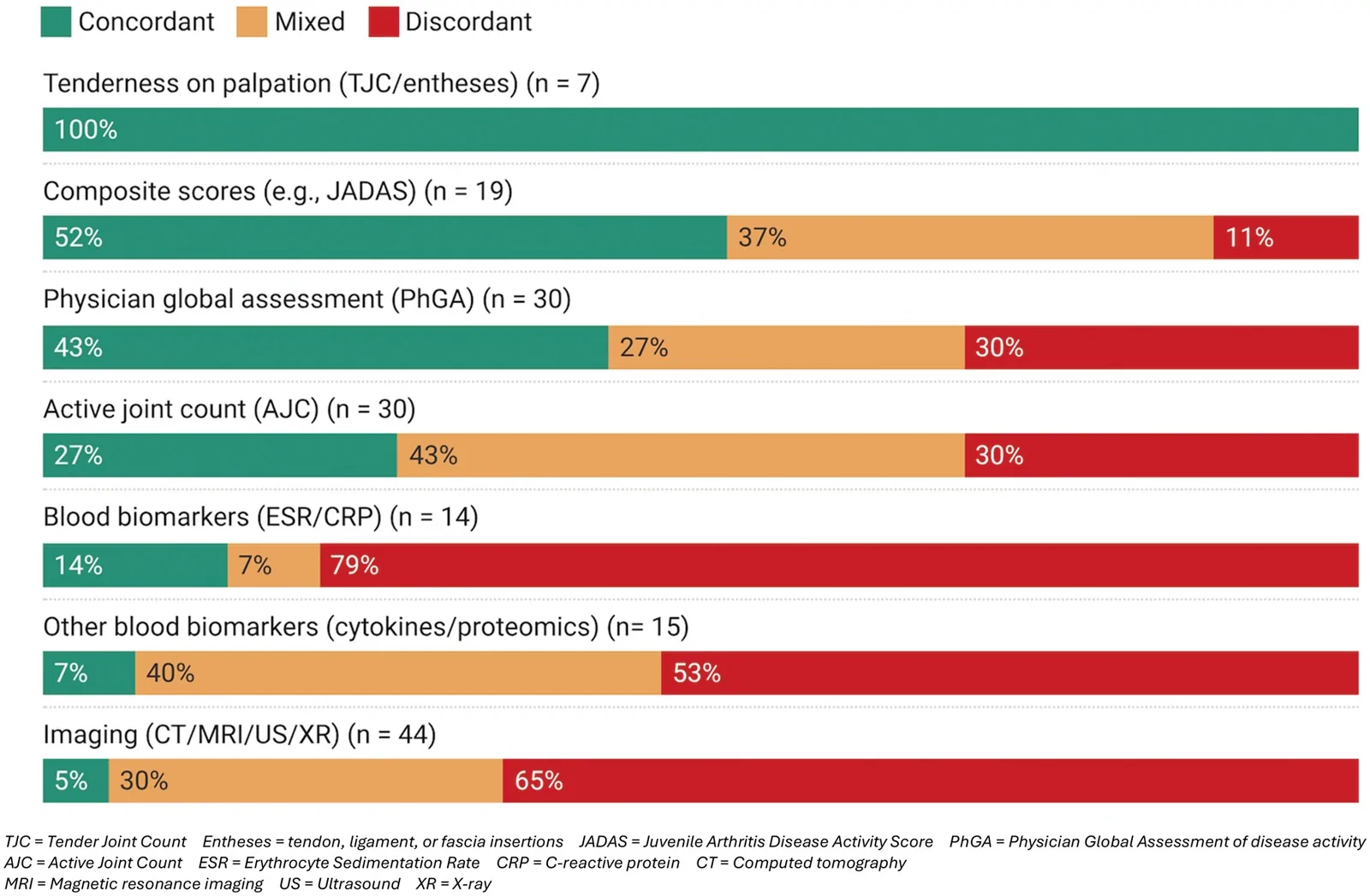
Distribution of concordant, mixed and discordant findings across marker analyses. Bars show the proportion of each relationship type within each marker category

#### Physician-assessed clinical measures and composite DAS

Studies that used physician-assessed measures or composite DAS predominantly included mixed JIA subtypes and assessed generalized/whole-body pain or disease activity (Supplementary Tables S3–S6).

Two studies examined pain in relation to joint damage using the Juvenile Arthritis Damage Index (JADI-A and JADI-E); neither identified an association between pain and joint damage (Supplementary Table S2).

#### Imaging and laboratory biomarkers

Imaging-based studies were more likely to have included single JIA subtypes [35%; most commonly enthesitis-related arthritis (ERA) and juvenile SpA] and focused on the TM joint (40%), followed by the SI joint (20%) and spine (11%), with few studies examining peripheral joints or whole-body imaging. Across all imaging modalities, there were concordant findings in only 5% of analyses. Discordance most often reflected imaging findings with low or absent pain (65%).

Studies analysing blood biomarkers also included a higher proportion of single-subtype cohorts (30%; most commonly systemic juvenile idiopathic arthritis (sJIA) or ERA). ESR and CRP were the least concordant with pain (79% discordant), with most discordant findings reflecting a lack of association. Relationships between pain and other blood biomarkers, including cytokines, immune markers and proteomic panels were more heterogeneous, but no single biomarker demonstrated consistent concordance across studies (Supplementary Table S9).

Given that fewer than one in five studies reported overall concordance, and that mixed findings were common due to multiple disease activity markers being analysed, subsequent synthesis focused on characterizing patterns of discordance.

### Discordance

Among the 46 studies classified as discordant overall, 29 (63%) reported null findings, represented by weak correlations, or author reported lack of statistical significance. These findings occurred across all marker types: imaging, physician-assessed or composite DAS, and blood biomarkers (and multi-modal combinations). The remaining 17 studies reported directional discordance (i.e. high pain despite low disease activity markers or low or absent pain despite evidence of disease activity markers).

To explore patterns of directional discordance, studies reporting directional discordance within otherwise mixed findings were included alongside studies reporting overall discordance. Studies reporting high or persistent pain despite markers indicating low disease activity relied almost exclusively on physician-assessed clinical or composite DAS. In contrast, studies reporting low or absent pain predominantly used imaging markers of disease activity, either alone or in combination with other measures, reflecting differences in marker selection and clinical context rather than diagnostic performance (Supplementary Table S10).

Eight studies explicitly quantified directional discordance at the individual level using pre-specified criteria or subgroup definitions (Supplementary Table S2). Persistent pain despite markers indicating low or inactive disease activity was reported in five studies, with authors reporting that 7–22% of children and young people experienced ongoing pain despite physician- or composite score–determined low disease activity (high pain–low disease activity marker discordance). Conversely, a smaller number of studies quantified the opposite pattern, where low or absent pain occurred despite objective markers indicating active disease (low pain–high disease activity marker discordance), most commonly identified using imaging markers of subclinical inflammation, particularly MRI and US.

### Predictive studies

Eight longitudinal studies examined predictive relationships (Supplementary Table S2). Authors reported that early pain was weakly predictive of subsequent disease activity, while baseline disease activity markers showed weak or inconsistent associations with later pain, except for enthesitis in one study [26].

### Exploratory subgroup analyses

Exploratory analyses examined whether pain-disease activity marker relationships varied by participant or disease characteristics (age group, sex/gender, JIA subtype, disease status or duration, and anatomical region) or by pain measurement characteristics (construct, reporter, recall period or measurement frequency). Studies including adolescents (13–17 years; *n* = 16) more frequently reported discordant relationships (63%). Studies focusing on ERA (*n* = 8), sJIA (*n* = 6) and the TM joint (*n* = 17) were predominantly discordant, with no studies reporting overall concordance. Other age groups and polyarticular JIA studies broadly reflected the overall pattern observed across all studies. Interpretation of subgroup findings is limited by small sample sizes and systematic differences in marker selection; for example, adolescent-, ERA-, sJIA- and TM joint–focused studies more often relied on imaging or blood biomarker-based assessments, which demonstrated lower overall concordance with pain. Studies using a single pain assessment reported fewer concordant relationships than those incorporating repeated pain measurements (14% *vs* 30%). No consistent patterns were identified in relation to sex or gender, disease status, disease duration or other pain measurement characteristics.

## Discussion

This scoping review synthesized data from over 57 000 children and young people with JIA across 122 studies examining the relationship between pain and objective or clinical markers used to approximate disease activity in CYP with JIA. Fewer than one in five studies reported overall concordance at the study level. Pain aligned more consistently with physician-assessed clinical measures and composite scores than with imaging modalities or laboratory inflammatory biomarkers when examined in marker-specific analyses.

Studies examining physician-assessed clinical measures and composite DAS reported the highest proportion of concordant findings. However, several methodological considerations are relevant when interpreting these findings. The PhGA does not include a patient-reported component but may be influenced by subjective patient pain reports and shows substantial inter-rater variability [27, 28]. Composite DAS (e.g. JADAS) incorporate a parent or patient global assessment, a subjective component that has been shown to correlate with pain intensity and may function as a proxy for pain [23]. As a result, concordance with pain may partly reflect shared construct content rather than independent alignment with inflammatory disease activity.

In contrast, studies that explored the relationship between pain and imaging modalities frequently reported imaging findings without associated pain. While normal developmental imaging features in children can mimic inflammatory pathology and require cautious interpretation [29], symptoms and routine examination can underestimate joint involvement in regions known to be clinically silent, such as the TM joint and SI joint [30, 31]. Imaging-based studies often focused on these anatomical sites, which may partly explain the poor concordance and limit broader interpretation of pain–imaging relationships. Most studies that explored pain in relation to ESR or CRP reported weak or non-significant associations, consistent with findings from RA in adults [32].

Across all studies, discordance most commonly reflected weak or non-significant associations between pain and disease activity markers. Most studies relied on single, one-off pain assessments, typically reflecting current or past-week pain, which does not reflect routine clinical practice, where pain is often discussed in relation to experiences over longer intervals between visits. When discordance was explicitly reported as directional (i.e. pain levels that were high or low relative to markers of disease activity), this occurred in different clinical contexts and using different marker types. In studies that reported ongoing or high pain despite low disease activity, disease activity was typically defined using physician-assessed clinical measures alone (i.e. no imaging) and interpretations more often emphasized psychosocial or non-inflammatory contributors. In contrast, studies identifying low or absent pain despite evidence of disease activity were predominantly imaging-based. The identification and interpretation of directional discordance in these studies may therefore have been influenced by whether imaging was incorporated into assessment, rather than reflecting inherent differences in pain–disease activity relationships.

These findings of this review highlight several important clinical considerations. Discordance between pain and markers of disease activity is common and clinicians should therefore explicitly discuss the potential for discordance with patients. When current disease activity markers suggest low or inactive disease, pain experiences should be validated to prevent epistemic injustice (i.e. harm arising when a patient’s capacity as a knower is unfairly diminished) and stigma, potentially shaping coping strategies and engagement with care [33]. Conversely, low or absent pain may be misinterpreted by children and families as remission, potentially reducing treatment adherence [34].

Pain is complex, may be multifactorial and reflect central nervous system alterations or psychosocial factors [35, 36], challenging the biomedical model often prioritized by clinicians [8]. However, studies in this review that quantified persistent pain in the absence of active disease in JIA relied primarily on physician-assessed clinical measures or composite scores alone. This represents an important limitation, given evidence that physical examination has restricted reliability and that imaging may be warranted in atypical or discordant presentations [29]. Incorporating imaging alongside clinical or composite disease activity measures in future studies may help clarify the extent to which inflammatory or structural abnormalities not otherwise evident on clinical examination may be present in children with pain despite low or inactive disease.

This review has limitations that should be considered when interpreting findings. As a scoping review, no formal appraisal of study quality was undertaken, consistent with JBI guidance, and the aim was to map the extent and nature of the evidence rather than evaluate risk of bias. The review included conference abstracts, allowing broader mapping of emerging evidence, although findings from these sources should be interpreted cautiously given their brief and non-peer-reviewed nature. Finally, this review maps relationships between pain and markers used to approximate disease activity rather than providing direct evidence of inflammatory activity. Use of established JBI methodology and reporting in accordance with PRISMA-ScR represents a methodological strength.

In conclusion, relationships between pain and disease activity markers in JIA are complex, often non-linear, and frequently discordant, reflecting both the subjective nature of pain and limitations in how disease activity is currently assessed. These findings have important implications for clinicians and consumers. Pain may be a source of anxiety for CYP with JIA, particularly when its relationship to underlying disease activity is uncertain [9]. For clinicians, patient-reported pain may be de-prioritized when clinical or laboratory assessments do not indicate active disease [8]. Our findings highlight that pain and markers used to approximate disease activity in JIA should be interpreted in parallel rather than as interchangeable indicators. Guidance supporting a more consistent approach to interpreting and integrating pain and disease activity markers would assist both clinicians and consumers.

## Supplementary Material

keag544_Supplementary_Data

## Data Availability

Data supporting the findings of this review are included in the article and online supplementary material. Additional extracted data are available from the corresponding author upon reasonable request.
