# Supplementary material for: Exploring the relationship between pain and markers of disease activity in juvenile idiopathic arthritis: a scoping review
Source: Rheumatology (Oxford). 2026 Sep 29;65(10):keag544. doi: 10.1093/rheumatology/keag544 (PMC13704791; doi:10.1093/rheumatology/keag544)
Supplement: Supplementary file 1 [file keag544_supplementary_data.docx]

**Supplementary Data S1**

**Search histories (12 June 2025)**

MEDLINE (Ovid)

| **#** | **Query** | **Results from 12 Jun 2025** |
| --- | --- | --- |
| 1 | "Arthritis, Juvenile"/ | 12,365 |
| 2 | ((juvenil* or child*) adj3 arthriti*).ti,ab,kf. | 14,527 |
| 3 | (juvenil* adj2 arthropath*).ti,ab,kf. | 56 |
| 4 | (JIA or JRA or JCA or "S JIA" or SJIA or "SO JIA" or SOJIA or SJIA or PCJIA or JRD or OJIA or OLIOGOJIA or JUSPA or JSPA or JAS).ti,ab,kf. | 9,189 |
| 5 | or/1-4 | 19,329 |
| 6 | Infant/ | 908,372 |
| 7 | "Child, Preschool"/ | 1,038,359 |
| 8 | Child/ | 2,026,393 |
| 9 | Adolescent/ | 2,334,401 |
| 10 | Young Adult/ | 1,140,965 |
| 11 | Pediatrics/ | 59,964 |
| 12 | (infant or infants or baby or babies or toddler* or child* or school age* or schoolage* or schoolchild* or schoolboy* or schoolgirl* or school going or schoolgoing or preschool* or "pre school*" or kindergarten* or (school and student*) or kid or kids or underage* or youth or youths or adolescen* or juvenil* or minors or boy or boys or girl or girls or teen* or preteen* or preadolescen* or prepubescent* or pubescent* or preteen* or prepuberty or puber* or "under 18" or "under 18s" or pediatr* or paediatr* or peadiatr* or (young* adj2 (person* or people* or adult* or individual* or woman* or women* or man* or men* or patient or patients or population*))).ti,ab,kf. | 3,224,897 |
| 13 | (pediatr* or paediatr* or child* or adolescen*).jw. | 895,311 |
| 14 | or/6-13 | 5,664,607 |
| 15 | "Arthritis, Psoriatic"/ | 9,173 |
| 16 | "Arthritis, Rheumatoid"/ | 115,738 |
| 17 | Sacroiliitis/ | 852 |
| 18 | Spondylarthritis/ | 4,163 |
| 19 | "Spondylitis, Ankylosing"/ | 17,289 |
| 20 | Spondylarthropathies/ | 1,420 |
| 21 | (polyarthriti* or polyarticular or oligoarthriti* or oligoarticular or pauciarth* or pauciarticular or poly arthriti* or poly articular or oligo arthriti* or oligo articular or pauci arth* or pauci articular or (arthr* adj2 (psoria* or iliosacral or sacroiliac)) or rheumatoid arthritis or rheumatic arthritis or sacroiliiti* or sacroileitis or sacroilitis or spondyloarthr* or spondylarthr* or spondylo arthr* or ((spin* or vertebral) adj arthrit*) or rheumatoid spondylitis or (spondylitis adj2 ankylo*) or rheumatic disease* or inflammatory arthr* or enthesitis or still* disease or still* syndrome or still chauffard* disease).ti,ab,kf. | 196,421 |
| 22 | or/15-21 | 222,744 |
| 23 | Pain/ | 161,715 |
| 24 | Pain Measurement/ | 101,342 |
| 25 | Chronic Pain/ | 27,265 |
| 26 | Acute Pain/ | 3,571 |
| 27 | Facial Pain/ | 7,612 |
| 28 | Nociceptive Pain/ | 1,016 |
| 29 | Pain Threshold/ | 14,901 |
| 30 | Pain Management/ | 45,285 |
| 31 | Pain Perception/ | 3,521 |
| 32 | Hyperalgesia/ | 15,068 |
| 33 | Central Nervous System Sensitization/ | 1,152 |
| 34 | Visual Analog Scale/ | 4,222 |
| 35 | Hypersensitivity/ | 53,773 |
| 36 | (pain* or hurt* or discomfort* or physical* suffer* or aching or ache* or tender* or visual analog* scale or VAS or nocicept* or nocipercept* or hyperalg* or hyper alg* or hyperpathia or allodynia* or enthesalgi* or ((central or CNS) adj4 sensiti*) or somatosensory profile* or hypersensitiv* or hyper sensitiv*).ti,ab,kf. | 1,207,268 |
| 37 | or/23-36 | 1,302,844 |
| 38 | Biomarkers/ | 387,074 |
| 39 | Blood Sedimentation/ | 12,716 |
| 40 | "C-Reactive Protein"/ | 58,676 |
| 41 | Cytokines/ | 192,355 |
| 42 | "Glial Cell Line-Derived Neurotrophic Factor"/ | 3,925 |
| 43 | Hematologic Tests/ | 10,392 |
| 44 | Joints/dg | 988 |
| 45 | Magnetic Resonance Imaging/ | 512,125 |
| 46 | Mass Spectrometry/ | 111,985 |
| 47 | "Liquid Chromatography-Mass Spectrometry"/ | 1,610 |
| 48 | Physical Examination/ | 44,490 |
| 49 | Proteomics/ | 79,641 |
| 50 | Proteome/ | 42,999 |
| 51 | Radiography/ | 330,808 |
| 52 | "Range of Motion, Articular"/ | 64,440 |
| 53 | "Severity of Illness Index"/ | 284,507 |
| 54 | Synovial Fluid/ | 14,205 |
| 55 | Thermography/ | 8,452 |
| 56 | "T-Lymphocytes, Regulatory"/ | 39,661 |
| 57 | Ultrasonography/ | 209,514 |
| 58 | "Ultrasonography, Doppler"/ | 17,911 |
| 59 | ((biologic* or physiologic* or objective) adj3 (measure* or indicator* or marker*)).ti,ab,kf. | 92,703 |
| 60 | (biomarker* or ((biochemical or serum) adj2 marker*) or (lab* adj2 (test* or marker*)) or protein* or cytokine* or interleukin* or "c reactive" or crp or hscrp).ti,ab,kf. | 4,775,710 |
| 61 | (((blood or erythrocyte) adj2 sedimentation) or ESR or ((blood or hematologic or haematologic* or serolog*) adj2 test*)).ti,ab,kf. | 113,922 |
| 62 | ((glial adj4 neurotrophic factor) or GDNF).ti,ab,kf. | 6,743 |
| 63 | (swelling or swollen or effusion or arthrocele or arthroncus).ti,ab,kf. | 192,888 |
| 64 | (joint adj (examination or count* or evaluat* or assess*)).ti,ab,kf. | 4,209 |
| 65 | (imaging* or magnetic resonance image* or MRI or magnetic resonance tomography or zeugmatography or mr tomography or nmr tomography or proton spin tomography or FMRI or functional mris or functionalmri* or contrast enhancement or radiolog*).ti,ab,kf. | 1,636,831 |
| 66 | (mass spectr* or "LC MS" or "HPLC MS" or "UPLC MS").ti,ab,kf. | 430,896 |
| 67 | ((clinical or physical) adj4 (exam* or evaluat* or assess* or finding*)).ti,ab,kf. | 778,172 |
| 68 | (clinical adj3 (marker* or measure* or data or record* or indicator*)).ti,ab,kf. | 353,097 |
| 69 | (((global assessment or global health assessment) and physician*) or PHGA).ti,ab,kf. | 3,250 |
| 70 | (abnormal* adj2 physical).ti,ab,kf. | 1,701 |
| 71 | sens* test*.ti,ab,kf. | 20,198 |
| 72 | (proteom* or prote* omic* or peptidom*).ti,ab,kf. | 165,369 |
| 73 | (radiograph* or roentgenog* or xray* or "x ray*").ti,ab,kf. | 769,274 |
| 74 | ((motion adj3 (limit* or restric* or range)) or ((joint* or articular) adj3 (flex* or ROM))).ti,ab,kf. | 62,302 |
| 75 | (((((health or disease) adj (activity or course or status)) or (illness or severity)) adj3 (score* or scale* or measur* or marker* or indicat* or classif* or index* or assessment*)) or JADAS or CJADAS).ti,ab,kf. | 159,118 |
| 76 | (((synovial or synovium or articular or joint*) adj3 (hypertroph* or fluid or thicken* or erosion or hyperplasia)) or synovia).ti,ab,kf. | 21,746 |
| 77 | (cartilage adj3 damag*).ti,ab,kf. | 6,496 |
| 78 | (thermograph* or thermoscan* or thermo scan* or thermometry or thermolog* or thermogram* or infrared scanning or IRT or ((thermal or temperature) adj3 (joint* or mapping or imag* or video* or scan*))).ti,ab,kf. | 28,761 |
| 79 | ("t cell*" or "t regulatory" or treg* or "t lymphocyte*" or "tr1 cell*" or "th3 cell*" or "t helper 3 cell*").ti,ab,kf. | 550,614 |
| 80 | (ultrasound* or ultrason* or echograph* or echogram* or echoscop* or echosound or echotomograph* or sonograph* or sonogram* or doppler).ti,ab,kf. | 626,328 |
| 81 | ((cortical or subcortical or cortex) adj3 (volume* or alteration* or thickness or thicken*)).ti,ab,kf. | 22,190 |
| 82 | or/38-81 | 9,731,035 |
| 83 | 5 or (14 and 22) | 40,501 |
| 84 | 37 and 82 and 83 | 3,694 |
| 85 | limit 84 to english language | 3,294 |

Embase (Ovid)

| **#** | **Query** | **Results from 12 Jun 2025** |
| --- | --- | --- |
| 1 | exp juvenile rheumatoid arthritis/ | 30,396 |
| 2 | ((juvenil* or child*) adj3 arthriti*).ti,ab,kf. | 25,865 |
| 3 | (juvenil* adj2 arthropath*).ti,ab,kf. | 75 |
| 4 | (JIA or JRA or JCA or "S JIA" or SJIA or "SO JIA" or SOJIA or SJIA or PCJIA or JRD or OJIA or OLIOGOJIA or JUSPA or JSPA or JAS).ti,ab,kf. | 18,701 |
| 5 | or/1-4 | 38,551 |
| 6 | infant/ | 914,702 |
| 7 | preschool child/ | 749,921 |
| 8 | child/ | 2,682,089 |
| 9 | adolescent/ | 2,124,750 |
| 10 | young adult/ | 629,263 |
| 11 | pediatrics/ | 110,659 |
| 12 | (infant or infants or baby or babies or toddler* or child* or school age* or schoolage* or schoolchild* or schoolboy* or schoolgirl* or school going or schoolgoing or preschool* or "pre school*" or kindergarten* or (school and student*) or kid or kids or underage* or youth or youths or adolescen* or juvenil* or minors or boy or boys or girl or girls or teen* or preteen* or preadolescen* or prepubescent* or pubescent* or preteen* or prepuberty or puber* or "under 18" or "under 18s" or pediatr* or paediatr* or peadiatr* or (young* adj2 (person* or people* or adult* or individual* or woman* or women* or man* or men* or patient or patients or population*))).ti,ab,kf. | 4,486,437 |
| 13 | (pediatr* or paediatr* or child* or adolescen*).jw. | 1,219,621 |
| 14 | or/6-13 | 6,588,831 |
| 15 | psoriatic arthritis/ | 36,218 |
| 16 | rheumatoid arthritis/ | 264,807 |
| 17 | sacroiliitis/ | 6,709 |
| 18 | spondylarthritis/ | 13,844 |
| 19 | ankylosing spondylitis/ | 39,159 |
| 20 | spondyloarthropathy/ | 6,570 |
| 21 | (polyarthriti* or polyarticular or oligoarthriti* or oligoarticular or pauciarth* or pauciarticular or poly arthriti* or poly articular or oligo arthriti* or oligo articular or pauci arth* or pauci articular or (arthr* adj2 (psoria* or iliosacral or sacroiliac)) or rheumatoid arthritis or rheumatic arthritis or sacroiliiti* or sacroileitis or sacroilitis or spondyloarthr* or spondylarthr* or spondylo arthr* or ((spin* or vertebral) adj arthrit*) or rheumatoid spondylitis or (spondylitis adj2 ankylo*) or rheumatic disease* or inflammatory arthr* or enthesitis or still* disease or still* syndrome or still chauffard* disease).ti,ab,kf. | 328,899 |
| 22 | or/15-21 | 404,182 |
| 23 | pain/ | 457,400 |
| 24 | pain measurement/ | 13,986 |
| 25 | pain severity/ | 19,999 |
| 26 | pain assessment/ | 106,552 |
| 27 | chronic pain/ | 94,890 |
| 28 | face pain/ | 15,199 |
| 29 | nociceptive pain/ | 2,922 |
| 30 | pain threshold/ | 23,941 |
| 31 | analgesia/ | 191,854 |
| 32 | nociception/ | 56,734 |
| 33 | exp hyperalgesia/ | 29,254 |
| 34 | central nervous system sensitization/ | 1,062 |
| 35 | visual analog scale/ | 156,061 |
| 36 | tender joint count/ | 1,698 |
| 37 | hypersensitivity/ | 63,816 |
| 38 | (pain* or hurt* or discomfort* or physical* suffer* or aching or ache* or tender* or visual analog* scale or VAS or nocicept* or nocipercept* or hyperalg* or hyper alg* or hyperpathia or allodynia* or enthesalgi* or ((central or CNS) adj4 sensiti*) or somatosensory profile* or hypersensitiv* or hyper sensitiv*).ti,ab,kf. | 1,884,910 |
| 39 | or/23-38 | 2,107,296 |
| 40 | biological marker/ | 575,784 |
| 41 | laboratory test/ | 242,975 |
| 42 | erythrocyte sedimentation rate/ | 79,171 |
| 43 | "C reactive protein"/ | 313,302 |
| 44 | cytokine/ | 314,508 |
| 45 | glial cell line derived neurotrophic factor/ | 11,081 |
| 46 | blood examination/ | 22,494 |
| 47 | blood analysis/ | 101,093 |
| 48 | nuclear magnetic resonance imaging/ | 1,148,775 |
| 49 | mass spectrometry/ | 345,194 |
| 50 | liquid chromatography-mass spectrometry/ | 121,009 |
| 51 | physical examination/ | 337,331 |
| 52 | clinical assessment/ | 266,462 |
| 53 | clinical indicator/ | 7,705 |
| 54 | exp Juvenile Arthritis Disease Activity Score/ | 199 |
| 55 | joint examination/ | 1,152 |
| 56 | joint swelling/ | 11,959 |
| 57 | quantitative sensory testing/ | 2,583 |
| 58 | proteomics/ | 145,700 |
| 59 | radiography/ | 305,526 |
| 60 | "range of motion"/ | 81,747 |
| 61 | "severity of illness index"/ | 30,297 |
| 62 | synovial fluid/ | 25,386 |
| 63 | disease severity assessment/ | 13,454 |
| 64 | thermography/ | 18,648 |
| 65 | regulatory T lymphocyte/ | 109,408 |
| 66 | echography/ | 430,261 |
| 67 | Doppler ultrasonography/ | 47,283 |
| 68 | "cortical thickness (brain)"/ | 9,132 |
| 69 | ((biologic* or physiologic* or objective) adj3 (measure* or indicator* or marker*)).ti,ab,kf. | 137,617 |
| 70 | (biomarker* or ((biochemical or serum) adj2 marker*) or (lab* adj2 (test* or marker*)) or protein* or cytokine* or interleukin* or "c reactive" or crp or hscrp).ti,ab,kf. | 6,162,538 |
| 71 | (((blood or erythrocyte) adj2 sedimentation) or ESR or ((blood or hematologic or haematologic* or serolog*) adj2 test*)).ti,ab,kf. | 206,940 |
| 72 | ((glial adj4 neurotrophic factor) or GDNF).ti,ab,kf. | 8,899 |
| 73 | (swelling or swollen or effusion or arthrocele or arthroncus).ti,ab,kf. | 298,669 |
| 74 | (joint adj (examination or count* or evaluat* or assess*)).ti,ab,kf. | 9,953 |
| 75 | (imaging* or magnetic resonance image* or MRI or magnetic resonance tomography or zeugmatography or mr tomography or nmr tomography or proton spin tomography or FMRI or functional mris or functionalmri* or contrast enhancement or radiolog*).ti,ab,kf. | 2,457,536 |
| 76 | (mass spectr* or "LC MS" or "HPLC MS" or "UPLC MS").ti,ab,kf. | 527,948 |
| 77 | ((clinical or physical) adj4 (exam* or evaluat* or assess* or finding*)).ti,ab,kf. | 1,273,439 |
| 78 | (clinical adj3 (marker* or measure* or data or record* or indicator*)).ti,ab,kf. | 595,725 |
| 79 | (((global assessment or global health assessment) and physician*) or PHGA).ti,ab,kf. | 8,024 |
| 80 | (abnormal* adj2 physical).ti,ab,kf. | 2,903 |
| 81 | sens* test*.ti,ab,kf. | 31,351 |
| 82 | (proteom* or prote* omic* or peptidom*).ti,ab,kf. | 213,639 |
| 83 | (radiograph* or roentgenog* or xray* or "x ray*").ti,ab,kf. | 983,432 |
| 84 | ((motion adj3 (limit* or restric* or range)) or ((joint* or articular) adj3 (flex* or ROM))).ti,ab,kf. | 80,718 |
| 85 | (((((health or disease) adj (activity or course or status)) or (illness or severity)) adj3 (score* or scale* or measur* or marker* or indicat* or classif* or index* or assessment*)) or JADAS or CJADAS).ti,ab,kf. | 264,920 |
| 86 | (((synovial or synovium or articular or joint*) adj3 (hypertroph* or fluid or thicken* or erosion or hyperplasia)) or synovia).ti,ab,kf. | 33,057 |
| 87 | (cartilage adj3 damag*).ti,ab,kf. | 9,890 |
| 88 | (thermograph* or thermoscan* or thermo scan* or thermometry or thermolog* or thermogram* or infrared scanning or IRT or ((thermal or temperature) adj3 (joint* or mapping or imag* or video* or scan*))).ti,ab,kf. | 33,842 |
| 89 | ("t cell*" or "t regulatory" or treg* or "t lymphocyte*" or "tr1 cell*" or "th3 cell*" or "t helper 3 cell*").ti,ab,kf. | 786,971 |
| 90 | (ultrasound* or ultrason* or echograph* or echogram* or echoscop* or echosound or echotomograph* or sonograph* or sonogram* or doppler).ti,ab,kf. | 954,941 |
| 91 | ((cortical or subcortical or cortex) adj3 (volume* or alteration* or thickness or thicken*)).ti,ab,kf. | 34,291 |
| 92 | or/40-91 | 13,511,222 |
| 93 | 5 or (14 and 22) | 65,377 |
| 94 | 39 and 92 and 93 | 7,678 |
| 95 | limit 94 to english language | 7,150 |

Scopus

( TITLE-ABS-KEY ( ( ( juvenil* OR child* ) W/2 arthriti* ) OR ( juvenil* W/1 arthropath* ) OR ( jia OR jra OR jca OR "S JIA" OR sjia OR "SO JIA" OR sojia OR sjia OR pcjia OR jrd OR ojia OR oliogojia OR juspa OR jspa OR jas ) ) ) OR ( ( TITLE-ABS-KEY ( ( infant OR infants OR baby OR babies OR toddler* OR child* OR "school age*" OR schoolage* OR schoolchild* OR schoolboy* OR schoolgirl* OR "school going" OR schoolgoing OR preschool* OR "pre school*" OR kindergarten* OR ( school AND student* ) OR kid OR kids OR underage* OR youth OR youths OR adolescen* OR juvenil* OR minors OR boy OR boys OR girl OR girls OR teen* OR preteen* OR preadolescen* OR prepubescent* OR pubescent* OR preteen* OR prepuberty OR puber* OR "under 18" OR "under 18s" OR pediatr* OR paediatr* OR peadiatr* OR ( young* W/1 ( person* OR people* OR adult* OR individual* OR woman* OR women* OR man* OR men* OR patient OR patients OR population* ) ) ) ) OR SRCTITLE ( pediatr* OR paediatr* OR child* OR adolescen* ) ) AND ( TITLE-ABS-KEY ( ( polyarthriti* OR polyarticular OR oligoarthriti* OR oligoarticular OR pauciarth* OR pauciarticular OR "poly arthriti*" OR "poly articular" OR "oligo arthriti*" OR "oligo articular" OR "pauci arth*" OR "pauci articular" OR ( arthr* W/1 ( psoria* OR iliosacral OR sacroiliac ) ) OR "rheumatoid arthritis" OR "rheumatic arthritis" OR sacroiliiti* OR sacroileitis OR sacroilitis OR spondyloarthr* OR spondylarthr* OR "spondylo arthr*" OR ( ( spin* OR vertebral ) PRE/0 arthrit* ) OR "rheumatoid spondylitis" OR ( spondylitis W/1 ankylo* ) OR "rheumatic disease*" OR "inflammatory arthr*" OR enthesitis OR "still* disease" OR "still* syndrome" OR "still chauffard* disease" ) ) ) ) AND (TITLE-ABS-KEY ( ( pain* OR hurt* OR discomfort* OR "physical* suffer*" OR aching OR ache* OR tender* OR "visual analog* scale" OR vas OR nocicept* OR nocipercept* OR hyperalg* OR "hyper alg*" OR hyperpathia OR allodynia* OR enthesalgi* OR ( ( central OR cns ) W/3 sensiti* ) OR "somatosensory profile*" OR hypersensitiv* OR "hyper sensitiv*" ) )) AND (TITLE-ABS-KEY ( ( ( biologic* OR physiologic* OR objective ) W/2 ( measure* OR indicator* OR marker* ) ) OR ( biomarker* OR ( ( biochemical OR serum ) W/1 marker* ) OR ( lab* W/1 ( test* OR marker* ) ) OR protein* OR cytokine* OR interleukin* OR "c reactive" OR crp OR hscrp ) OR ( ( ( blood OR erythrocyte ) W/1 sedimentation ) OR esr OR ( ( blood OR hematologic OR haematologic* OR serolog* ) W/1 test* ) ) OR ( ( glial W/3 "neurotrophic factor" ) OR gdnf ) OR ( swelling OR swollen OR effusion OR arthrocele OR arthroncus ) OR ( joint PRE/0 ( examination OR count* OR evaluat* OR assess* ) ) OR ( imaging* OR "magnetic resonance image*" OR mri OR "magnetic resonance tomography" OR zeugmatography OR "mr tomography" OR "nmr tomography" OR "proton spin tomography" OR fmri OR "functional mris" OR functionalmri* OR "contrast enhancement" OR radiolog* ) OR ( mass AND spectr* OR "LC MS" OR "HPLC MS" OR "UPLC MS" ) OR ( ( clinical OR physical ) W/3 ( exam* OR evaluat* OR assess* OR finding* ) ) OR ( clinical W/2 ( marker* OR measure* OR data OR record* OR indicator* ) ) OR ( ( ( "global assessment" OR "global health assessment" ) AND physician* ) OR phga ) OR ( abnormal* W/1 physical ) OR "sens* test*" OR ( proteom* OR "prote* omic*" OR peptidom* ) OR ( radiograph* OR roentgenog* OR xray* OR "x ray*" ) OR ( ( motion W/2 ( limit* OR restric* OR range ) ) OR ( ( joint* OR articular ) W/2 ( flex* OR rom ) ) ) OR ( ( ( ( ( health OR disease ) PRE/0 ( activity OR course OR status ) ) OR ( illness OR severity ) ) W/2 ( score* OR scale* OR measur* OR marker* OR indicat* OR classif* OR index* OR assessment* ) ) OR jadas OR cjadas ) OR ( ( ( synovial OR synovium OR articular OR joint* ) W/2 ( hypertroph* OR fluid OR thicken* OR erosion OR hyperplasia ) ) OR synovia ) OR ( cartilage W/2 damag* ) OR ( thermograph* OR thermoscan* OR "thermo scan*" OR thermometry OR thermolog* OR thermogram* OR "infrared scanning" OR irt OR ( ( thermal OR temperature ) W/2 ( joint* OR mapping OR imag* OR video* OR scan* ) ) ) OR ( "t cell*" OR "t regulatory" OR treg* OR "t lymphocyte*" OR "tr1 cell*" OR "th3 cell*" OR "t helper 3 cell*" ) OR ( ultrasound* OR ultrason* OR echograph* OR echogram* OR echoscop* OR echosound OR echotomograph* OR sonograph* OR sonogram* OR doppler ) OR ( ( cortical OR subcortical OR cortex ) W/2 ( volume* OR alteration* OR thickness OR thicken* ) ) )) AND ( LIMIT-TO ( LANGUAGE , "English" ) )

**Supplementary Data S2**

**Preferred Reporting Items for Systematic reviews and Meta-Analyses extension for Scoping Reviews (PRISMA-ScR) Checklist**

| SECTION | ITEM | PRISMA-ScR CHECKLIST ITEM | REPORTED ON PAGE # |
| --- | --- | --- | --- |
| TITLE | | | |
| Title | 1 | Identify the report as a scoping review. | 1 |
| ABSTRACT | | | |
| Structured summary | 2 | Provide a structured summary that includes (as applicable): background, objectives, eligibility criteria, sources of evidence, charting methods, results, and conclusions that relate to the review questions and objectives. | 2 |
| INTRODUCTION | | | |
| Rationale | 3 | Describe the rationale for the review in the context of what is already known. Explain why the review questions/objectives lend themselves to a scoping review approach. | 3 |
| Objectives | 4 | Provide an explicit statement of the questions and objectives being addressed with reference to their key elements (e.g., population or participants, concepts, and context) or other relevant key elements used to conceptualize the review questions and/or objectives. | 3 |
| METHODS | | | |
| Protocol and registration | 5 | Indicate whether a review protocol exists; state if and where it can be accessed (e.g., a Web address); and if available, provide registration information, including the registration number. | 4 |
| Eligibility criteria | 6 | Specify characteristics of the sources of evidence used as eligibility criteria (e.g., years considered, language, and publication status), and provide a rationale. | 4 |
| Information sources* | 7 | Describe all information sources in the search (e.g., databases with dates of coverage and contact with authors to identify additional sources), as well as the date the most recent search was executed. | 5 |
| Search | 8 | Present the full electronic search strategy for at least 1 database, including any limits used, such that it could be repeated. | Appendix 1 |
| Selection of sources of evidence† | 9 | State the process for selecting sources of evidence (i.e., screening and eligibility) included in the scoping review. | 5 |
| Data charting process‡ | 10 | Describe the methods of charting data from the included sources of evidence (e.g., calibrated forms or forms that have been tested by the team before their use, and whether data charting was done independently or in duplicate) and any processes for obtaining and confirming data from investigators. | 5 |
| Data items | 11 | List and define all variables for which data were sought and any assumptions and simplifications made. | 5, Appendix 5 |
| Critical appraisal of individual sources of evidence§ | 12 | If done, provide a rationale for conducting a critical appraisal of included sources of evidence; describe the methods used and how this information was used in any data synthesis (if appropriate). | 6 |
| Synthesis of results | 13 | Describe the methods of handling and summarizing the data that were charted. | 6 |
| RESULTS | | | |
| Selection of sources of evidence | 14 | Give numbers of sources of evidence screened, assessed for eligibility, and included in the review, with reasons for exclusions at each stage, ideally using a flow diagram. | 7, Fig 1 |
| Characteristics of sources of evidence | 15 | For each source of evidence, present characteristics for which data were charted and provide the citations. | Table 1 |
| Critical appraisal within sources of evidence | 16 | If done, present data on critical appraisal of included sources of evidence (see item 12). | NA |
| Results of individual sources of evidence | 17 | For each included source of evidence, present the relevant data that were charted that relate to the review questions and objectives. | Tables 1–2; Supplementary Tables S1–S9 |
| Synthesis of results | 18 | Summarize and/or present the charting results as they relate to the review questions and objectives. | Results p 8-10; Fig 2 |
| DISCUSSION | | | |
| Summary of evidence | 19 | Summarize the main results (including an overview of concepts, themes, and types of evidence available), link to the review questions and objectives, and consider the relevance to key groups. | 11 |
| Limitations | 20 | Discuss the limitations of the scoping review process. | 12 |
| Conclusions | 21 | Provide a general interpretation of the results with respect to the review questions and objectives, as well as potential implications and/or next steps. | 12-13 |
| FUNDING | | | |
| Funding | 22 | Describe sources of funding for the included sources of evidence, as well as sources of funding for the scoping review. Describe the role of the funders of the scoping review. | 13 |

JBI = Joanna Briggs Institute; PRISMA-ScR = Preferred Reporting Items for Systematic reviews and Meta-Analyses extension for Scoping Reviews.

* Where *sources of evidence* (see second footnote) are compiled from, such as bibliographic databases, social media platforms, and Web sites.

† A more inclusive/heterogeneous term used to account for the different types of evidence or data sources (e.g., quantitative and/or qualitative research, expert opinion, and policy documents) that may be eligible in a scoping review as opposed to only studies. This is not to be confused with *information sources* (see first footnote).

‡ The frameworks by Arksey and O’Malley (6) and Levac and colleagues (7) and the JBI guidance (4, 5) refer to the process of data extraction in a scoping review as data charting*.*

§ The process of systematically examining research evidence to assess its validity, results, and relevance before using it to inform a decision. This term is used for items 12 and 19 instead of "risk of bias" (which is more applicable to systematic reviews of interventions) to include and acknowledge the various sources of evidence that may be used in a scoping review (e.g., quantitative and/or qualitative research, expert opinion, and policy document).

*From:* Tricco AC, Lillie E, Zarin W, O'Brien KK, Colquhoun H, Levac D, et al. PRISMA Extension for Scoping Reviews (PRISMAScR): Checklist and Explanation. Ann Intern Med. 2018;169:467–473. [doi: 10.7326/M18-0850](http://annals.org/aim/fullarticle/2700389/prisma-extension-scoping-reviews-prisma-scr-checklist-explanation).

**Supplementary Data S3**

**Full text exclusion reasons**

| Abramowicz 2020 | Wrong outcomes |
| --- | --- |
| Adib 2008 | Wrong outcomes |
| Aeder 2021 | Review-reference list screened |
| Agarwal 2008 | Wrong outcomes |
| Akdeniz 2020 | Relationship between pain and disease markers not explored or reported clearly |
| Alekseev 2013 | Relationship between pain and disease markers not explored or reported clearly |
| Alimanovic 2021 | Relationship between pain and disease markers not explored or reported clearly |
| Álvarez-Madrid 2009 | Wrong outcomes |
| Anink 2015 | Relationship between pain and disease markers not explored or reported clearly |
| Anonymous | Ongoing trial results not yet published |
| Anthony 2005 | Review-reference list screened |
| Anthony 2007 | Review-reference list screened |
| Arneng 2024 | Relationship between pain and disease markers not explored or reported clearly |
| Artamonov 2023 | Wrong outcomes |
| Atar 2020 | Wrong patient population |
| Avar-Aydin 2022 | Wrong patient population |
| Ayed 2023 | Wrong outcomes |
| Basbozkurt 2015 | Relationship between pain and disease markers not explored or reported clearly |
| Baydoun 2023 | Relationship between pain and disease markers not explored or reported clearly |
| Behrens 2022 | Wrong patient population |
| BenDhia 2023 | Abstract only without sufficient data |
| Berntson 2008 | Wrong outcomes |
| Berntson 2021 | Relationship between pain and disease markers not explored or reported clearly |
| Berntson 2024 | Relationship between pain and disease markers not explored or reported clearly |
| Bettaieb 2021 | Relationship between pain and disease markers not explored or reported clearly |
| Bingham 2012 | Relationship between pain and disease markers not explored or reported clearly |
| Bingham 2017 | Relationship between pain and disease markers not explored or reported clearly |
| Bohr 2015 | Relationship between pain and disease markers not explored or reported clearly |
| Boiu 2012 | Wrong outcomes |
| Bollow 1998 | Wrong patient population |
| Borzani 2015 | Wrong outcomes |
| Brandon 2025 | Relationship between pain and disease markers not explored or reported clearly |
| Breton 2011 | Wrong outcomes |
| Bromberg 2014 | Review-reference list screened |
| Brown 2012 | Relationship between pain and disease markers not explored or reported clearly |
| Brown 2020 | Wrong patient population |
| Brunelli 2018 | Relationship between pain and disease markers not explored or reported clearly |
| Brunner 2004 | Relationship between pain and disease markers not explored or reported clearly |
| Brunner 2013 | Relationship between pain and disease markers not explored or reported clearly |
| Brunner 2023 | Relationship between pain and disease markers not explored or reported clearly |
| Burgos-Vargas 2017 | Relationship between pain and disease markers not explored or reported clearly |
| Burrone 2022 | Wrong outcomes |
| Carlomagno 2017 | Relationship between pain and disease markers not explored or reported clearly |
| Carmona 2013 | Wrong patient population |
| Chaplin 2021 | Review-reference list screened |
| Chauvin 2013 | Wrong outcomes |
| Chauvin 2014 | Relationship between pain and disease markers not explored or reported clearly |
| Chauvin 2017 | Wrong patient population |
| Chew 2014 | Abstract only without sufficient data |
| Choida 2019 | Abstract only without sufficient data |
| Chou 2012 | Relationship between pain and disease markers not explored or reported clearly |
| Collin 2022 | Relationship between pain and disease markers not explored or reported clearly |
| Consolaro 2007 | Wrong outcomes |
| Consolaro 2011 | Wrong outcomes |
| Cornelissen 2014 | Wrong outcomes |
| Covone 2014 | Wrong outcomes |
| Craig 2021 | Relationship between pain and disease markers not explored or reported clearly |
| Datta 2024 | Relationship between pain and disease markers not explored or reported clearly |
| Davidsone 2011 | Wrong outcomes |
| deLalouvière 2014 | Review-reference list screened |
| Demir 2018 | Wrong outcomes |
| DeSomer 2011 | Wrong outcomes |
| Dghaies 2023 | Relationship between pain and disease markers not explored or reported clearly |
| Dghaies 2023 | Wrong outcomes |
| Diafa 2011 | Wrong outcomes |
| DiCarlo 2021 | Wrong patient population |
| Dzhus 2020 | Abstract only without sufficient data |
| Ekelund 2025 | Wrong outcomes |
| El-Azeem 2012 | Relationship between pain and disease markers not explored or reported clearly |
| Elhai 2011 | Relationship between pain and disease markers not explored or reported clearly |
| Elhai 2013 | Relationship between pain and disease markers not explored or reported clearly |
| Elhosiny 2020 | Relationship between pain and disease markers not explored or reported clearly |
| Elndari 2018 | Abstract only without sufficient data |
| Elzayat 2023 | Wrong outcomes |
| ErguluEsmen 2020 | Relationship between pain and disease markers not explored or reported clearly |
| Fellas 2022 | Relationship between pain and disease markers not explored or reported clearly |
| Fernanda 2011 | Wrong outcomes |
| FerrazJúnior 2012 | Wrong outcomes |
| Filippou 2011 | Wrong patient population |
| Fischer 2021 | Wrong outcomes |
| Fisher 2014 | Wrong outcomes |
| Fox 2016 | Wrong outcomes |
| Garcia-Munitis 2006 | Wrong outcomes |
| Garip 2011 | Wrong patient population |
| Gattinara 2013 | Abstract only without sufficient data |
| Giancane 2017 | Review-reference list screened |
| Gmuca 2015 | Review-reference list screened |
| Gragg 1996 | Wrong patient population |
| Guzman 2015 | Abstract only without sufficient data |
| Hanns 2017 | Relationship between pain and disease markers not explored or reported clearly |
| Hanns 2018 | Relationship between pain and disease markers not explored or reported clearly |
| Hanns 2018 | Wrong outcomes |
| Hanns 2020 | Relationship between pain and disease markers not explored or reported clearly |
| Hari 2014 | [Wrong outcomes](https://ard.bmj.com/content/73/Suppl_2/1093.1) |
| Hashaad 2017 | Wrong outcomes |
| Hendry 2012 | Wrong outcomes |
| Hernandez-Baldizon 2017 | Relationship between pain and disease markers not explored or reported clearly |
| Heshin-Bekenstein 2019 | Wrong outcomes |
| Hoeksma 2013 | Wrong outcomes |
| Hua 2020 | Wrong patient population |
| Huang 2012 | Relationship between pain and disease markers not explored or reported clearly |
| Ibrahim 2022 | Wrong outcomes |
| Iversen 2014 | Abstract only without sufficient data |
| Jadon 2013 | Review-reference list screened |
| James 2022 | Relationship between pain and disease markers not explored or reported clearly |
| Janow 2016 | Relationship between pain and disease markers not explored or reported clearly |
| Januskeviciute 2017 | Wrong outcomes |
| Jarvis 2006 | Review-reference list screened |
| Johansson 2024 | Relationship between pain and disease markers not explored or reported clearly |
| Kharrat 2022 | Wrong outcomes |
| Kithan 2018 | Wrong outcomes |
| Kolkhidova 2023 | Wrong outcomes |
| Konca 2012 | Wrong patient population |
| Koos 2014 | Wrong outcomes |
| Kristensen 2016 | Review-reference list screened |
| Kuis 1997 | Review-reference list screened |
| Lanni 2021 | Wrong outcomes |
| Larheim 1982 | Relationship between pain and disease markers not explored or reported clearly |
| Larheim 2015 | Review-reference list screened |
| LassouedFerjani 2023 | Wrong outcomes |
| LassouedFerjani 2024 | Wrong outcomes |
| Leegaard 2013 | Wrong outcomes |
| Liu 2025 | Wrong patient population |
| Mahler 2017 | Relationship between pain and disease markers not explored or reported clearly |
| Mahmoud 2022 | Relationship between pain and disease markers not explored or reported clearly |
| Maritsi 2013 | Relationship between pain and disease markers not explored or reported clearly |
| Marques 2013 | Relationship between pain and disease markers not explored or reported clearly |
| MartiMasanet 2021 | Wrong outcomes |
| Maschio 2019 | Abstract only without sufficient data |
| May 2011 | Wrong outcomes |
| Mazzoni 2018 | Wrong outcomes |
| Mazzoni 2023 | Relationship between pain and disease markers not explored or reported clearly |
| McErlane 2012 | Wrong outcomes |
| Miotto 2013 | Wrong outcomes |
| Molina 2014 | Wrong outcomes |
| Moued 2013 | Relationship between pain and disease markers not explored or reported clearly |
| Murias 2018 | Abstract only without sufficient data |
| Murray 1996 | Wrong outcomes |
| Nishat 2024 | Wrong outcomes |
| Nordal 2011 | Wrong outcomes |
| Nordal 2017 | Relationship between pain and disease markers not explored or reported clearly |
| Nørgaard 2016 | Wrong outcomes |
| Nusman 2014 | Abstract only without sufficient data |
| Nykvist 2017 | Wrong patient population |
| Oen 2003 | Relationship between pain and disease markers not explored or reported clearly |
| Oen 2005 | Wrong outcomes |
| Oliveira 2017 | Wrong patient population |
| Oliver 2022 | Relationship between pain and disease markers not explored or reported clearly |
| Olson 1991 | Relationship between pain and disease markers not explored or reported clearly |
| Ören 1996 | Abstract only without sufficient data |
| Ozen 1997 | Wrong outcomes |
| Ożga 2024 | Wrong patient population |
| Paivi 2011 | Relationship between pain and disease markers not explored or reported clearly |
| Pascoli 2010 | Wrong outcomes |
| Pelajo 2011 | Wrong outcomes |
| Perez 2014 | Abstract only without sufficient data |
| Phatak 2017 | Relationship between pain and disease markers not explored or reported clearly |
| Polizzi 2021 | Relationship between pain and disease markers not explored or reported clearly |
| Quesada-Masachs 2017 | Abstract only without sufficient data |
| Reilly 1988 | Wrong patient population |
| Ridella 2019 | Relationship between pain and disease markers not explored or reported clearly |
| Ridella 2023 | Wrong outcomes |
| Ringold 2013 | Wrong outcomes |
| Robin 2012 | Wrong outcomes |
| Rongo 2019 | Relationship between pain and disease markers not explored or reported clearly |
| Ronsivalle 2024 | Review-reference list screened |
| Rosenbaum 2025 | Review-reference list screened |
| Sabounji 2024 | Wrong outcomes |
| Sadreddini 2009 | Wrong patient population |
| Salamzade 2025 | Wrong outcomes |
| Schoth 2022 | Review-reference list screened |
| Shanmugavel 2008 | Relationship between pain and disease markers not explored or reported clearly |
| Shelmerdine 2018 | Review-reference list screened |
| Shiff 2020 | Relationship between pain and disease markers not explored or reported clearly |
| Shoop-Worrall 2024 | Relationship between pain and disease markers not explored or reported clearly |
| Shyen 2013 | Wrong outcomes |
| Shyen 2014 | Wrong outcomes |
| Sivas 2009 | Wrong patient population |
| Smith 2021 | Review-reference list screened |
| Solari 2008 | Relationship between pain and disease markers not explored or reported clearly |
| Somers 2010 | Wrong patient population |
| Sontichai 2018 | Wrong outcomes |
| Stead 2023 | Wrong outcomes |
| Stinson 2012 | Review-reference list screened |
| Stoustrup 2021 | Review-reference list screened |
| Susarla 2012 | Abstract only without sufficient data |
| Tadrous 2015 | Relationship between pain and disease markers not explored or reported clearly |
| Tarakci 2019 | Relationship between pain and disease markers not explored or reported clearly |
| Tarkiainen 2019 | Relationship between pain and disease markers not explored or reported clearly |
| Terreri 2011 | Wrong outcomes |
| Tesher 2022 | Relationship between pain and disease markers not explored or reported clearly |
| Thastum 2001 | Wrong outcomes |
| Tibaldi 2019 | Relationship between pain and disease markers not explored or reported clearly |
| Toiviainen-Salo 2012 | Wrong outcomes |
| Trincianti 2020 | Wrong outcomes |
| Trouvin 2022 | Wrong patient population |
| Tucker 2022 | Relationship between pain and disease markers not explored or reported clearly |
| Tufekci 2023 | Relationship between pain and disease markers not explored or reported clearly |
| Tuomi 2024 | Wrong outcomes |
| Tzaribachev 2012 | Wrong outcomes |
| Urtane 2018 | Wrong outcomes |
| Vallogini 2023 | Wrong outcomes |
| VanDerHeijden 2021 | Wrong outcomes |
| VanderLinden 1988 | Wrong outcomes |
| Vanoni 2016 | Relationship between pain and disease markers not explored or reported clearly |
| Varni 1988 | Relationship between pain and disease markers not explored or reported clearly |
| VegaFernandez 2023 | Wrong outcomes |
| Ventura-Ríos 2018 | Wrong outcomes |
| Vidal 2017 | Abstract only without sufficient data |
| Vidovic 2013 | Wrong outcomes |
| Vijayan 2012 | Relationship between pain and disease markers not explored or reported clearly |
| Wabi 2013 | [Abstract only without sufficient data](https://ard.eular.org/article/S0003-4967(24)47574-2/abstract) |
| Wadeley 2018 | Wrong patient population |
| Ward 2016 | Wrong outcomes |
| Weiss 2008 | Relationship between pain and disease markers not explored or reported clearly |
| Weiss 2014 | Wrong outcomes |
| Weiss 2014 | Review-reference list screened |
| Weitzman 2020 | Wrong outcomes |
| Yamada 2016 | Wrong patient population |
| Yildiz-Kabak 2024 | Relationship between pain and disease markers not explored or reported clearly |
| Zweers 2021 | Review-reference list screened |

**Supplementary Data S4**

Data extraction template

2. Study ID
3. Title
4. Source type
5. Journal
6. Country in which the study was conducted
7. Study aim / Research question
8. Study design
9. Start / End date
10. Sample size
11. Age range
12. Age (Median or Mean)
13. Age at onset / diagnosis
14. Disease duration
15. JIA subtype(s)
16. JIA subtype distribution
17. Gender distribution (% female)
18. Race / ethnicity (if reported)
19. Disease status
20. Treatment / medication
21. Comorbidities
22. Pain outcome(s) reported

-Construct ie intensity/interference

-Measure ie VAS/NRS/QST

-Recall period ie prev week (average)/current or present/not reported

-Who reported? (CYP or parent/proxy)

23. Blood / fluid biomarkers

-Other bloods

-Analysed in relation to pain?

24. Musculoskeletal imaging

-Analysed in relation to pain?

25. Physician / composite assessments of disease activity

-Analysed in relation to pain outcomes?

26. Was a composite disease activity score used that includes subjective components (e.g. pain or well-being)?

-Analysed in relation to pain outcomes?

27. Anatomical location
28. State the specific objective–pain comparison(s) analysed ie Pain VAS vs ESR or QST vs JADAS
29. List any objective measures reported but *not* examined in relation to pain outcomes
30. Method of exploration
31. Time-point
32. Results: Pain
33. Results: Objective measures
34. Relationship between pain and measures of disease activity
35. Authors’ explanation (if discordance or weak/no association between pain and disease activity or other objective markers was found)

**Table S1.** Participant characteristics

| First Author/ Year | Age (Median or Mean) years | Sex distribution (% F) | | Race/ethnicity | | JIA subtype distribution (%) | | Disease status | |  |
| --- | --- | --- | --- | --- | --- | --- | --- | --- | --- | --- |
| Abu-Saad 1995^1^ | Not reported (range 7-16) | 73 | | 31/33 Dutch origin | | Poly JRA 45, sJIA 30 , Pauci JRA 25 | | Mixed | |  |
| Algergawy 2011^2^ | Mean 9 | 55 | | Not reported | | Poly 40 , Pauci JRA 35, sJIA 25 | | Mixed | |  |
| Alongi 2022^3^ | Mean 11 PhGA=0 group)  Mean 12 (PhGA>0 group) | PhGA=0: 35 PhGA>0: 33 | | Not reported | | PhGA=0 group:  Oligo 48, RF– poly 21, sJIA 12, ERA 89, Undiff 5, PsA 3, RF+ poly 2  PhGA>0 group:  Oligo 36, RF– poly 23, ERA 14, sJIA 13, Undiff 6, RF+ poly 5, PsA 4 | | Inactive/remission | |  |
| Alqanatish 2011^4^ | Mean 10 | 40 | | Not reported | | Oligo-p 80, Oligo-e 20 | | Mixed | |  |
| Armbrust 2013^5^ | Median 12 | 67 | | Not reported | | Poly 49, Oligo 38, ERA 4 ,sJIA 8, Other 12 | | Mixed | |  |
| Arnstad 2019^6^ | Median Baseline 6 FU 15 | 70 | | Not reported | | Oligo 49 | | Mixed | |  |
| Arnstad 2020^7^ | Median 23 | 71 | | Mostly Caucasian | | Oligo-p 28, RF– poly 24, Oligo-e 19, Undiff 15, ERA 7, PsA 4, RF+ poly 2, sJIA 1 | | Mixed | |  |
| Bandeira 2006^8^ | Not reported | 77 | | Not reported | | Oligo-p 33 , Oligo-e 31, Poly 19 , sJIA 15, PsA 3 | | Not reported | |  |
| Biancardi 2015^9^ | Not reported | Not reported | | Not reported | | Oligo-p 61, Oligo-e 14, RF– poly 21, sJIA 4 | | Mixed | |  |
| Bloom 1995^10^ | Not reported | 67 | | Not reported | | sJIA 83, Pauci JRA 17 | | Active | |  |
| Bou Antoun 2017^11^ | Not reported | 60 | | Not reported | | All ERA | | Not reported | |  |
| Brandon 2017^12^ | Median 12 | 70 | | White 85%, Black/African, American 7%, Asian 1%, Other 6% More than 1 race 1% | | Oligo 32, ERA 22, RF− poly 22, Undiff 10 PsA 9, sJIA 4.5, RF+ poly 2 | | Mixed | |  |
| Brandon 2021^13^ | Median 14 | 37 | | White 71 % | | ERA 89 | | Mixed | |  |
| Carlsson 2023^14^ | Median 12 | 73 | | Not reported | | Oligo 43, Poly 33, other subtypes 24 | | Mixed | |  |
| Chaplin 2018^15^ | Mean 20 | 68 | | White British 66% | | Poly 40 | | Not reported | |  |
| Collin 2024^16^ | Mean 11 | 72 | | Not reported | | Oligo 46, RF– poly 33, PsA 9, sJIA 6, RF + poly 1, ERA 1, Undiff 1 | | Active | |  |
| Coulton 1987^17^ | Mean 9 | 70 | | White 97% Black 3% | | Poly JRA 56, Pauci JRA 44 Children with sJIA classified as Poly or Pauci | | Mixed | |  |
| de Oliveira Sato 2011^18^ | Median 12 | 56 | | Not reported | | Oligo-p 89, Oligo-e 11 | | Mixed | |  |
| de Vos 2023^19^ | Not reported | Not reported | | Not reported | | Not reported | | Mixed | |  |
| Dekker 2010^20^ | Mean | 88 | | Not reported | | RF− poly 68, RF+ poly 9, Oligo-e 9, Oligo-p 6, ERA 6, PsA 3 | | Mixed | |  |
| Eboli 2022^21^ | Not reported | Not reported | | Not reported | | Not reported | | Mixed | |  |
| Elfving 2023^22^ | Median 10 | 67 | | Not reported | | Oligo-p 49, ERA 18, RF– poly 14, PsA 8, Oligo-e 6, RF+ poly 6 | | Mixed | |  |
| Elliott 1997^23^ | 16 | 100 | | Asian | | sJIA | | Active | |  |
| Esbersen 2018^24^ | Not reported | Not reported | | Not reported | | Not reported | | Not reported | |  |
| Flatø 2002^25^ | Mean  Baseline 8 FU 23 | Total cohort: 67 Those with sacroiliitis: 30 | | Not reported | | Pauci JRA 64, Poly 28, sJIA | | Not reported | |  |
| Ferrazzo 2019^26^ | Not clear | F (single case) | | Not reported | | NA | | Not reported | |  |
| Gestler 2022^27^ | Not reported | Not reported | | Not reported | | Not reported | | Inactive/remission | |  |
| Gorczyca 2017^28^ | Not reported | Not reported | | Not reported | | Not reported | | Mixed | |  |
| Guzman 2017^29^ | Median 8 | 68 | | Not reported | | Oligo 37, RF− poly 21, ERA 13, Undiff 11, Systemic 8, PsA 6, RF+ poly 4 | | Newly diagnosed (inception cohort) | |  |
| Hara 2017^30^ | Not reported | 100 | | Japanese | | Poly 66 ,Oligo (monoarticular) 33 | | Not reported | |  |
| Hogewe 1995^31^ | Mean 12 | 68 | | Not reported | | Oligo 56, Poly 23, sJIA 21 | | Mixed | |  |
| Hospach 2014^32^ | Median 8 | 54 | | Not reported | | Oligo-e 46, RF– poly 31, PsA 15, sJIA 8 | | Mixed | |  |
| Hu 2019^33^ | Mean 9 | 31 | | Not reported | | All sJIA | | Mixed | |  |
| Ilowite 1992^34^ | Mean 10 | 83 | | Not reported | | Pauci JRA 61, Poly 28, sJIA 11 | | Not reported | |  |
| Katsicas 2017^35^ | Median 12 | 10 | | Not reported | | All ERA | | Mixed | |  |
| Katsicas 2018^36^ | Median 12 | 17 | | Not reported | | All ERA | | Not reported | |  |
| Khanom 2022^37^ | Mean 11 | 68 | | Not reported | | RF− poly 29, Oligo-p 25, ERA 9, Oligo-e 8, RF+ poly 6, Undiff 6, sJIA 4, PsA 2 | | Mixed | |  |
| Kovalchuk 2018^38^ | Mean 13 | 48 | | Not reported | | Poly 49, Oligo-p 31.7, Oligo-e 20 | | Mixed | |  |
| Küseler 2005^39^ | Mean 12 | Not reported | | Not reported | | Oligo 60, Poly 27, sJIA 3 | | Mixed | |  |
| Learoyd 2019^40^ | Mean16 | 57 | | Not reported | | Poly 56, ERA 33, Oligo 6, sJIA 5 | | Mixed | |  |
| Leksell 2012^41^ | Mean 14 | 71 | | Not reported | | Oligo 21, Poly 36, sJIA 12, PsA 17, other 14 | | Not reported | |  |
| Lomholt 2013^42^ | Mean 13 | | Standard treatment group: 82 Anti-TNF group: 71 | | Not reported | | Standard treatment group (n=50): RF– poly 44 ,Oligo-p 22, Oligo-e 18, sJIA 10, PsA 2  Anti-TNF group (n=41):  RF– poly 42, Oligo-e 22, sJIA 22, PsA 5 ERA 5, Oligo-p 2, RF+ poly 2 | | Mixed | |
| Magni-Manzoni 2005^43^ | Not reported | 79 | | Not reported | | Oligo 68 (-p 49 , -e 19), Poly 13, sJIA 10 , ERA/PsA 9 | | Flare | |  |
| Magni-Manzoni 2009^44^ | Not reported | 84 | | Not reported | | Oligo-e 38, Oligo-p 25, RF– poly 22, ERA 13, PsA 3 | | Mixed | |  |
| Mahmood 2014^45^ | Not reported | Not reported | | Not reported | | Not reported | | Not reported | |  |
| Malattia 2010^46^ | Mean 12 | Wrist group: 83 Hip group: 40 | | Not reported | | Wrist group: Oligo-e 42, sJIA 25, RF-neg poly 8, RF-pos poly 8, Oligo-p 8, Undif 8  Hip group: sJIA 60,Oligo-e 10, Oligo-p 10, ERA 10,Undiff 10 | | Active | |  |
| Malleson 2004^47^ | Not reported, participants split into two age groups 8-15 years (n = 183) >16 years (n = 205) | 8-15 group: 74 >16 group: 81 | | Age Group: 8-15 years White 85%, Aboriginal or part Aboriginal 10%, Other (East Indian, Asian, Black, other) 5%  Age Group: >16 years White 82%, Aboriginal or part Aboriginal 15%, Other (East Indian, Asian, Black, other) 4% | | Age Group: 8-15 years Pauci JRA 67, RF– poly 18, sJIA 13, RF+ poly 3  Age Group: >16 years Pauci JRA 48, RF– poly 23, RF+ poly 17, sJIA 12 | | Mixed | |  |
| Mannion 2019^48^ | Median 12 | 73 | | White 83%, Hispanic 9%, Black 5%, Asian 4%, Other 1% | | RF - poly 39, Oligo 26, sJIA 10, ERA 9, RF + poly 8, PsA 7, Undiff 2 | | Mixed | |  |
| Mannion 2022^49^ | Not reported | Not reported | | Not reported | | Oligo; Poly (distrib not reported) | | Mixed | |  |
| McGrath 2023^50^ | Not reported | Persisting pain 77 Decreasing controls 64 | | Not reported | | Persist vs control RF+ Poly 13 vs 4 RF- Poly 20 vs 23  Undiff 23 vs 9 Oligo: 10 vs 31  ERA 23 vs 20 | | Mixed | |  |
| McLellan 2023^51^ | Not reported | 79 | | Not reported | | Oligo 46, RF– poly 21, ERA 11, sJIA 7, RF+ poly 7, PsA 5, IBD related 4 | | Mixed | |  |
| Melchiorre 2010^52^ | Median 11 | 84 | | Not reported | | After 6 months, Oligo-e 16 | | Active | |  |
| Miller 2011^53^ | Median  sJIA 11 Oligo-p 10 Oligo-e 15 RF- poly 11 RF+ poly 14 PsA 13 | sJIA 44 Oligo-p 83  Oligo-e 83  RF- poly 85  RF+ poly 100  PsA 67  ERA 50 | | White/non-Hispanic 78%, Hispanic 13%,  African American 3% , Asian 3%, Other 4% | | Oligo-p 40, RF- poly 25, sJIA 11, ERA 11,  RF+ poly 8, Oligo-e 4, PsA 2 | | Not reported | |  |
| Min 2020^54^ | Mean 12 | 67 | | Not reported | | Poly 64, Oligo 36 | | Mixed | |  |
| Minden 2014^55^ | Mean 23 at last follow up | Not reported | | Not reported | | Not reported | | Mixed | |  |
| Miyamae 2019^56^ | Not reported | 67 | | Not reported | | All RF– poly | | Active | |  |
| Mohammed 2012^57^ | Mean 14 | 65 | | Not reported | | Poly 68, Oligo 18, sJIA 15 | | Mixed | |  |
| Mosa 2021^58^ | 5 | F | | Not reported | | All sJIA | | Active | |  |
| Mountain 2025^59^ | Median Whole cohort 15   Low pain/low disease 13 Low pain/high disease 15  High pain/low disease 13 High pain/high disease 15 | Whole cohort (n=363) 76 Analytical subset (n = 187) 52 Low pain/low disease (n = 59) 56 Low pain/high disease (n = 44) 55 High pain/low disease (n = 14) 71 High pain/high disease (n = 70) 44 | | White%  Whole cohort 89.5%  Low pain/low disease (n = 59) 90%  Low pain/high disease (n = 44) 80%  High pain/low disease (n = 14) 93%  High pain/high disease (n = 70) 81% | | Oligo-p 34, RF− poly 28, PsA 8, Oligo-e 7, sJIA 6, Undiff 6, RF+ poly 5 | | Mixed | |  |
| Naddei 2023^60^ | Not reported | Not reported | | Not reported | | Not reported | | Inactive/remission | |  |
| Nguyen 2024^61^ | Median ReACCh-Out 10 CAPRI 9 | 65/61 | | Not reported | | ReACCh-Out/CAPRI Oligo 41/45, RF– poly 20/18, ERA 13/16, Undiff 10/7, PsA 6/6, sJIA 6/4 RF+ poly 4/4 | | Mixed | |  |
| Nistala 2007^62^ | Mean 14 | Not reported | | Not reported | | ERA 35,Poly 27,sJIA 21,Oligo 12, PsA 6 | | Mixed | |  |
| Oen 2009^63^ | Median 9 | 66 | | Not reported | | Oligo 41, RF– poly 20, Undiff 12, ERA 10, sJIA 7,PsA 7, RF+ poly 4 | | Mixed | |  |
| Oen 2018^64^ | Not reported | 64 | | Any European 85%, Any, Canadian Aboriginal 9%, Any other (Asian, Latin American, Black, or Arab) 19 % | | Oligo-p 34, RF - poly 20, ERA 14, Undiff 10, Oligo-e 6, sJIA 6, PsA 6, RF+ poly 4 | | Mixed | |  |
| Oen 2021^65^ | Median 10 | 60-66 across stages | | Not reported | | At diagnosis: Oligo 41, Poly RF- 20, ERA 15, PsA 6, sJIA 5, Poly RF+ 3, Undiff 10 | | Mixed | |  |
| Palmisani 2006^66^ | Median Early 4 Advanced 11 Longstanding 16 | Early 74  Advanced 79 Longstanding 74 | | Not reported | | Early: Oligo-p 57, Oligo-e 23, Poly 13, sJIA 7  Advanced:  Oligo-p 32, Oligo-e 32, Poly 23, sJIA 10, PsA 4 Longstanding:  Oligo-p 33, Oligo-e 26, Poly 18, sJIA 20, PsA 3 | | Mixed | |  |
| Papsdorf 2011^67^ | Mean 13 | 65 | | Not reported | | RF– poly 29, Oligo-e 16, ERA 14, sJIA 13, RF+ poly 10, PsA 8, Oligo-p 5, Non-classifiable 4 | | Active | |  |
| Pedersen 2008^68^ | Not reported | Not reported | | Not reported | | Not reported | | Not reported | |  |
| Pinto 2018^69^ | 12 | 0 (male patient) | | Caucasian | | NA | | Not reported | |  |
| Portelli 2014^70^ | Median 10 | 80 | | Not reported | | Oligo 43, Poly 40,sJIA 13, PsA 2, ERA 2 | | Not reported | |  |
| Quilis 2024^71^ | Median 11 | 63 | | Not reported | | Oligo-p 45, RF– poly 19, PsA 8, sJIA 7, Oligo-e 7, ERA 7, Undiff 5, RF+ poly 3 | | Mixed | |  |
| Radziszewska 2019^72^ | Not reported | Not reported | | Not reported | | All ERA | | Not reported | |  |
| Randell 2024^73^ | Mean 14 | 70 | | Race  White 81%, Black 3%, Asian 2%, > 1 race 4%, Other 3% Prefer not to answer: 8%  Ethnicity Non-Hispanic 92 %, Hispanic 8% | | Poly 37, Oligo 34, Spondyloarthritis (ERA, PsA, Juvenile AS) 25, sJI 5 | | Mixed | |  |
| Rapoff 1991^74^ | Mean 9 | 87 | | Not reported | | Poly 61, Pauci JRA 36, sJIA 3 | | Not reported | |  |
| Rashid 2018^75^ | Median 8 | 66 | | Not reported | | Oligo-p 43, RF– poly 26, PsA 8, sJIA 6, ERA 5, Oligo-e 5, RF+ poly 4, Undiff 3 | | Mixed | |  |
| Ravelli 1997^76^ | Mean 11 | 56 | | Not reported | | sJIA 36, Pauci JRA 35, Poly 29 | | Active | |  |
| Ross 1989^77^ | Not reported | 76 | | Not reported | | Pauci JRA 44, RF- poly 35, sJIA onset with Poly involvement 11, sJIA 9, RF + poly 2 | | Mixed | |  |
| Rumsey 2020^78^ | Not reported | Children with enthesitis: 43 Children without enthesitis: 69 | | Not reported | | Children with enthesitis (n=214): ERA 64, Undiff 18, RF- poly 8, Oligo 6, PsA 2, RF+ poly 2  In children without enthesitis (n=1157): Oligo 47, RF- poly 22, Undiff 8, sJIA 7, PsA 7, ERA 5, RF+ poly 4 | | Mixed | |  |
| Rypdal 2024^79^ | Median Baseline 6  FU 23 | 68 | | Not reported | | At 18 year follow up: Oligo-p 27, RF- poly 18, Undiff 15, ERA 10, PsA 7 Oligo-e 20 (reported at baseline, significant decrease in overall Oligo group due to shift to Oligo extended category over time) sJIA 3, RF+ poly 1 | | Mixed | |  |
| Schanberg 1997^80^ | Mean 12 | 64 | | Caucasian 95% African-American 5% | | Poly 41, ERA 21, sJIA 18, Oligo 14, PsA 5 | | Mixed | |  |
| Schanberg 2003^81^ | Mean 12 | 59 | | White 85% African American 15% | | Poly JRA 59, sJIA 27, PsA 7, JSpA 7 | | Mixed | |  |
| Scott 1977^82^ | Not reported | Not reported | | Not reported | | Not reported | | Not reported | |  |
| Sgarlat 2011^83^ | Mean 12 | 73 | | Not reported | | Not reported | | Mixed | |  |
| Sherry 1990^84^ | Not reported | 73 | | Not reported | | Whole cohort (n=293): Pauci 47, Poly 36, sJIA 17  Painless cohort (n=41) Pauci 88, Poly 7, sJIA 5 | | Active | |  |
| Shiff 2018^85^ | Mean 9 | 64 | | any part Caucasian 84% | | Oligo 40, RF- poly 20.7, ERA 13.5, Undiff 9.9, sJIA 6 PsA 5.7, RF+ poly 3.8 | | Mixed | |  |
| Shoop-Worrall 2018^86^ | Median 7 | 70 | | White or Caucasian 90% | | Oligo 68, RF− poly 28, RF+ poly 5 | | Mixed | |  |
| Shoop-Worrall 2021^87^ | Median 7 | 65 | | White 90% | | Oligo-p 45, RF− poly 23, PsA 7, sJIA 6, ERA 6, Oligo-e 6, Undiff 4, RF+ poly 4 | | Mixed | |  |
| Spadaro 1996^88^ | Mean 14 | 83 | | Not reported | | Poly 62.5, Pauci 37.5 | | Active | |  |
| Spekking 2023^89^ | Median 9 | 59 to 74 across arms | | Not reported | | RF-neg Poly 77.4 to 82.8 across arms Oligo 9.4 to 16.1 across arms PsA 6.4 to 12.5 across arms | |  | |  |
| Stoll 2010^90^ | Not reported | 29 | | Not reported | | All ERA/JSpA | | Not reported | |  |
| Stoustrup 2016^91^ | Not reported | Not reported | | Not reported | | Not reported | | Not reported | |  |
| Sudhakar 2022^92^ | Not reported | 20 | | Not reported | | sJIA 55 , ERA 30, Undiff15 | | Mixed | |  |
| Svensson 2000^93^ | Mean/Median 12 | 60 | | Not reported | | Pauci JRA 74 , Poly 18, sJIA 8 | | Mixed | |  |
| Svensson 2001^94^ | Mean 3 | 92 | | Not reported | | Poly 67, Pauci 33 | | Not reported | |  |
| Tarakci 2011^95^ | Mean 12 | 64 | | Not reported | | Poly 51.9, Oligo 28.8, ERA 7.7, PsA 5.8 ,sJIA 3.8 Other 1.9 | | Mixed | |  |
| Tarkiainen 2018^96^ | Not reported | Not reported | | Not reported | | All Poly | | New-onset, followed until inactive disease (ID) on medication for at least 6 months | |  |
| Thastum 2011^97^ | Mean 11 | 83 | | Not reported | | RF– poly 40.4, Oligo-p 25.5, Oligo-e 19.1, sJIA 12.8 PsA 2.1 | | Mixed | |  |
| Thompson 1987^98^ | Mean 10 | 78 | | Caucasian 57%, Hispanic 22%, Black 17%, Asian 4% | | Pauci JRA 22, Poly 48, sJIA 26 | | Mixed | |  |
| Tirelli 2020^99^ | Median 15 | 36 | | Non-white 15% Hispanic 5% | | All ERA | | Mixed | |  |
| Trachtman 2021^100^ | Median 13 | 73 | | White 84% Black or African American 5%  Asian 6%  Other 5%  Overall 89% non-Hispanic | | JSpA 45, Poly 23, PsA 23, Oligo 9 | | Mixed | |  |
| Trachtman 2021^101^ | Mean 11 | 71 | | Race: Caucasian 94% Non-Caucasian 7% Ethnicity: Hispanic or Latino 11% Not Hispanic or Latino 89% | | Oligo-e 15, Poly RF+ 11.7, Poly RF- 73.3 | | Mixed | |  |
| Tripalo Batoš 2020^102^ | Median 14 | 81 | | Not reported | | Non-diff JSpA 67, Diff JSpA 33 | | Active | |  |
| Tucker 2009^103^ | Not reported | 65 | | Not reported | | Not reported | | Not reported | |  |
| Tupper 2013^104^ | Mean 13 | 73 | | Not reported | | Poly RF- 38, Oligo 22, sJIA 14, ERA 9, PsA 7, Poly RF+ 5, Undiff 5 | | Not reported | |  |
| Ünal 2018^105^ | Mean 13 | 56 | | Not reported | | Not reported | | Mixed | |  |
| Upadhyay 2021^106^ | Mean 13 | 69 | | Not reported | | Poly 81, PsA 6.5, Oligo 12.5 | | Mixed | |  |
| Van Der Heijden 2022^107^ | Mean 13 | 67 | | Not reported | | Not reported | | Mixed | |  |
| van Dijkhuizen 2023^108^ | Median Parent sample 11 Patient sample 13 | Parent sample 67 Patient sample 66 | | Not reported | | Parents (n = 8,643):  Oligo-p 31.8, RF– poly 23.5, Oligo-e 10.8, sJIA 10.7, ERA 10.2, RF+ poly 4.1, Undiff 5.6, PsA 3.3   Patients (n = 6,060):  Oligo-e 26, RF-pos Poly 24.3, sJIA 13.4 , Oligo-p 11.8, ERA 10.3, PsA 5.4, Undiff 5.1, RF– poly 3.6 | | Mixed | |  |
| Vandvik 1990^109^ | Median 12 | 67 | | Not reported | | Pauci JRA 31.6, Poly 31.6, JAS 19.3, Other 17.5 | | Not reported | |  |
| Varni 1987^110^ | Mean 10 | 76 | | Caucasian 57%, Hispanic 22%, Black 17%, Asian 4% | | Poly 48, sJIA 26 , Pauci JRA 22 | | Mixed | |  |
| Vega-Fernandez 2023^111^ | Median 14 | 70 | | Not reported | | All Poly | | Active | |  |
| Verstappen 2015^112^ | Not reported | 63 | | White 89% Asian, Black, or Other 7%, Mixed 7% | | Oligo-p 51.1, RF– poly 17.2, sJIA 5.7, Oligo-e 2.1, RF+ poly 3.4, PsA 5.2 , Undiff 5.9, Other 4 | | Mixed | |  |
| Viswanath 2011^113^ | Mean 17 | 9.3 | | Not reported | | All ERA | | Mixed | |  |
| von Schuckmann 2020^114^ | Not reported | 52 | | Not reported | | Oligo 46.6, ERA 38.1, PsA 8.8 | | Not reported | |  |
| Vuorimaa 2008^115^ | Mean 12 | 73 | | Not reported | | Poly 50.3, Oligo 32.4, Oligo-e 17.2 | | Not reported | |  |
| Weiss 2012^116^ | Not reported | 73 | | Not reported | | RF– poly 30, Oligo-p 27, ERA 10, RF + 7, sJIA 9, Oligo-e 8, Undiff 2, PsA 6 | | Mixed | |  |
| Weiss 2016^117^ | Mean 14 | 50 | | Not reported | | ERA 90 , PsA 10 | | Active | |  |
| Xu 2016^118^ | Mean  Active 8 Remission 6 | Active 37 Remission 47 | | Not reported | | All sJIA | | Mixed | |  |
| Yamazaki 2022^119^ | Median 14 | 29 | | Not reported | | All JSpA | | Active | |  |
| Yilmaz 2010^120^ | Mean 12 | 27 | | Not reported | | All JAS | | Active | |  |
| Zhang 2025^121^ | Mean 9 | 43 | | Han 85%, Tujia 5%, Miao 2%, Gelao 1% Yi 1%, Others (Zhuang, Tibetan, Bouyei, Li, Dong, Zang) 4% Unspecified 2% | | All sJIA | | Mixed | |  |
| Zwir 2012^122^ | Mean 12 | Not reported | | Not reported | | Oligo 52 | | Mixed | |  |

*ERA = enthesitis-related arthritis FU = follow-up ID = inactive disease JAS = juvenile ankylosing spondylitis JIA = juvenile idiopathic arthritis JRA = juvenile rheumatoid arthritis JSpA = juvenile spondyloarthritis Oligo = oligoarticular juvenile idiopathic arthritis Oligo-e = extended oligoarticular juvenile idiopathic arthritis Oligo-p = persistent oligoarticular juvenile idiopathic arthritis PhGA = physician global assessment Poly = polyarticular juvenile idiopathic arthritis PsA = psoriatic arthritis RF = rheumatoid factor RF+ = rheumatoid factor positive RF– = rheumatoid factor negative sJIA = systemic juvenile idiopathic arthritis Undiff = undifferentiated juvenile idiopathic arthritis*

**Table S2.** Study-level summary of pain and objective/clinical marker relationships

| First author/ Year | Marker category* | Pain-marker analysis | Relationship and summary of author findings** | Concordance category |
| --- | --- | --- | --- | --- |
| Abu-Saad 1995 | PhGA | Pain VAS (worst prev week) vs Physician global disease index Pain VAS present vs Physician global disease index | Concordant: Worst pain for the previous week correlated significantly with the physician's disease activity rating.  No relationship/association:  No clear linear relationship was found between the child's present pain ratings and physician-rated disease activity; strangely, children rated with mild disease activity reported the highest present pain  Present pain may underestimate the impact of arthritis because pain is often more severe during movement; worst pain over the past week may better reflect true disease activity. | **MIXED** |
| Algergawy 2011 | IMAGING | Pain VAS vs US | No relationship/association: No significant correlation between VAS scores and USG knee synovial thickness Concordant: A significant positive correlation was found between Visual Analog Scale (VAS) scores and USG knee effusion volume | **MIXED** |
| Alongi 2022 | MULTI  i.e., *pain was analysed against more than one marker within the same study* | Pain VAS vs PhGA  Pain VAS (parent reported) vs AJC | In the multivariable logistic regression, pain VAS > 0 was independently associated with having a PhGA > 0 during periods when active joint count (AJC) = 0. In the dominance analysis, pain VAS > 0 was the second strongest predictor of a non-zero PhGA Even when all other disease activity criteria were met, physicians were more likely to assign a non-zero PhGA if pain VAS was elevated.  Some children had persistent pain despite good disease control and PhGA of 0. Pain may reflect factors beyond inflammation, such as mechanical pain, structural damage, or pain amplification, especially in adolescents and those with longstanding disease. These factors were not assessed in the study. | **DISCORDANT Pain > Disease activity** |
| Alqanatish 2011 | IMAGING | Pain (narrative/clinical) vs MRI/US (Infrapatellar bursal swelling) | The bursal swelling was often painless despite clear objective evidence of inflammation via clinical examination, ultrasound, and MRI | **DISCORDANT Pain < Disease activity** |
| Armbrust 2013 | PhGA | Pain VAS vs PhGA | Concordant: Low Pain & NAD: Pain scores were lowest when patients/parents perceived non-active disease (NAD), aligning with true negative assessments by rheumatologists High Pain in True Positive: When rheumatologists confirmed active disease (true positive), patients/parents also reported high pain Discordant: Pain-Driven Overestimation: Patients/parents frequently overestimated disease activity, strongly influenced by pain High pain scores did not differentiate between patient/parent assessments that were true positive (rheumatologist agreed on AD) and false positive (rheumatologist did not agree on AD). Pain did not reliably distinguish active from inactive disease  Pain is a major problem in children with JIA and it is not always related to disease activity or damage. It can be caused by pathophysiological and psycho-emotional factors. Pain is related to well-being and should be a major concern in the treatment of children with JIA.True AD requires adjustment of medication, while other factors leading to AD being perceived requires education and proper counseling. | **MIXED** |
| Arnstad 2019 | MULTI | Pain at baseline, objective at follow-up: Baseline pain VAS vs JADI-A / JADI-E (at follow up) Baseline pain VAS vs Wallace remission status (at follow up) Baseline pain VAS vs disease extension in oligoarticular subgroup (at follow up)  Same time point: Follow up pain VAS vs Wallace remission status (at follow up) | No relationship / poor predictor: Baseline pain VAS vs JADI-A / JADI-E (long-term damage at follow-up). Baseline pain was a poor predictor of long-term articular or extra-articular damage, with poor discrimination for later damage. Odds of damage were similar regardless of baseline pain.  Concordant / predictive relationship: Baseline pain VAS vs Wallace remission status (follow-up). Higher baseline pain was associated with a greater likelihood of not achieving remission without medication at 8-year follow-up, with acceptable discrimination.  Baseline pain VAS vs disease extension in the oligoarticular subgroup (follow-up). In oligoarticular JIA, baseline pain was associated with a higher likelihood of disease extension or unfavourable categories at 8-year follow-up (48% with baseline pain vs 30% without).  Not reported / unclear: Follow-up pain VAS vs Wallace remission status (same time point). At 8-year follow-up, 57% reported pain and 57% were not in remission without medication; prevalence was reported without formal analysis of the relationship. Persistent pain despite apparent treatment response supports multifactorial contributors to pain, including biological and psychosocial factors. | **MIXED** |
| Arnstad 2020 | WALLACE | Pain VAS (current) vs Wallace criteria Pain VAS (prev week) vs Wallace criteria SF-36 pain intensity/interference (prev 4 weeks) vs Wallace criteria | Regardless of which measurement used for self-reported pain, participants with active disease reported the highest pain scores. On the contrary, participants in remission off medication reported pain scores more similar to controls. | **CONCORDANT** |
| Bandeira 2006 | JOINT COUNTS | Parent Pain VAS vs SJC (simple and weighted) Parent Pain VAS vs TEN/POMC (simple and weighted) Parent Pain VAS vs LROMC (simple and weighted) Parent Pain VAS vs AJC (simple and weighted) | Weighted joint counts generally showed stronger correlations with parent-reported pain intensity. Greater concordance between Parent Pain VAS and disease activity measures was observed in Cohort 2 (disease duration >5 years), with higher correlation magnitudes than in early disease.  Concordant: Cohort 1 Parent pain VAS vs simple TEN/POMC: r = 0.39 Parent pain VAS vs weighted TEN/POMC: r = 0.43  Cohort 2 (disease duration >5 years) Parent pain VAS vs simple SJC: r = 0.53 Parent pain VAS vs weighted SJC: r = 0.55 Parent pain VAS vs simple TEN/POMC: r = 0.48 Parent pain VAS vs weighted TEN/POMC: r = 0.53 Parent pain VAS vs simple LROMC: r = 0.33 Parent pain VAS vs weighted LROMC: r = 0.33 Parent pain VAS vs simple AJC: r = 0.51 Parent pain VAS vs weighted AJC: r = 0.60  Weak/inconsistent: Cohort 1 Parent pain VAS vs weighted SJC: r = 0.26  No relationship / association: Cohort 1 Parent pain VAS vs simple SJC: r = 0.14 (NS) Parent pain VAS vs simple LROMC: r = 0.19 (NS) Parent pain VAS vs weighted LROMC: r = 0.17 (NS) Parent pain VAS vs simple AJC: r = 0.16 (NS) Parent pain VAS vs weighted AJC: r = 0.23 (NS)  Cohort 1 had higher reported pain VAS despite the median counts of swollen, painful and active joints being comparable in the two cohorts. Weighted joint counts showed stronger correlation with parent-reported pain than unweighted counts. This may reflect better alignment of weighted scores with the impact of joint involvement on function, especially for joints with higher functional importance. | **MIXED** |
| Biancardi 2015 | IMAGING | Pain VAS vs US (Gray Scale and Power Doppler) | Parent Pain VAS showed moderate correlations with US findings in upper extremities (MCP, PIP, wrist, elbow) (r = 0.34-0.56) but low correlations for knees and ankles (r = 0.09-0.22)  Weaker agreement between US and clinical markers in the lower limbs (knee, ankle), as compared to upper limbs. | **MIXED** |
| Bloom 1995 | IMAGING | Pain (narrative/clinical) vs US | 5 of 6 cysts were painless despite visible US-confirmed synovial pathology | **DISCORDANT Pain < Disease activity** |
| Bou Antoun 2017 | IMAGING | Pt reported buttock pain and/or SI joint tenderness on exam (narrative/clinical) vs MRI detected sacroiliitis  Inflamm back pain (narrative/clinical) vs MRI detected sacroiliitis | Concordant: Buttock pain and/or SI joint tenderness vs MRI The presence of buttock pain or abnormal sacroiliac joint examination was a strong predictor of MRI-detected sacroiliitis. Sensitivity: 0.75 Specificity: 0.83 PPV: 0.75 NPV: 0.83  No relationship / association: Inflammatory back pain vs MRI Inflammatory back pain was not a significant predictor of MRI-detected sacroiliitis. Sensitivity: 0.12 Specificity: 0.75 PPV: 0.25 NPV: 0.56  Inflammatory back pain is less common in children than in adults, and sacroiliitis can remain clinically silent in children. Only a small proportion of patients with MRI-detected sacroiliitis reported a history of inflammatory back pain (13%). | **MIXED** |
| Brandon 2017 | COMPOSITE | Pain Interference vs JADAS3 | Pain Interference scores demonstrated strong construct validity by successfully discriminating between clinically defined disease states. The Mann-Whitney test showed that T scores for Pain Interference significantly differed (P<0.05) between children categorized as having inactive disease and those with active disease, for both patient and parent reports | **CONCORDANT** |
| Brandon 2021 | IMAGING | Change in pain vs change in MRI SIS (SPARCC Inflammation Score) | Correlation between change in patient-reported pain and change in SIS was weak (r = 0.14).  Complaints of inflammatory back pain by history and pain to palpation have relatively low positive predictive value in the pediatric population, making objective measures like SPARCC SIS critical. | **DISCORDANT Weak/inconsistent** |
| Carlsson 2023 | IMAGING | Pain VAS vs MRI | In JIA patients, orofacial pain intensity and pain during jaw function were related to TMJ bone marrow oedema, and in some cases erosion. However, there was substantial overlap in TMJ MRI findings between JIA patients and healthy individuals. Minor TMJ MRI changes may fall within the normal spectrum in children and adolescents and are not necessarily indicative of disease, potentially reflecting normal growth, disc displacement, trauma, or degenerative changes.  The overlap between JIA and healthy controls, together with weak or absent associations between clinical symptoms and MRI findings, suggests that TMJ MRI findings should be interpreted with caution in clinical practice. The limited clinical signs and symptoms observed may also reflect good disease control, as patients were well managed by paediatric rheumatologists and orofacial pain specialists, More extensive systemic pharmacological treatment regimens are a likely reason for lower observed erosion and synovial thickening compared to previous studies | **MIXED** |
| Chaplin 2018 | JOINT COUNTS | Pain APPT via AJC | Pain intensity showed a weak-to-moderate association with active joints (rs = 0.27). Active joint count alone explained little variance in pain, with most of the association mediated by illness perceptions; identity and consequence beliefs accounted for 70.5% of the relationship between active joints and pain intensity. These findings indicate that clinical disease activity does not fully explain pain and support a biopsychosocial model, highlighting illness beliefs as a potential intervention target. | **DISCORDANT Weak/inconsistent** |
| Collin 2024 | IMAGING | Self-reported TMJ pain vs TMJ deformity on CBCT | Concordant: Self-reported TMJ pain was associated with TMJ deformity on CBCT at baseline, with a higher prevalence of deformity among children reporting TMJ pain. TMJ pain and dysfunction were predictive of baseline TMJ deformities.  No relationship/association: Previous TMJ pain or dysfunction did not predict longitudinal changes in TMJ morphology. The lack of association over time may reflect the small number of joints showing progression, limited temporal sampling, and high levels of disease control with bDMARD therapy, which may have reduced detectable structural change. | **MIXED** |
| Coulton 1987 | MULTI | Pain intensity (AIMS) vs AJC Pain intensity (AIMS) vs Active/Inactive JA group classification | Pain dimension vs Joint count (AJC): Active JA: Concordant (r = 0.35) Inactive JA: NA (lack of variance)  Pain dimension vs Active/Inactive JA group classification: Concordant (77.63% correctly predicted) | **CONCORDANT** |
| de Oliveira Sato 2011 | DAMAGE | Pain VAS vs JADI-A Pain VAS vs JADI-E | Pain VAS vs JADI-A: rs -0.03 NS Pain VAS vs JADI-E: rs -0.03 NS | **DISCORDANT No relationship/association** |
| de Vos 2023 | JOINT COUNTS | SPJC vs AJC Pain VAS vs AJC | The association between SPJC of 0 and AJC of 0 was statistically significant (χ² = 187.8). 83% of people who reported an SPJC of 0 had an AJC of 0.  Participants with active joints (AJC > 0) reported higher Pain VAS compared to those with AJC = 0.  Discordant: 50 participants (17.5% of those reporting SPJC = 0) stated SPJC = 0 but had AJC > 0. Relying solely on patient-reported SPJC risks missing 7% of patients needing therapy adjustments.  For the discordant group (SPJC = 0 but AJC > 0), only a small percentage (8%) had an active joint that cannot be reported in the JAMAR, implying other reasons for discrepancy.  Participants with Poly JIA were more often incorrect about having no active joints (20 participants, 40% of the discordant group). | **CONCORDANT** |
| Dekker 2010 | COMPOSITE | Pain VAS overall body vs JADAS71 Pain VAS feet vs JADAS71 Pain VAS feet (Dr report) vs JADAS71 | VAS overall body pain vs JADAS71 r = 0.80 VAS pain feet vs JADAS71 r = 0.70 VAS pain feet (Dr reported) vs JADAS71 r = 0.74 | **CONCORDANT** |
| Eboli 2022 | IMAGING | Pain VAS vs US | No strong agreement between US and clinical data in detecting signs of TMJ involvement.  Further studies with larger cohorts and involving the use of MRI are needed to clarify the role of US in the assessment of TMJ arthritis in JIA. | **DISCORDANT Weak/inconsistent** |
| Elfving 2023 | BLOOD BIOMARKERS (OTHER) | Pain VAS vs IL-18R1 Pain VAS vs TGFβ Pain VAS vs GDNF Pain VAS vs FGF21 Pain VAS vs VEGFA Pain VAS vs S100A12 | No relationship:  For well-known inflammatory markers like IL-6, S100A12, TNFα, and IL-1, no statistically significant relationship or association with Pain VAS Concordant: In the unadjusted analyses, these three proteins showed statistically significant correlations with pain VAS scores: GDNF: correlation coefficient 0.33 TGFβ: correlation coefficient 0.34 IL-18R1: correlation coefficient 0.37 Pain in JIA is often independent of inflammation; inflammatory markers (e.g., IL-6) correlated with disease activity but not pain. Conversely, GDNF correlated with pain but not inflammation, suggesting non-inflammatory pain mechanisms. | **MIXED** |
| Elliott 1997 | MULTI | Pain VAS vs Fever Pain VAS vs TJC Pain VAS vs PhGA Pain VAS vs ESR Pain VAS vs CRP Pain VAS vs SAA Pain VAS vs IL-6 Pain VAS vs sTNFR p55 and p75 Pain VAS vs IL-1ra Pain VAS vs Immunoreactive TNF-α | Discordant Pain > Disease activity:  ESR, CRP, SAA, IL-6,sTNFR p55/75, IL-1ra Discordant Pain<Disease activity: TNF-α Concordant: PhGA, TJC  While systemic disease features were transiently controlled, the administration of cA2 did not result in an improvement in the patient's arthritis, which included joint pain and tenderness. This was in marked contrast to responses observed in adult rheumatoid arthritis. TNF-α might be a less important inflammatory mediator in the articular disease of systemic JCA compared to adult RA. | **MIXED** |
| Esbersen 2018 | COMPOSITE | Pain VAS vs JADAS-27 | Disease activity (JADAS27) was significantly positively correlated to pain intensity. Higher pain intensity is related to higher disease activity in JIA. | **CONCORDANT** |
| Ferrazzo 2019 | IMAGING | Pain (narrative/clinical) vs MRI/CT | TMJ arthritis in JIA may be asymptomatic; lack of early diagnosis can lead to permanent skeletal deformity due to altered mandibular growth. | **DISCORDANT Pain < Disease activity** |
| Flatø 2002 | IMAGING | Inflammatory back pain (narrative/clinical) vs Radiographic sacroiliitis | At follow-up (after a median disease duration of 14.9 years), inflammatory back pain was more frequently observed in patients with radiographic sacroiliitis (65%) than in those without sacroiliitis (4%).  At 6 months of disease duration (onset), inflammatory back pain was also more frequent in patients who later developed sacroiliitis (10%) than in those who did not (1%). | **CONCORDANT** |
| Gestler 2022 | CLINICAL EXAMINATION (UNDEFINED) | Pain (narrative/clinical) via residual complaints questionnaire vs assessment of inactive disease (lab and clinical) | Discordant for 17.9% of children reported ongoing arthralgia despite inactive disease  Concordant for 82.1%  Higher-than-expected prevalence of residual complaints in JIA patients with inactive disease | **MIXED** |
| Gorczyca 2017 | BLOOD BIOMARKERS (OTHER) | Pain VAS vs MRP8/MRP14 | MRP8/14 positively correlated with pain VAS | **CONCORDANT** |
| Guzman 2017 | JOINT COUNTS | Pain VAS prev week vs AJC Severe Controlled Pain VAS prev week vs AJC Severe Persisting | Stronger association (Severe Controlled group, 9%): Pain intensity (VAS) decreased in parallel with reductions in AJC Spearman correlation = 0.46 Weaker association (Severe Persisting group, 11.5%): Pain remained higher relative to joint activity, showing a weaker relationship with AJC Spearman correlation = 0.30  Post hoc analyses found little evidence that a chronic pain syndrome, rather than persistent disease activity, was the main reason for the severe persisting course. | **MIXED** |
| Hara 2017 | MULTI | Pain (narrative/clinical) vs PD US Pain (narrative/clinical) vs XR Pain (narrative/clinical) vs serum MMP-3 level Pain (narrative/clinical) vs clinical exam | Concordant: Pain vs clinical exam (case 1 no pain= no abnormalities found on examination) Pain vs PD US (case 3 wrist pain = mod grade PD US signal in the wrist joint) Discordant - Pain < Disease activity: Pain vs PD US (case 1 no pain but prominent PD signal in both shoulder joints Discordant - Pain > Disease activity: Pain vs serum MMP-3 Pain vs XR (case 3 wrist pain but serum MMP-3 level and X-ray findings were normal)  Joint ultrasonography is a sensitive technique for detecting findings consistent with arthritis that may be difficult to detect by physical examination, conventional lab tests (MMP-3), or X-ray. In case 3, US findings (moderate PD signal) led to a change in biological DMARD even though serum MMP-3 and X-ray findings were normal, supporting its utility when pain is present but other markers are normal. | **MIXED** |
| Hogewe 1995 | MULTI | Pain VAS vs C-reactive protein (CRP) Pain VAS vs Active Joint Count (AJC) Pain VAS vs Swollen Joint Count Pain VAS vs Ritchie Index | Pain VAS (previous week) showed moderate associations with CRP (r = 0.58), active joint count (r = 0.43), swollen joint count (r = 0.43), and the Ritchie Index (r = 0.47). | **CONCORDANT** |
| Hospach 2014 | IMAGING | Pain (narrative/clinical) vs MRI | At onset, All 13 patients, including those with pain, showed joint enhancement on MRI. 3 of the 5 patients with pain (60%) showed skeletal changes (ankylosis, erosions, or narrowing of the spinal canal) on MRI.  At follow-up, pain was no longer reported by any patient yet 6 of 13 (46%) still showed skeletal changes (ankylosis, erosions, or narrowing of the spinal canal) on MRI. Inflammation sites (hyperintensity, enhancement) were still present in a majority of patients (77% and 85% respectively).  At the onset of cervical spine involvement, all 13 patients (100%) showed LROM, whereas only 5 of them (38%) complained of pain LROM and MRI at Follow-up vs Pain: At follow-up, pain was no longer reported by any patient, but LROM persisted in 9 out of 13 patients (69%), with 6 of these 9 patients (46% of the total cohort) still showing skeletal changes (ankylosis, erosions, or narrowing of the spinal canal) on MRI.LROM as a leading clinical sign of cervical spine arthritis- patient history and pain is not a sufficient criterion for cervical spine involvement because LROM was present in all patients at diagnosis while only a minority complained of pain. This indicates a stronger and more consistent association between LROM and objective disease activity on MRI than with pain. | **DISCORDANT Pain < Disease activity** |
| Hu 2019 | BLOOD BIOMARKERS (OTHER) | Pain (narrative/clinical) vs Histone | Histone levels significantly affected other clinical manifestations (fever, rash, hepatosplenomegaly, serositis) but not joint pain.  Serum histone levels were related to most clinical signs and symptoms of sJIA but not joint pain, suggesting histones' role in systemic activity might be distinct from direct pain mediation in this context. | **DISCORDANT No relationship/association** |
| Ilowite 1992 | THERMOGRAPHY | Self-report pain VAS vs thermography findings (dT values) Parent report pain VAS vs thermography findings (dT values) Dr report pain VAS Self report PPQ vs thermography findings (dT values) | Concordant: Parent pain VAS vs joint temperature (r = 0.58) Doctor pain VAS vs joint temperature (r = 0.50)  In children with pauciarticular onset disease, parent VAS scores correlated with dT values (r = 0.63) For younger patients (<7.86 years; median split), patient VAS scores vs joint temperature values (r = 0.73)  No relationship/association:  Patient (child) pain intensity ratings vs joint temperature were not statistically significant overall (r = 0.32). In older patients, this correlation was lower and negative (r = −0.24)  No significant differences in dT values were found between children who chose adjectives reflecting heat on the PPQ (“burning,” “hot,” “warm”) and those who did not.  Mixed: For parent pain VAS vs joint temperature, a discrepancy was noted between younger (r = 0.70) and older (r = 0.24) patients. In younger children, the relationship between joint inflammation and perceived pain was fairly strong (dT values accounted for 53% of variance). In contrast, in older children, the correlation was lower and negative, possibly due to more socio-emotional factors affecting pain perception. | **MIXED** |
| Katsicas 2017 | BLOOD BIOMARKERS (OTHER) | Lumbar pain (narrative/clinical) vs Th17 | Concordant: Lumbar Pain (LP): Th17 cells showed a strong correlation with Lumbar Pain (r=0.83, p=0.0001) despite not correlating with AJC Not reported: Th1 or Th17 vs Pain VAS/SIJ pain | **MIXED** |
| Katsicas 2018 | IMAGING | Pain VAS vs MRI  Sacroiliac pain (SIP) vs MRI  Lumbar pain (LP) vs MRI | No relationship/association between: Pain VAS and MRI Sacroiliac pain (SIP) and MRI Lumbar pain (LP) and MRI  Not reported (the conclusion focuses on SPARCC SIS association with age/disease duration) | **DISCORDANT No relationship/association** |
| Khanom 2022 | COMPOSITE | Pain dimension from CHU9D vs cJADAS71 | Moderate correlation: Pain (CHU9D) vs cJADAS: r = 0.39 cJADAS predicted Pain (CHU9D): β = 0.34  Low explanatory power (discordance): Disease activity explained only 15.3% of CHU9D total variance. Pain and other symptom flares should be assessed as separate entities that are not fully accounted for by clinical measures of inflammation. In the case of a symptom flare where disease activity is low or absent, anti-inflammatory medication may not be effective; therefore, alternative treatments, such as physiotherapeutic or occupational approaches, might be more appropriate. | **MIXED** |
| Kovalchuk 2018 | MULTI | Only significant correlations (p < 0.05) reported: Child Pain VAS vs AJC Child Pain VAS vs PhGA Child Pain VAS vs DAS Child Pain VAS vs DAS28 Child Pain VAS vs SDAI Child Pain VAS vs CDAI Child Pain VAS vs Number of limited joints Child Pain VAS vs Number of painful joints Child Pain VAS vs Number of swollen joints Child Pain VAS vs ESR Child Pain VAS vs CRP Child Pain VAS vs CD3+ Child Pain VAS vs CD4+  Parent Pain VAS vs AJC Parent Pain VAS vs PhGA Parent Pain VAS vs DAS Parent Pain VAS vs DAS28 Parent Pain VAS vs SDAI Parent Pain VAS vs CDAI Parent Pain VAS vs Number of limited joints Parent Pain VAS vs Number of painful joints Parent Pain VAS vs Number of swollen joints Parent Pain VAS vs ESR Parent Pain VAS vs CRP Parent Pain VAS vs CD3+ Parent Pain VAS vs CD4+ | Concordant (Moderate Correlation 0.4-0.7): Child Pain VAS vs PhGA: 0.69 Child Pain VAS vs DAS: 0.62 Parent Pain VAS vs PhGA: 0.57 Child Pain VAS vs DAS28: 0.55 Child Pain VAS vs SDAI: 0.55 Child Pain VAS vs CDAI: 0.55 Parent Pain VAS vs DAS: 0.54  Parent Pain VAS vs DAS28: 0.48 Parent Pain VAS vs SDAI: 0.48 Parent Pain VAS vs CDAI: 0.49 Child Pain VAS vs Painful JC: 0.42 Weak/Inconsistent Findings (Low Correlation < 0.4): Parent Pain VAS vs Painful JC: 0.37 Child Pain VAS vs CD4+: -0.36 Child Pain VAS vs AJC: 0.36 Child Pain VAS vs CD3+: -0.34 Child Pain VAS vs Swollen JC: 0.31 Parent Pain VAS vs AJC: 0.31 Parent Pain VAS vs Swollen JC: 0.26 No Relationship/Association (Not statistically significant): Child Pain VAS vs Limited range JC Child Pain VAS vs ESR Child Pain VAS vs CRP Parent Pain VAS vs Limited range JC Parent Pain VAS vs ESR Parent Pain VAS vs CRP Parent Pain VAS vs CD3+ Parent Pain VAS vs CD4+  Parents based their PaGA assessment on pain complaints and physical/psychosocial limitations. Physicians based their PhGA on joint counts and laboratory tests. This difference in criteria could influence correlations with specific objective measures. | **MIXED** |
| Küseler 2005 | IMAGING | Pain symptom score vs MRI | No correlation between total clinical and MRI scores (r² = 0.03). Symptoms (e.g., joint pain at rest or on motion) were reported in 30% of visits, but 93% of TMJs showed synovial enhancement on MRI. Only one child had no symptoms and a normal MRI.  TMJ related symptoms and clinical signs are not characteristic of JIA, leading to early TMJ inflammation often remaining subclinical and active for years without presenting symptoms. | **DISCORDANT Pain < Disease activity; No relationship/association** |
| Learoyd 2019 | MULTI | At individual clinic visits: Pain VAS (present/current) vs PhGA  Pain VAS (present/current) vs AJC  Pain VAS (present/current) vs SJC Pain VAS (present/current) vs LROMC  Across trajectories: Pain trajectory vs Baseline PhGA  Pain trajectory vs Baseline AJC Pain trajectory vs Baseline SJC  Pain trajectory vs Baseline LROMC  Pain trajectory vs Average PhGA  Pain trajectory vs Average AJC Pain trajectory vs Average SJC Pain trajectory vs Average LROMC | Concordant: Single clinic visit pain associated with PhGA Higher average PGA at study onset in variable/high pain trajectories than low pain High pain trajectory associated with higher SJC at study onset High pain trajectory associated with more LROMC at study onset Variable pain trajectory associated with more LROMC Joint type patterns (active, swollen, limited) differed, with variable/high pain groups showing more swollen and limited joints  Discordant (Pain > Disease Activity): High pain trajectory less likely to have active joints at study onset Variable pain trajectory showed a trend towards fewer active joints  No relationship/association: Single clinic visit pain showed no association with concurrent active, swollen, or limited joints Average AJC showed no significant difference across pain trajectories  Pain vs current inflammation (discordant/no association): Patients showed individual pain sensitivity independent of concurrent joint inflammation Pain trajectory was not dependent on current arthritic activity (active joint count) These findings suggest high pain trajectories can exist despite less active arthritis  Role of prior arthritic activity (priming): Patients with high pain had less active arthritis but more symptoms associated with prior arthritic activity at or before study onset (swollen and limited joints) The mechanism may involve prolonged central sensitisation not related to current inflammation i.e., prior, rather than current, arthritic activity may explain high pain sensitivity  Individual pain sensitivity: Individuals differed in pain sensitivity This sensitivity was not solely explained by current inflammatory status It led to persistently higher or more prolonged pain in some individuals Differences in innate sensitivity to inflammatory pain resulted in low or high pain trajectories Proposed neurobiological mechanisms included genetic and epigenetic processes, alterations in brain networks, and priming of the pain system by previous pain and trauma. | **MIXED** |
| Leksell 2012 | IMAGING | Severe symptoms vs XR Severe impact on daily life vs XR Facial pain > 2 times/week vs XR Facial pain ≤ 2 times/week vs XR Pain at chewing vs XR | No statistically significant association between subjective orofacial pain/symptoms and TMJ structural changes on X-ray  It is not possible to predict disabling orofacial pain and dysfunction based solely on objective findings. | **DISCORDANT No relationship/association** |
| Lomholt 2013 | CLINICAL EXAMINATION + LABORATORY PARAMETERS (defined composite) | FPS-R (diary for 2 weeks) vs Composite disease activity score (composite; criteria specified) | 1. Discordant - Pain > Disease activity Overall Group Comparison:  Anti-TNF group (n=41) had significantly lower disease activity (mean 0.65) vs standard treatment group (n=50) (mean 2.8)  However, average pain intensity was similar (Anti-TNF: mean 1.03; Standard: mean 0.86). A total of 28 out of 41 anti-TNF children reported pain, while 13 out of 41 were pain-free Among Children Reporting Pain:  Anti-TNF group (28 out of 41 total anti-TNF children) had significantly lower disease activity (mean 0.68) vs standard group (49 out of 50 total standard treatment children) (mean 2.90) Despite lower disease activity, these 28 anti-TNF children reported significantly higher pain intensity (mean 1.50) than the 49 standard treatment children who reported pain (mean 0.88) 2. No relationship/association Within Anti-TNF Group: When comparing anti-TNF children with pain (28 out of 41) to pain-free anti-TNF children (13 out of 41), no differences were found in disease activity This indicates that pain presence/absence was not associated with objective disease activity in this group.  Possible biological factor: Central sensitization causing hyperalgesia Possible psychological and social factors: For children on anti-TNF agents, experiencing pain was associated with more maladaptive psychological responses, including magnifying their pain, fearing harm, and feeling functionally impaired by it, irrespective of their objective disease activity. | **DISCORDANT Pain > Disease activity; No relationship/association** |
| Magni-Manzoni 2005 | CLINICAL EXAMINATION + LABORATORY PARAMETERS | Parent pain assessment (VAS) vs flare | Parent pain assessment (VAS): Showed "poorest performances" in detecting a disease flare, with an Effect Size (ES) of 0.47 and a Standardized Response Mean (SRM) of 0.40 | **DISCORDANT Weak/inconsistent** |
| Magni-Manzoni 2009 | IMAGING | Parent pain VAS vs US | Relationship between Parent pain VAS and US: Spearman's correlations were all poor, ranging from -0.07 to -0.18 Relationship between TEN/POM and US: Spearman's correlations were all poor, ranging from 0.15 to 0.25 Kappa values for agreement between TEN/POM and US findings were fair: 0.35 for synovial hyperplasia, 0.32 for joint effusion, and 0.37 for PD signal  US findings were not correlated with subjective parent's ratings. This is not surprising because these measures assess disease constructs that are only partially related to joint inflammation Joint swelling is considered a more reliable clinical indicator of joint synovitis in JIA than pain on motion or tenderness. | **DISCORDANT Weak/inconsistent** |
| Mahmood 2014 | IMAGING | Pain (narrative/clinical) report vs MRI | A subset (25%) of patients had no pain despite an abnormal cervical spine MRI  Cervical spine involvement may be clinically silent and potentially only present clinically when severe and disabling; early identification through MRI is important for early therapeutic intervention and potentially better outcome. | **MIXED** |
| Malattia 2010 | IMAGING | Pain VAS prev week (average) vs MRI | Concordant for wrist (Pain VAS vs MRI): REE (MRI dynamic parameter) correlated with pain assessment scale (rs = 0.63)  No relationship/association for hip (Pain VAS vs MRI): No correlation was reported between dynamic MRI parameters (ME, MV, REE) and pain intensity for hip arthritis; only LROM was correlated with MRI (rs = 0.69) Hip disease patients showed lower disease activity on dynamic MRI parameters than wrist arthritis patients Clinical examination may not adequately detect low inflammation in hip joints Prior studies confirm that clinical examination is inadequate for assessing hip joint disease activity, risking structural damage despite apparent clinical control. | **MIXED** |
| Malleson 2004 | MULTI | Pain VAS vs PGA Pain VAS vs AJC | Disease-related factors explain only a small proportion of the variation in pain scores. Overall Cohort: In the full multivariate model, PGA explained only 6.5% of the variation in pain scores (5.3% for scores 2 or 3 and 1.2% for score 1) Active Joint Count (AJC): Showed a statistically significant univariate correlation with pain (Spearman's coefficient 0.424 in the overall cohort, however, its predictive power varied by age group in multivariate analysis: In the 8-15 year age group, AJC explained 8.4% of the pain variation and was an independent predictor In the >16 year age group, AJC was eliminated from the regression model, indicating it was not an independent predictor of pain. Disparity with other studies may be due to "present pain" vs "worst pain" assessment. Pain in JRA is a multifaceted condition that cannot be simply explained by demographic or disease-related factors. These findings stress the importance of looking further at other factors, such as coping, mood, and family structures. | **DISCORDANT Weak/inconsistent** |
| Mannion 2019 | PhGA | Pain score vs PhGA | Weak correlation between physician global and pain score (R=0.24) | **DISCORDANT Weak/inconsistent** |
| Mannion 2022 | MULTI | PI vs cJADAS Inactive Disease PI vs cJADAS Low Disease Activity (LDA) PI vs ACR provisional criteria for Clinical Inactive Disease (ACRCID) PI vs ACR preliminary criteria for Clinical Inactive Disease (Wallace criteria) | Similar worsening frequency of PI across all groups (1/3 PI improved, 1/3 PI worse, 1/3 PI unchanged althouogh cJADAS ID and cJADAS LDA were associated with relative change in pain interference | **MIXED** |
| McGrath 2023 | JOINT COUNTS | Pain VAS over time vs Baseline active joint and enthesitis count | Higher enthesitis and AJC were seen in the persistent pain group; enthesitis remained a strong independent predictor. Female sex, more widespread enthesitis, and lower quality of life at baseline were key predictors of persisting pain in JIA. Authors suggest that these children may be candidates for early pain management interventions. They emphasise the need for future research into psychological and contextual predictors, including coping and central sensitisation, which were not assessed in this study. | **CONCORDANT** |
| McLellan 2023 | IMAGING | Pain (narrative/clinical) vs MRI | 78% of TMJ scans were abnormal with 53% indicating joint damage. 29% of patients did not have pain, but other symptoms (restricted movement, jaw swing) | **MIXED** |
| Melchiorre 2010 | IMAGING | Pain (narrative/clinical) vs JE (joint effusion) on US | Only 2 (4.3%) out of 46 children with US evidence of Joint Effusion (JE) were symptomatic (defined to include recurrent pain); the other 44 (95.6%) did not complain of joint pain  TMJ involvement in oligoarticular JIA is often asymptomatic and consequently overlooked. Clinical assessment of TMJ is insufficient because its involvement is often asymptomatic. | **DISCORDANT Pain < Disease activity** |
| Miller 2011 | MULTI | Pain NRS vs PhGA Pain NRS vs AJC | Concordant: Pain NRS vs PhGA (all mod to strong) ERA: strong 0.89 Systemic: strong 0.76 RF- poly: mod 0.55 RF+ poly: mod 0.48 Oligo-p: mod 0.45 Weak/Inconsistent: Pain NRS vs AJC Narrative summary: correlations between pain scores and joint counts in all JIA subtypes tended to be lower than other correlations  Correlations between pain scores and joint counts in all JIA subtypes tended to be lower than other correlations (data not shown), possibly because pain assessment during clinic visits can be insensitive. | **MIXED** |
| Min 2020 | COMPOSITE | Pain VAS vs cJADAS | Pain VAS demonstrated a positive correlation with disease activity (R= 0.58) | **CONCORDANT** |
| Minden 2014 | PhGA | Pain NRS vs PhGA | During pediatric care, the relationship between physician and patient scores for disease activity (which includes pain) was reported as a correlation of 0.56. During adult care, this correlation weakened to 0.51. In adult care, patients reported higher pain levels (mean 2.8) and a higher percentage experiencing pain (78%) at last follow-up, while adult healthcare providers rated the patients' disease activity lower (mean 1.9) than the patients did themselves. | **MIXED** |
| Miyamae 2019 | MULTI | Pain (narrative/clinical) vs ESR Pain (narrative/clinical) vs CRP Pain (narrative/clinical) vs MMP-3 Pain (narrative/clinical) vs US Pain (narrative/clinical) vs Clinical examination Pain (narrative/clinical) vs DAS28-ESR | No pain despite active disease  Neural mechanisms underlying pain in early childhood, particularly the developmental stage of pain perception and verbalization in younger children, may explain the lack of reported pain despite active disease. They also highlight that the absence of pain led to delayed diagnosis in these cases. | **DISCORDANT Pain < Disease activity** |
| Mohammed 2012 | MULTI | Pain VAS vs MRI (Total MRI score) Pain VAS vs Clinical examination  Pain VAS vs Disease activity (Clinical disease activity score) TMJ symptoms (presence/absence) vs MRI abnormalities (presence/absence) | No relationship/association: There was no association between patient-reported TMJ symptoms (presence/absence) and MRI TMJ abnormalities (χ² = 5.3).  Discordant – Pain < Disease activity: TMJ involvement was often asymptomatic, with symptoms underestimating the inflammatory state.  For those with pain: Concordant: A positive correlation was found between pain severity (Pain VAS) and total MRI score (r = 0.72). A negative correlation was found between pain severity (Pain VAS) and maximal interincisal opening (MIO) (r = −0.72). Mean pain scores were higher in patients with active disease than in those with inactive disease.  TMJ involvement in JIA is often asymptomatic and the joint is difficult to examine. Symptoms underestimate the inflammatory state of the TMJ, and the absence of symptoms or TMJ examination abnormalities does not exclude TMJ disease. Clinical examination appears more reliable than symptom enquiry alone, as patients with clinical findings also showed pathological signs on post-contrast MRI. | **MIXED** |
| Mosa 2021 | IMAGING | Pain (narrative/clinical) vs US | The bicipital synovial cyst presented as a painless swelling despite the high systemic inflammation and imaging evidence of tenosynovitis and cystic lesions.  The case emphasized the concept of "non-articular synovitis" as the sole musculoskeletal manifestation of SJIA at disease onset, with the bicipital synovial cyst presenting as a painless swelling despite high systemic inflammation and disease activity. | **DISCORDANT Pain < Disease activity** |
| Mountain 2025 | COMPOSITE | Pain VAS vs cJADAS | Overall association (n = 187 cohort): A moderate-to-strong positive association was observed between pain severity and cJADAS-10 (r = 0.506)  Subgroup stratification (discordance/concordance): Two concordant groups were identified (Low Pain/Low Disease; High Pain/High Disease) and two discordant groups (High Pain/Low Disease; Low Pain/High Disease)  The observed discordance lay in participants’ personal models of pain beliefs, with the High Pain/Low Disease trajectory (the primary discordant group) more likely to perceive greater threat and less control over pain than the Low Pain/Low Disease reference group. Similarly, the High Pain/High Disease group was more likely to perceive greater threat, experience more negative emotional representations, and report less control over pain than the reference group.  High pain severity, regardless of underlying clinical disease activity, was strongly associated with adverse pain belief constructs, indicating that subjective pain experience is influenced by psychological factors such as beliefs about pain threat and controllability rather than objective clinical disease measures. | **MIXED** |
| Naddei 2023 | PhGA | Pain VAS vs PhGA (inactive disease) | Pain is a main determinant of this discordance  Many patients mark the PaGA>1 in the absence of active disease according to the physician, showing that to patients abrogation of inflammation not always mean remission. Pain and impairment of quality of life appear to be the main determinants of this discordance, suggesting that PaGA reflects many aspects of the disease burden, including non-inflammatory pain, functional ability, treatment burden and psychosocial aspects. | **DISCORDANT Pain > Disease activity** |
| Nguyen 2024 | MULTI | Parent reported pain VAS/NRS prev week vs Wallace inactive disease status Parent reported pain VAS/NRS prev week vs. Inactive Disease (cJADAS10 criteria) Parent reported pain VAS/NRS prev week vs. Minimally Active Disease (cJADAS10 criteria) | Pain vs Wallace: Parent-reported minimal pain (Pain < 1) attainment (63%) lagged significantly behind Inactive Disease (Wallace criteria) attainment (83%) in the later cohort Pain vs cJADAS: Attainment increases for cJADAS10 ID (50% - 69%) and MAD (69% - 84%) were substantially larger than concurrent increases for parent-reported Pain < 1 (57% - 63%) within 70 weeks  Improvements in pain and global scores were smaller than improvements in disease activity Subjective improvement potentially lagging behind changes in disease activity Measurement differences between cohorts Patient/parent expectations or psychosocial effects Disconnect between physician-rated and patient/parent-reported outcomes. | **DISCORDANT** |
| Nistala 2007 | IMAGING | Hip pain (narrative/clinical history) vs MRI | Weak/inconsistent: Hip pain associated with MRI activity score in univariate analysis (P=0.2) but did not make a statistically significant contribution in a multiple regression model predicting MRI scores  Findings of inflammation without clinical signs are likely to reflect the high sensitivity of MRI. Clinical assessment of active hip arthritis has a limited relationship with MRI features of inflammation particularly when co-existent damage is present. | **DISCORDANT No relationship/association** |
| Oen 2009 | MULTI | Pain VAS vs AJC Pain VAS vs PhGA Pain VAS vs ESR Pain VAS vs CRP | Pain VAS vs AJC: r = 0.38 (n = 321) Pain VAS vs PhGA: r = 0.28 (n = 319) Pain VAS vs ESR: r = 0.17 (n = 291) Pain VAS vs CRP: r = 0.14 (n = 251)  Pain was weakly associated with objective measures of disease activity. AJC showed the strongest correlation with pain (r = 0.38), followed by PhGA (r = 0.28), ESR (r = 0.17), and CRP (r = 0.14).  ESR and CRP were weakly associated with pain and were not predictive in multivariable models. Pain correlated more strongly with patient-reported outcomes (e.g. JAQQ, C-HAQ) than with physician-assessed or objective clinical measures. | **DISCORDANT Weak/inconsistent** |
| Oen 2018 | MULTI | Pain VAS prev week vs AJC  Pain VAS prev week vs PhGA (Time to reach best possible scores) | Pain persisted longer even after clinical indicators (AJC, PhGA) had improved. Improvement in HRQoL is slower and lags behind improvement for disease activity, pain, and disability measures by 1-4 years.  Pain and HRQoL improved later than disease activity. This may reflect a lag between inflammation control and perceived well-being, supporting the idea that HRQoL (including pain) may improve more slowly due to its multidimensional nature. | **MIXED Pain > Disease activity** |
| Oen 2021 | CLINICAL EXAMINATION + LABORATORY PARAMETERS (defined composite) | Pain VAS (prev week) vs Disease activity (latent variable indicated by ESR, CRP, Active joint count, PhGADA) | Moderate positive correlation between disease activity and pain at all timepoints: Diagnosis: β = 0.43 3-9 months: β = 0.45-0.48 Flare: β = 0.62-0.64 Pain and disease activity were more strongly concordant at time of flare (β = 0.62-0.64) than at diagnosis or early post-diagnosis.  Pain was the strongest direct contributor to lower health-related quality of life and also mediated the effects of disease activity and function. Authors emphasised the need to address pain early and independently of inflammation. | **CONCORDANT** |
| Palmisani 2006 | MULTI | Parent Pain score vs PhGA Parent pain vs AJC Parent pain vs TEN/POMC Parent pain vs SJC Parent pain vs LROMC Parent pain vs ESR Parent pain vs CRP Parent pain vs Poznanski score | Concordant: Parent pain vs PhGA: Moderate correlation across all cohorts: Early (0.42), Advanced (0.68), Longstanding (0.47) Parent pain vs TEN/POMC: Moderate correlation across all cohorts: Early (0.49), Advanced (0.59), Longstanding (0.47) Notably, this was the sole articular variable correlated with parent pain in early disease Mixed Relationships (varying levels of association or different patterns of correlation across the disease duration cohorts): Parent pain vs No. active joints (AJC): Low correlation in early disease (0.35) Moderate correlation in advanced disease (0.54) and longstanding disease (0.41) Parent pain vs Poznanski score (radiographic damage): Moderate negative correlation in early disease (-0.47) Low correlation in advanced disease (-0.12) and longstanding disease (0.04) Parent pain vs ESR: Moderate correlation in early disease (0.46) Low correlation in advanced disease (0.38) and longstanding disease (0.23) Weak/Inconsistent: Parent pain vs CRP: Low correlation across all cohorts: Early (0.35), Advanced (0.26), Longstanding (0.34)  Influence of other disease-related factors: The parent's perception of pain might be influenced by factors beyond just joint pain, such as the intensity and duration of morning stiffness General factors related to the child: This could include the child's ability to cope with pain or the influence of pain on the child's psychosocial functioning Difference in assessment timeframe: The parent rates the child's pain as experienced in everyday living in the week preceding the visit, whereas the physician assesses the level of pain on the day of the visit as elicited by pressure or passive motion This difference in the time period being assessed could contribute to the varying perception. | **MIXED** |
| Papsdorf 2011 | WALLACE | Pain VAS vs Wallace criteria for Inactive Disease (ID) | Pain and disease activity were measured at the same time. When patient-reported pain was added to remission criteria, remission rates dropped by 75%, indicating persistent pain despite clinical disease control. When using modified remission criteria, including the patients' global assessment of pain, the number of responders in ID decreased markedly. Thus a normalization of the laboratory inflammation parameters and the disappearance of clinical symptoms do not necessarily mean a subjective sensation of absence of all symptoms, especially of pain. In the current definitions of remission, the patients' pain is excluded [13]. The reason for this exclusion may have been the fact that it cannot be clearly determined to what extent pain is due to damage occurring after a longer disease duration (which cannot be influenced by anti-inflammatory agents), or due to ongoing inflammation indicating ongoing active disease. | **DISCORDANT Pain > Disease activity** |
| Pedersen 2008 | MULTI | TMJ pain (narrative/clinical) vs Clinical examination  TMJ pain (narrative/clinical) vs MRI TMJ pain (narrative/clinical) vs OPG | Subjective symptoms underestimate inflammatory TMJ findings  Inflammation seems to start early without causing symptoms Changes due to resorption/remodeling and resulting temporomandibular dysfunction cause symptoms at a later age | **DISCORDANT Pain < Disease activity** |
| Pinto 2018 | MULTI | Pain (narrative/clinical) vs XR Pain (narrative/clinical) vs CT/CBCT Pain (narrative/clinical) vs MRI Pain (narrative/clinical) vs clinical examination | No pain despite imaging showing severe destruction of both condyles  The clinical symptoms of juvenile TMD in JIA can change and are difficult to assess, especially in small children, as pain is often reported by parents. Prior antirheumatic treatment can also camouflage symptoms. | **DISCORDANT Pain < Disease activity** |
| Portelli 2014 | IMAGING | Jaw pain (specific questionnaire and narrative/clinical ) vs MRI | Pain did not significantly correlate with MRI findings of TMJ effusion or structural changes. Condylar and mandibular alterations were observed more often in asymptomatic patients than in those reporting pain.  TMJ clinical evaluation was quite difficult, especially in young children, because symptoms, such as pain, could often only be evaluated indirectly, by asking the parents. Furthermore, some clinical symptoms could be masked by antirheumatic therapy. | **DISCORDANT Pain < Disease activity** |
| Quilis 2024 | BLOOD BIOMARKERS (OTHER) | Pain VAS prev week (JAMAR) vs Tregs Pain VAS prev week (JAMAR) vs Th1 Pain VAS prev week (JAMAR) vs Th2 Pain VAS prev week (JAMAR) vs IFN-γ Pain VAS prev week (JAMAR) vs IL-6 Pain VAS prev week (JAMAR) vs IL-4 Pain VAS prev week (JAMAR) vs IL-10 Pain VAS prev week (JAMAR) vs TGF-β | No association was observed between pain VAS and Treg, Th1, Th2, IFN-γ, IL-6, or IL-4.  Weak, borderline association: Pain VAS vs IL-10: r = 0.22  Weak association: Pain VAS vs TGF-β: r = 0.33  IL-10 and TGF-β may reflect an anti-inflammatory state and contribute to subjective well-being and reduced pain. | **DISCORDANT Weak; No relationship/association** |
| Radziszewska 2019 | BLOOD BIOMARKERS (OTHER) | Pain VAS (night pain and past week) vs serum cytokines (IL-6, IL-12, IL-17, IL-23, IL-27, TNF-α, IFN-γ) | No correlation found between cytokine levels and pain scores (night or past week)  Well-controlled ERA patients on treatment have similar cytokine profiles as healthy controls and they do not correlate with clinical pain scores or disease activity. | **DISCORDANT No relationship/association** |
| Randell 2024 | MULTI | Pain Interference (PI) vs Active disease (PhGA >1 or AJC >0) PI vs AJC | In the bivariate analysis, pain interference (PI) was higher in children with active disease (PhGA >1 and/or AJC >0). PI increased with the number of active joints (R² = 0.09). However, in multivariable analyses, the magnitude of these relationships was small, and most clinical and demographic variables, including disease activity and treatments, were no longer significant. Functional and psychosocial factors were more strongly related to PI than clinical or demographic variables. | **DISCORDANT Weak/inconsistent** |
| Rapoff 1991 | MULTI | Parent reported pain vs AJC  Parent reported pain vs ESR | Concordant: Pain complaints correlated with AJC (r = 0.46)  No relationship/association: The multiple regression analysis, with parental ratings as predictors of ESR, was not significant. An individual correlation between parental pain complaints and ESR was not reported. The authors noted that the lack of correlation between pain and ESR was expected, as ESR may not accurately reflect disease activity in JRA. ESR can be normal in severe disease or elevated in mild cases, and in this sample the correlation between ESR and active joint count was low (r = 0.29), further questioning ESR’s validity as an indicator of inflammation. | **MIXED** |
| Rashid 2018 | MULTI | Pain VAS vs AJC  Pain VAS vs PhGA | Low pain group showed clear concordance ie low pain, low AJC/PhGA Improved pain: high pain early, then improvement (some early discordance) Consistently high pain showed discordance ie high pain and low-moderate AJC/PhGA AJC and PhGA were not significant predictors of consistently high pain trajectory in multinomial logistic regression. Neither AJC nor PGA at presentation, nor early changes in AJC or PGA, were found to predict membership in either of the three trajectory groups.  Non-inflammatory mechanisms: Disease activity alone cannot fully explain pain; management focused solely on inflammation may not improve pain outcomes Altered pain processing: Potential for peripheral and central sensitization, leading to lowered pain thresholds Patient-reported measures: Strong influence of subjective evaluations (e.g., functional disability, general well-being, baseline pain) over objective disease activity measures (AJC, PhGA) in predicting high pain Older age at onset & Longer disease duration: These factors, possibly linked to diagnostic delays, may magnify pain experience and functional impact  Limited treatment efficacy: Anti-inflammatory medications (DMARDs, biologics) may be less effective at reducing pain in some patients, with biologics nearly trebling the likelihood of persistent high pain Psychosocial and emotional factors: Acknowledged as likely contributors to the magnified experience of pain | **MIXED** |
| Ravelli 1997 | MULTI | Parent pain vs number of swollen joints  Parent pain vs swelling score Parent pain vs number of joints with LROM  Parent pain vs LROM score Parent pain vs AJC Parent pain vs PhGA Parent pain vs ESR Parent pain vs CRP | Parent pain VAS was not strongly correlated with articular variables or lab markers Parent pain vs PhGA r = 0.35 Parent pain vs no swollen joints r = 0.17 Parent pain vs LROM score r = 0.25 Parent pain vs AJC: r = 0.26 Parent pain vs ESR: r = 0.31 Parent pain vs CRP: r = 0.17 Authors stated: an r value >0.7 (bold and underlined) was considered to indicate colinearity  Colinearity is common within categories of disease activity measures (e.g., subjective, functional, articular, laboratory), but uncommon among variables from different categories | **DISCORDANT No relationship/association** |
| Ross 1989 | PhGA | Pain VAS vs: Joint inflammation (physician) Pain VAS vs: Global disease activity (physician) Pain VAS vs morning stiffness (parent/child reported) Pain VAS vs composite of the above (unweighted sum) | Parent pain VAS was significantly associated with disease activity. Correlations with the clinical state composite were r = 0.40 (pauciarticular) and r = 0.56 (polyarticular). Individual indicators (joint inflammation, stiffness, physician global) ranged r = 0.32-0.50. Regression confirmed clinical state was a significant predictor of pain in both subtypes (β = 0.445 and 0.415; p < 0.005).  Reported pain was a valid indicator of clinical state, especially in polyarticular JRA. Contrary to prior assumptions, younger children with pauciarticular disease reported higher pain than older children, and age was not a significant predictor. | **CONCORDANT** |
| Rumsey 2020 | JOINT COUNTS | Pain VAS (prev week, average) vs Entheseal tenderness (presence/absence) Pain VAS (prev week, average) vs Number of affected enthesis sites | Children with enthesitis had worse pain throughout the follow-up compared to those without enthesitis, after adjusting for JIA category and other covariates | **CONCORDANT** |
| Rypdal 2024 | MULTI | At 18 year follow up:  Pain VAS vs AJC Pain VAS vs PhGA | Concordant: At 18-year follow-up, pain VAS was correlated with PhGA (r = 0.61) and AJC (r = 0.56). Median Pain VAS, AJC, and PhGA values were reported for each of the four trajectory groups at 18 years; however, direct correlation analyses between Pain VAS and AJC or PhGA within individual trajectory groups were not presented. Trajectory groups were defined using cJADAS-10 components (PaGA, AJC, PhGA).In the total cohort, PaGA was correlated with pain (r = 0.77) and fatigue (r = 0.76). Discordant (Pain > Disease activity): Group 2 (min –mod disease activity), comprising 24.4% of the cohort, showed a greater increase in PaGA than PhGA over the disease course. At 18-year follow-up, median PaGA was 2.0 (IQR 0.5–4.5) and median PhGA was 1.0 (IQR 0–2.0), with a median AJC of 0 (IQR 0–1). A similar pattern of greater PaGA increase relative to PhGA, without a corresponding increase in active joint count, was reported for patients with active disease in the total cohort. PaGA reflects non-inflammatory factors when high despite low objective disease activity (e.g., AJC, PhGA). This discordance is acknowledged as a “not uncommon clinical experience". Factors contributing to high PaGA include: Functional disability, joint damage (articular and extra-articular; significantly higher JADI-A/E in groups with higher PaGA scores); quality of life issues, pain from sources other than inflammatory disease activity, sleep quality, fatigue When PaGA is the main driver, intensifying traditional DMARDs or biologics may not be the appropriate therapeutic strategy Instead, a multidisciplinary approach is recommended to address the patient's perceived disease burden, incorporating strategies such as: Physical and sport therapy Pain management Coping strategies Family counseling | **MIXED** |
| Schanberg 1997 | PhGA | Oucher vs Physician's disease severity rating Pain VAS vs Physician's disease severity rating Pain location (body map) vs Physician's disease severity rating | Pain thermometer (r = 0.44) Oucher scale (r = 0.50) Pain distribution (r = 0.41)  Current disease activity explained a significant proportion of variance in pain thermometer ratings (28%), Oucher scale ratings (33%), and number of pain locations (13%).  Higher levels of disease activity were related to higher pain ratings and a greater number of reported pain locations. | **CONCORDANT** |
| Schanberg 2003 | MULTI | Pain VAS diary over 2 months vs PhGA Pain VAS diary over 2 months vs AJC Pain VAS diary over 2 months vs ESR Percentage of pain days vs PhGA Percentage of pain days vs AJC | Concordant:  Pain VAS vs PhGA Higher baseline physician global assessment ratings were correlated with increased daily pain (r = 0.39) (PhGA was assessed at baseline, and pain was measured daily over a 2-month period)  Pain VAS vs AJC A higher number of active joints at baseline was correlated with increased daily pain (r = 0.36) (AJC was assessed at baseline, and pain was measured daily over a 2-month period)  Percentage of pain days vs PhGA/AJC The percentage of pain days reported by children classified as having ongoing active disease (85%) was higher than that reported by children with minimal disease activity (58%)  Children with ongoing active disease also reported higher average pain ratings on pain days compared to those with minimal disease activity.  No relationship/association:  Pain VAS vs ESR Daily symptoms, including pain intensity, were unrelated to baseline ESR The lack of relationship between daily symptoms and baseline ESR suggests that ESR might not capture daily fluctuations in symptoms as effectively as other measures.  Reported frequencies of pain and disease flare in the study might be higher than more current rates due to the widespread use of anti-tumor necrosis factor therapy and other biologic agents that became available after study enrollment. | **MIXED** |
| Scott 1977 | MULTI | Pain Pain VAS vs disease activity (ESR + joint swelling) Pain VAS vs disease severity (AJC + functional limitation) | Pain VAS correlated poorly with other measures of disease severity and activity No significant correlation between Pain VAS score and age, activity, or severity scores (r=0.12, 0.2, and 0.2 respectively)  Pain was generally low and not a reliable indicator of disease status in children with JIA. Possibly due to environmental factors, learning/maturation of pain experience, and parental influence. | **DISCORDANT No relationship/association** |
| Sgarlat 2011 | PhGA | Pain NRS vs PhGA | Pain NRS vs PhGA: c = 0.43  The correlation between parent/patient pain (NRS) and Physician Global Assessment (PhGA) (c = 0.43) was considered much less robust and did not correlate strongly, particularly when compared with the highly correlated relationship between parent/patient pain (NRS) and parent/patient global assessment of disease activity (PaGA) (c = 0.75)  Parents or patients may place greater weight on pain when assessing overall disease activity than physicians. | **DISCORDANT Weak/inconsistent** |
| Sherry 1990 | MULTI | Painless vs painful group comparisons: ESR Synovial leukocytes and neutrophils Radiographic damage Synovial biopsies | Concordant: Painlessness showed some concordance with lower inflammatory markers (ESR, synovial fluid neutrophils) Discordant - Pain < Disease activity  However, significant disease activity, including iritis, flexion contractures, and radiographic changes, was present in painless JRA at comparable frequencies to painful JRA Some children without pain also had large numbers of neutrophils in their synovial fluid  Painless JRA showed active disease (e.g., joint damage) but lower synovial fluid inflammatory cells Delayed diagnosis for painless JRA increased risk of untreated iritis/joint damage However, some painless children had significant inflammation/damage, implying painlessness not just absence of disease. Suggest evaluating pain mediators (e.g., prostaglandins, substance P) for further understanding | **MIXED** |
| Shiff 2018 | MULTI | Pain trajectory (derived from VAS) vs Baseline Active Joint Count  Pain trajectory (derived from VAS) vs Baseline ESR   Pain trajectories (derived from VAS) vs Baseline Enthesitis | Concordant: Chronically moderate pain trajectory associated with higher baseline AJC (mean 10.0 vs 2.1 joints for minimal pain) Higher baseline AJC significantly predicted chronically moderate, moderate-decreasing, mild-increasing, and mild-decreasing pain trajectories Discordant - Pain > Disease activity: Many CYP reported frequent/debilitating pain despite effective disease control with therapies (e.g., biologics) Discordant - Pain < Disease activity: Mild-increasing pain trajectory had lower baseline AJC (mean 2.3 vs 5.2 joints) than mild-decreasing pain trajectory, an apparently paradoxical finding Weak/Inconsistent Relationship: Only a small proportion of pain severity variance (13%-33% in children, 2.2% in adults) was explained by physician-rated disease activity No relationship/association: Enthesitis at baseline was not a significant predictor of pain trajectory Baseline ESR was collected but not included in the final multivariable model due to altering trajectory shape  Chronically moderate and mild-increasing pain trajectories may indicate groups with psychosocial comorbidities or poorer coping skills. Factors like previous depression, pain self-efficacy, and pain-coping strategies were not assessed in this study. | **MIXED** |
| Shoop-Worrall 2018 | COMPOSITE | Pain VAS vs cJADAS10 status (CID vs MDA only) | Achieving Clinically Inactive Disease (CID) status on the cJADAS10 was associated with lower Pain Visual Analogue Scale (VAS) scores than achieving Minimal Disease Activity (MDA) alone  At 1 year (cross-sectional), the CID group reported 6.5 mm lower pain scores than the MDA-only reference group Over the longer term (1–5 years), the CID group reported an average of 5.5 mm lower pain scores over 5 years than the MDA-only group  Differences between CID definitions (Wallace vs cJADAS10) appear to be driven by the inclusion of patient/parent well-being in the cJADAS10, which may capture non-inflammatory components such as chronic pain and fatigue that are not assessed by the more objective Wallace criteria. Wallace criteria rely primarily on physician and laboratory measures and may overlook patient-relevant symptoms, indicating that a multifactorial treatment approach may be required when targeting cJADAS10-defined CID  Children who achieved CID using Wallace criteria but not cJADAS10 consistently demonstrated higher disability and poorer psychosocial functioning. | **CONCORDANT** |
| Shoop-Worrall 2021 | MULTI | Baseline Pain VAS vs baseline cluster Pain VAS vs AJC trajectory profile  Pain VAS vs PhGA trajectory profile | Concordant for 78% of the cohort**:** In four of six trajectory groups Low-Remission (32%), Low-Low (20%), High-Low (16%), and High-Low-High (10%): pain, PGE, PhGA, and AJC changed in parallel over time. In these groups, reductions in AJC and PhGA scores were accompanied by improved well-being and reduced pain Discordant – Pain > Disease Activity for 22% of participants  Low-Persistent 14% and High-Persistent 8% trajectory groups showed persistent poor well-being and pain despite low or improving AJC and PhGA. Example values: in the Low-Persistent group, median AJC = 1 (IQR 0–2) and PhGA = 2.8 cm, yet median pain = 5.0 cm and PGE = 5.1 cm; in the High-Persistent group, pain = 5.1 cm and PGE = 4.7 cm despite marked improvement in objective disease activity ie high pain and poor well-being in the absence of ongoing joint inflammation Baseline pain was higher in children who later belonged to both the High-High and High-Persistent groups suggesting that high initial pain predicted higher overall symptom burden but not necessarily later discordance.  Persistent pain despite low or improving disease activity may reflect non-inflammatory or extra-articular pain (e.g. fibromyalgia or enthesitis). These children tended to be older, have longer disease duration, live in more deprived areas, and report poorer HRQoL; anti-rheumatic therapy alone may not address symptoms. | **MIXED** |
| Spadaro 1996 | BLOOD BIOMARKERS (OTHER) | Pain VAS vs IL-6 Pain VAS vs sIL-2R | Concordant: Pain VAS vs IL-6 .487 No relationship/association: Pain VAS vs sIL-2R 0.159 p n.s  IL-6 is significantly correlated with both clinical disease activity and subjective pain severity (VAS), suggesting a role in the inflammatory process and the pain experience in JCA. sIL-2R, while elevated in some patients, did not show a significant correlation with pain or other clinical parameters, suggesting a lesser role. | **MIXED** |
| Spekking 2023 | MULTI | Pain VAS (prev week) vs Inactive disease status (Wallace criteria) Pain VAS (prev week) vs time to inactive disease Pain VAS (prev week) vs Baseline AJC  Pain VAS (prev week) vs Baseline PhGA | Concordant: Overall, pain scores significantly decreased from mean 55.3 mm to 19.5 mm after 24 months of treatment-to-target  Inactive disease status (FU, Wallace criteria) was associated with significantly lower Pain VAS (FU) (β –11.36 mm) The relationship was determined using a model adjusted for baseline confounders, such as JADAS10 .Higher baseline Pain VAS (β 0.46) and higher baseline Active Joint Count (AJC) (β 0.81) were predictive of higher Pain VAS (FU) over time Discordant: 11 percent of patients who achieved clinically inactive disease (ID) at 24 months as defined by the adjusted Wallace criteria, were still experiencing pain classified as moderate (8%)or severe (3%) A longer time to achieve inactive disease (FU) was associated with higher Pain VAS (FU) (β 0.52)  Lower baseline Physician Global Assessment (PhGA) was counterintuitively associated with higher Pain VAS (FU) (β –0.31) in the multivariable model  Some children still experience pain despite achieving clinically inactive disease, emphasizing the need to address patient-reported outcomes in addition to disease activity targets Central sensitization could be a factor in persistent pain even after inflammation is abrogated, and future research should focus on identifying patients at risk for chronic pain For patients with chronic pain despite inactive disease, multifactorial treatment strategies including education about pain mechanisms and psychosocial/physical factors might be beneficial They propose considering an extra patient-reported outcome to investigate if clinical improvement aligns with patient-satisfactory pain levels, suggesting a "dual target strategy" (inflammation control + symptom control) as proposed in adult RA. | **MIXED** |
| Stoll 2010 | IMAGING | Pain (narrative/clinical) vs Sacroiliitis on imaging (MRI/XR) | Sacroiliitis was detected by imaging (MRI or X-ray) in 11/53 (20.8%) children who had no documented history of back pain or stiffness and normal physical examination of the sacroiliac joints at the time of assessment. Physical examination manoeuvres and plain radiography showed limited sensitivity for detecting early sacroiliitis. Early diagnosis often requires advanced imaging, such as MRI, which can detect acute inflammatory changes (e.g., bone marrow oedema) before chronic changes or symptoms develop. The presence of “silent sacroiliitis” indicates that reliance on pain or clinical signs alone may miss axial disease. | **MIXED** |
| Stoustrup 2016 | IMAGING | Pain (TMJ specific questionnaire) vs MRI Pain (TMJ specific questionnaire) vs CBCT | Concordant: 88% of patients with TMJ pain had evidence of TMJ involvement on imaging Discordant - Pain > Disease activity: Pain was also present when imaging was normal Discordant - Pain < Disease activity:  Evidence of TMJ involvement on imaging was present in 79% of patients who reported no TMJ pain. | **MIXED** |
| Sudhakar 2022 | IMAGING | Pain VAS vs MRI BME score | No relationship: No statistically significant correlation between pain VAS and MRI scores (e.g., Pain VAS vs BME: rs = 0.34)  Discordant – Pain < Disease activity: Pain and clinical examination findings (swelling, limitation of range of motion, pain, tenderness) vs MRI were Discordant (Pain < Disease activity) Seventy-six percent of joints with no clinical arthritis still showed early MRI changes  Clinical signs and symptoms underestimate inflammation, particularly in deep joints such as the shoulder. | **DISCORDANT Pain < Disease activity; No relationship/association** |
| Svensson 2000 | IMAGING | Pain at rest and pain on function vs XR | No correlation was found between condylar lesions and TMJ pain. Radiographic condylar changes were significantly associated with stiffness at mouth opening, but not with pain.  Pain reporting may be influenced by lower medication use in this sample and by the subjective, variable nature of pain. Authors note that children tend to report arthritis-related pain less consistently than adults. | **DISCORDANT Pain < Disease activity** |
| Svensson 2001 | IMAGING | TMJ pain self report + pain on palp vs OPG and histological findings of condylar destruction and pathological changes observed at surgery | Structural damage was extensive, but pain minimal Pain not a reliable indicator of TMJ damage  TMJ destruction may occur without symptoms due to chronicity or treatment with NSAIDs/steroids Lower TMJ compartments were consistently affected, indicating a polarised pattern of pathology Structural damage of the TMJ does not necessarily result in pain, consistent with findings in adult RA. | **DISCORDANT Pain < Disease activity** |
| Tarakci 2011 | JOINT COUNTS | Pain VAS vs AJC | Significant but weak positive correlation between Pain VAS and number of active joints r = 0.30 | **DISCORDANT Weak/inconsistent** |
| Tarkiainen 2018 | COMPOSITE | Pain VAS vs JADAS Pain VAS vs Cumulative time spent in ID | JADAS at each time point associated with level of pain Cumulative time spent in ID associated with lower level of pain at 3 years (coefficient beta-0.63) Pain decreased as disease activity diminished | **CONCORDANT** |
| Thastum 2011 | CLINICAL EXAMINATION + LABORATORY PARAMETERS (defined composite) | FPS vs disease activity score | Pain level did not change despite decreased disease activity over time. Disease activity explained only a modest proportion of pain variance. A subgroup had high pain despite low disease activity, which was consistent over time. At T2 correlation between pain report and disease activity was .39  Pain-specific beliefs (e.g., harm and disability beliefs) were significantly higher in the high-pain/low-disease group and predicted later pain, suggesting they contribute to discordance between pain and disease activity. Pain-specific beliefs may be more influential on pain than general emotions. | **DISCORDANT Pain > Disease activity** |
| Thompson 1987 | PhGA | Pain VAS (intensity) vs Disease Activity Index | More intense present pain ratings were associated with more active disease. A significant correlation was found for each rater: child (r = 0.35), mother (r = 0.64), and physician (r = 0.65). Child-rated present pain vs disease activity index: r = 0.35 | **CONCORDANT** |
| Tirelli 2020 | COMPOSITE | Pain VAS vs cJADAS | Pain VAS vs cJADAS Higher pain was associated with higher cJADAS (β = 0.76)  cJADAS vs Pain VAS Higher cJADAS was associated with higher pain (β = 0.31)  Time effect: cJADAS showed significant improvement over time (β = −0.03 to −0.07 per month) Pain showed no meaningful change over time  Incident ERA cases had a greater rate of cJADAS improvement over time, but pain persisted over time among both incident and prevalent cases Pain persisted despite improvements in disease activity. Baseline pain strongly predicted higher pain at follow-up. Pain may indicate ongoing inflammation requiring more aggressive therapy or reflect residual symptoms requiring non-pharmacologic management. | **MIXED** |
| Trachtman 2021 (JCR) | COMPOSITE | CYP reported data (≥10 yrs) n =29 Pain Interference vs JADAS-71 Pain Interference vs JADAS-71 without patient global   Parent Proxy Reported Data (Children 1–9 Years) n = 14 Pain Interference vs JADAS-71 Pain Interference vs JADAS-71 without parent global | Patient self-reported Pain Interference (PI) vs JADAS (n = 29) PI T-score vs full JADAS-71: r = 0.55 PI T-score vs JADAS without patient global (PG): r = 0.41  Parent proxy-reported Pain Interference (PI) vs JADAS (n = 14) PI T-score vs full JADAS-71: r = 0.34 PI T-score vs JADAS without PG: r = 0.28  Any significant correlations were driven by the patient global (PG) component, which is subjective. Removal of PG weakened nearly all correlations. JADAS-71 may be inadequate for assessing psychosocial domains such as Pain Interference. Parent proxy JADAS responses correlated extremely poorly with PROMIS CAT measures. JADAS-71 was not recommended for use with parent proxy responses. | **MIXED** |
| Trachtman 2021 (JPRO) | PhGA | Pain VAS (prev week) vs PhGA | Discordant Pain > Disease activity: Prior to flare, pain scores increased at the visit immediately before flare, while PhGA scores did not show a similar increase. For the 42 subjects with a visit immediately prior to flare, the mean PhGA score was 0 (SD 0.1), whereas the mean pain score was 0.4 (SD 1.0).  Concordant: At the time of flare, PhGA scores were moderately correlated with pain scores (r = 0.45).  Overall, the predictive value for flare showed a weak and inconsistent relationship for both PhGA and pain, with low discrimination (AUC 0.51–0.58). Simple patient-reported outcomes, such as pain VAS, capture important aspects of disease that may not be reflected in provider-based measures of overall disease activity (PhGA). The weak predictive performance suggests that more sophisticated PROs may be required. | **MIXED** |
| Tripalo Batoš 2020 | IMAGING | Pain VAS vs indices of flexion, extension and sagittal flexibility Pain VAS vs MRI findings (cervical spine, SIJ, thoracic, lumbar spine) Pain VAS vs X-ray findings (cervical spine) | Statistical analysis:  No relationship ie Pain VAS was not statistically significantly correlated with clinical indices or imaging findings Narrative : Pain < Disease activity - pain is low despite active disease (clinical or imaging evidence) Authors note that pain was not the leading clinical sign, even in patients with positive cervical MRI findings  Pain may not be the leading clinical sign in JSpA with axial involvement, especially when pathological changes are detected on cervical MRI The small number of patients was a study limitation Children were in an early stage of the disease | **DISCORDANT Pain < Disease activity; No relationship/association** |
| Tucker 2009 | MULTI | Pain (VAS) vs AJC Pain (VAS) vs PhGA Decrease in pain vs decrease in active joints Decrease in pain vs ESR at 6 months | Strong relationship, supported both statistically and narratively Active joint count was an independent predictor of pain at baseline and at 6 months Reductions in active joint count and ESR at 6 months predicted reductions in pain Decreases in ESR and active joint count were associated with reduced pain Physician and patient/parent global assessments were strongly associated with pain Objective measures of disease activity were the strongest predictors of pain, while demographic and socioeconomic variables did not influence pain in this cohort. | **CONCORDANT** |
| Tupper 2013 | PhGA | Pain variability via diary vs PGADS | Disease severity (PGADS) predicted a higher probability of greater pain variability. For every 10-unit increase in PGADS, the odds of higher pain variability increased by approximately 25%.  Pain variability represents an important source of heterogeneity and should be considered in research design, including controlling for time of day when using recalled pain measures. Daily fluctuations in pain intensity are common and can be anticipated. | **CONCORDANT** |
| Ünal 2018 | COMPOSITE | Pain VAS from CHAQ vs JADAS | Pain CHAQ vs JADAS r=0.499 | **CONCORDANT** |
| Upadhyay 2021 | MULTI | Pain intensity (NRS) vs ESR Pain reports/symptomatology (NRS pain intensity, pain behavior, pain quality) vs MSK MRI Pain intensity (NRS) vs cJADAS | No relationship/association No significant group-level correlation between pain severity and systemic inflammatory markers (ESR, CRP) or composite disease activity scores (cJADAS) (R = -0.12 to 0.49) Individual patient vignettes illustrate both concordance (pain matching elevated markers/pathology) and discordance (pain present despite normal markers/no pathology, or low pain despite active disease) Prevalent disconnect between subjective pain and objective measures of inflammation/pathology in JIA  Pain in JIA can persist independently of inflammation, suggesting it's underpinned by altered brain structure and functional neurocircuitry. Neuroimaging findings demonstrate distinct neurobiological mechanisms of pain (including affective and sensory qualities) beyond peripheral disease activity, which helps explain the observed disconnect between subjective pain and objective inflammation. Important to understanding and addressing JIA pain directly, rather than solely focusing on inflammatory markers. | **MIXED** |
| Van Der Heijden 2022 | BLOOD BIOMARKERS (OTHER) | Pain severity (PROMIS Numeric Rating Scale) vs serum protein level | Discordant: discordance was observed in both directions; no consistent alignment between pain and disease activity Concordant: several serum proteins were significantly associated with pain severity (e.g., PRG4, CST3, IGKV3D.15)  The authors identified 14 proteins significantly associated with pain severity in JIA, including immune and inflammatory proteins such as PRG4, CST3, and VWF. Some of these proteins also correlated with ESR, while others did not, suggesting both inflammatory and non-inflammatory mechanisms of pain. These results indicate that current clinical measures of disease activity do not fully explain pain in JIA, and that serum proteomic markers may offer new insight into both peripheral and central mechanisms underlying pain. | **MIXED** |
| van Dijkhuizen 2023 | MULTI | Pain VAS vs Physician Global Assessment (PhGA)  Pain VAS vs Number of Joints with Active Arthritis (AJC/NAJ) Pain VAS vs Swollen Joint Count (SJC) Pain VAS vs Tender Joint Count (TJC) Pain VAS vs. Number of Joints with Limitation on Motion (NLJ) Pain VAS vs Erythrocyte Sedimentation Rate (ESR) Pain VAS vs cJADAS10 | Concordant (moderate to strong) Spearman ρ:  Parent-reported Pain VAS (previous week) vs: cJADAS10: 0.71 PhGA: 0.57 TJC: 0.55 AJC: 0.45  Patient-reported Pain VAS (previous week) vs: cJADAS10: 0.70 PhGA: 0.56 TJC: 0.54 AJC: 0.43  Weak / inconsistent  Parent-reported Pain VAS (previous week) vs: SJC: 0.39 LROMC: 0.35 ESR: 0.22  Patient-reported Pain VAS (previous week) vs: SJC: 0.37 LROMC: 0.33 ESR: 0.20 | **MIXED** |
| Vandvik 1990 | PhGA | Present pain VAS vs PhGA Worst pain VAS vs PhGA | In the total sample, no significant correlation was found between the child's present pain and disease severity rated by the physician (r=0.06). Similarly, no correlation was found between the child's worst pain and the physician's disease severity rating (r=-0.06) An exception to this lack of correlation was noted in the small observation group (n=10), where the physician's rating of disease severity showed a tendency to be related to the child's rating of pain (r=0.55, p=0.06)  Lack of correlation between the child's pain and the physician's disease severity assessment may be due to methodological differences. In this study, physician assessments were based on routine medical records and were not explicitly made in the context of the child's pain reports. Patients and parents may base their assessment of disease severity more on pain and functional disability compared to physician. | **DISCORDANT No relationship/association** |
| Varni 1987 | PhGA | Child, Parent Pain VAS intensity (present pain) vs Physician-rated Disease Activity Index | As physician-rated disease activity increased, there was a corresponding increase in rated present pain intensity by the child, parent, and physician | **CONCORDANT** |
| Vega-Fernandez 2023 | IMAGING | Pain VAS vs US | At baseline, Pain VAS showed a weak association with MSUS synovitis (rs = 0.27–0.28 across joint sets). At follow-up, Pain VAS showed no association with MSUS synovitis (rs = 0.02–0.11). Overall, pain ratings did not align with ultrasound-detected synovitis at either time point. Cognitive, emotional, and situational factors may exaggerate or minimise self-reported ratings of pain and well-being. | **DISCORDANT No relationship/association** |
| Verstappen 2015 | MULTI | Pain VAS vs AJC, LROMC PhGA, ESR, JADAS-71, JADAS3-71 | Pain was higher in the low-SES group, despite no differences in clinical disease activity measures (AJC, PhGA, JADAS71) between SES groups. This discordance may reflect unmeasured factors such as comorbidities or social stressors, indicating that clinical scores alone do not capture the full disease burden. Greater multidisciplinary support may be needed for children with higher self-reported symptoms. | **MIXED Pain > Disease activity** |
| Viswanath 2011 | BLOOD BIOMARKERS (OTHER) | Patient Pain VAS vs MMP-3 | MMP-3 vs Pain VAS: r = 0.28 | **DISCORDANT Weak/inconsistent** |
| von Schuckmann 2020 | IMAGING | Pain at rest vs MRI  Pain while chewing vs MRI | Concordant: Pain while chewing was statistically significantly associated with MRI-confirmed TMJ involvement No relationship/association: Pain at rest was not statistically significantly associated with MRI-confirmed TMJ involvement  While pain often indicates TMJ involvement (high PPV), the absence of pain does not rule it out (low NPV) because many TMJ involvement cases are asymptomatic. | **MIXED** |
| Vuorimaa 2008 | JOINT COUNTS | Pain frequency vs AJC | Concordant patterns:  Cluster 1: High pain co-occurred with high disease activity (high active joint counts)  Discordant patterns:  , Cluster 4: High pain despite low disease activity, characterised by low active joint counts and good functional status  Cluster 3: Low pain despite relatively high disease activity, with higher active joint counts but minimal pain and disability  Cluster 2: Moderate to high pain with low disease activity and short disease duration, indicating pain exceeding objective disease markers  Overall, clusters demonstrated both Pain > Disease Activity and Pain < Disease Activity patterns indicating that pain and disease activity did not align consistently across patient subgroups. | **MIXED** |
| Weiss 2012 | MULTI | Pain intensity vs AJC Pain intensity vs PhGA Pain intensity vs XR Pain intensity vs Enthesitis (specifically in ERA patients) Pain intensity vs Sacroiliac tenderness (specifically in ERA patients) | Concordant: Pain intensity vs AJC (full JIA cohort): Higher active joint count was significantly and independently associated with higher pain intensity Pain intensity vs enthesitis (ERA): Presence of active enthesitis was significantly and independently associated with higher pain intensity Pain intensity vs sacroiliac tenderness (ERA): Presence of sacroiliac tenderness was significantly and independently associated with higher pain intensity  Discordant – Pain > Disease activity: Pain intensity vs Physician Global Assessment (PGA): The correlation between PGA and pain was low (r = 0.43). Many participants reported pain despite low PGA scores  Weak / inconsistent: Pain intensity vs AJC (ERA subgroup): Active joint count showed a significant univariate association with pain but was not retained in the final multivariable model for ERA  No relationship / association: Pain intensity vs radiographic joint damage (X-ray): No statistically significant association was observed between radiographic evidence of joint damage and pain intensity in either the full JIA cohort or the ERA subgroup.  Pain intensity was only partly explained by clinical disease measures; psychosocial factors such as stress, mood, coping, and illness perceptions were noted as likely contributors but were not directly measured. | **MIXED** |
| Weiss 2016 | IMAGING | Back pain (narrative/clinical) (current or history) vs MRI sacroiliitis Inflammatory back pain (narrative/clinical) vs MRI sacroiliitis | Discordant (Pain < Disease activity): Active sacroiliitis, as identified by MRI, was frequently asymptomatic Of the 8 subjects with active sacroiliitis on MRI, 5 (62%) did not report current or a history of back pain Only 2 out of 8 (25%) of those with MRI-confirmed active inflammatory sacroiliitis reported inflammatory back pain While 38% of MRI-positive patients did report back pain, the majority (62%) did not Weak/inconsistent & No Relationship/Association: There was no significant difference in the prevalence of general back pain (38% in MRI-positive vs 59% in MRI-negative) or inflammatory back pain (25% in MRI-positive vs 22% in MRI-negative) between children with and without MRI findings of sacroiliitis For inflammatory back pain, the positive predictive value (PPV) was low (0.22) and its negative predictive value (NPV) was 0.81 The sensitivity of inflammatory back pain for sacroiliitis was low (0.25)  Juvenile SpA differs from adult SpA: Active sacroiliitis is frequently asymptomatic in children; inflammatory back pain is less common.Adult criteria are inadequate for children: The adult definition of axial SpA, which relies on inflammatory back pain, performs poorly in juvenile SpA due to low sensitivity for sacroiliitis. Peripheral symptoms are more prominent in children: Peripheral arthritis and enthesitis are often the most important early manifestations in children, not inflammatory back pain. | **DISCORDANT Pain < Disease activity; No relationship/association** |
| Xu 2016 | BLOOD BIOMARKERS (OTHER) | Pain VAS vs cytokines | IL-17 positively correlated with VAS (r = 0.524, p = 0.003); IL-6 not significantly correlated with VAS | **MIXED** |
| Yamazaki 2022 | IMAGING | Inflammatory back pain (narrative/clinical)vs MRI | The clinical severity of inflammatory back pain and peripheral enthesitis did not correlate with the MRI findings  The ASAS definition for adults might be less sensitive in children, and that inflammatory changes in the sacroiliac joint can be subtle in the early stages of sacroiliitis, potentially not being detected on MRI. | **DISCORDANT No relationship/association** |
| Yilmaz 2010 | IMAGING | Pain (narrative/clinical) vs XR Pain (narrative/clinical) vs MRI | Concordant with MRI Discordant with XR Authors highlight that MRI revealed sacroiliitis and enthesitis in all patients, even though radiographs were normal.  Plain film findings of the pelvis were within normal limits in all patients. This means that despite the extensive inflammatory changes visible on MRI, standard X-rays showed no signs of pathology like erosions, sclerosis, subchondral cysts, fissures, or bony ankylosis. MRI is now accepted as the radiological modality of choice, especially in children and adolescents, for evaluating pathological changes in the sacroiliac joints. They emphasize that MRI allows detailed evaluation of inflammatory and destructive changes in the pelvis in juvenile-onset ankylosing spondylitis (JAS) patients | **MIXED** |
| Zhang 2025 | MULTI | Pain VRS vs IL-10 Pain VRS vs IL-6  Pain VRS vs TNF-α  Pain VRS vs ESR  Pain VRS vs CRP Pain VRS vs Procalcitonin (PCT) Pain VRS vs clinical examination  Pain VRS vs Joint locations of pain | Concordant (C1): Clinical signs of inflammation (joint swelling, warmth) aligned with moderate-to-severe pain and functional impairment  Discordant – Pain < Inflammation (C3): Systemic inflammation with little or no reported joint pain ESR and CRP did not differ across pain phenotypes, showing no association with pain classification  Phenotype-specific findings: IL-10 levels were higher in C2 (mild intermittent pain with extra-articular symptoms) than in C1 Higher IL-10 was associated with milder pain phenotypes Younger age was associated with the no–joint-pain phenotype (C3)  Pain phenotypes differed in short-term clinical outcomes, including discharge pain status, internal transfers, and hospital length of stay. | **MIXED** |
| Zwir 2012 | IMAGING | Pain (narrative/clinical) vs MRI | Most children were asymptomatic despite abnormal MRI findings  TMJ should always be evaluated in JIA patients even in the absence of signs and symptoms because condylar damage can occur early and progress silently. Caution needed in interpreting synovial enhancement, as it can be present even in patients in remission. | **DISCORDANT Pain < Disease activity** |

**Marker categories reflect the type of objective or clinical disease activity measure analysed in relation to pain. PhGA refers to physician global assessment alone. Joint counts include active, swollen, tender, limited range of motion, or enthesitis counts analysed as individual clinical measures. Composite refers to validated composite disease activity scores that include both objective clinical components and a subjective patient- or parent-reported component (e.g., global assessment or well-being), such as the Juvenile Arthritis Disease Activity Score (JADAS) and variants. Wallace refers to ACR provisional criteria for clinically inactive disease. Imaging includes ultrasound, MRI, CBCT, CT, or radiography. Blood biomarkers (other) refers to laboratory markers other than ESR or CRP (e.g., cytokines, proteomic or molecular markers) analysed independently of any composite score. Clinical examination (undefined) refers to clinician-assessed disease activity where specific components were not clearly specified. Clinical examination + laboratory parameters (defined composite) refers to author-defined composite measures combining clinical examination findings with laboratory parameters. Thermography refers to temperature-based imaging measures.*

**This table is descriptive and maps how included studies analysed pain–disease activity marker relationships. Correlation coefficients are shown as reported to document the basis for concordance category assignment; no quantitative synthesis (e.g., meta-analysis) was undertaken, consistent with a scoping review.

*ACR = American College of Rheumatology ACRCID = ACR provisional criteria for Clinical Inactive Disease AD = active disease AIMS = Arthritis Impact Measurement Scales AJC = active joint count ASAS = Assessment of SpondyloArthritis international Society AUC = area under the curve bDMARD = biologic disease-modifying antirheumatic drug BME = bone marrow oedema cA2 = chimeric anti–tumour necrosis factor-α monoclonal antibody (infliximab) CBCT = cone-beam computed tomography CDAI = Clinical Disease Activity Index CHAQ/C-HAQ = (Childhood) Health Assessment Questionnaire CHU9D = Child Health Utility 9D CID = clinically inactive disease cJADAS = clinical Juvenile Arthritis Disease Activity Score cm = centimetre CRP = C-reactive protein CST3 = cystatin C CT = computed tomography CYP = children and young people DAS = Disease Activity Score DAS28 = Disease Activity Score (28-joint) DMARD = disease-modifying antirheumatic drug dT = temperature difference (thermography) ERA = enthesitis-related arthritis ES = effect size ESR = erythrocyte sedimentation rate FGF21 = fibroblast growth factor 21 FPS/FPS-R = Faces Pain Scale/Faces Pain Scale–Revised FU = follow-up GDNF = glial cell line–derived neurotrophic factor HRQoL = health-related quality of life ID = inactive disease IFN-γ = interferon gamma IGKV3D.15 = immunoglobulin kappa variable 3D-15 IL = interleukin IL-1ra = interleukin-1 receptor antagonist IL-18R1 = interleukin-18 receptor 1 IQR = interquartile range JA = juvenile arthritis JADAS = Juvenile Arthritis Disease Activity Score JADI-A = Juvenile Arthritis Damage Index–Articular JADI-E = Juvenile Arthritis Damage Index–Extra-articular JAMAR = Juvenile Arthritis Multidimensional Assessment Report JAS = juvenile-onset ankylosing spondylitis JCA = juvenile chronic arthritis JE = joint effusion JIA = juvenile idiopathic arthritis JRA = juvenile rheumatoid arthritis JSpA = juvenile spondyloarthritis LDA = low disease activity LP = lumbar pain LROM/LROMC = limited range of motion/limited range of motion count MAD = minimally active disease MCP = metacarpophalangeal (joint) MDA = minimal disease activity ME/MV/REE = dynamic MRI parameters MIO = maximal interincisal opening mm = millimetre MMP-3 = matrix metalloproteinase-3 MRI = magnetic resonance imaging MRP8/14 = myeloid-related protein 8/14 (calprotectin) MSK = musculoskeletal MSUS = musculoskeletal ultrasound NA = not applicable NAD = non-active disease NLJ = number of joints with limitation NPV = negative predictive value NRS = numerical rating scale NS = not significant OPG = orthopantomogram (panoramic radiograph) PaGA = patient/parent global assessment PCT = procalcitonin PD = power Doppler PGA = patient global assessment or physician global assessment PGADS = physician global assessment of disease severity PGE = patient global evaluation PI = pain interference PIP = proximal interphalangeal (joint) POM/POMC = pain on motion/pain on motion count PPQ = Pediatric Pain Questionnaire PPV = positive predictive value PRG4 = proteoglycan 4 (lubricin) PROMIS = Patient-Reported Outcomes Measurement Information System PROMIS CAT = PROMIS Computer Adaptive Test RA = rheumatoid arthritis RF = rheumatoid factor SAA = serum amyloid A SDAI = Simplified Disease Activity Index SES = socioeconomic status SIJ = sacroiliac joint sIL-2R = soluble interleukin-2 receptor SIP = sacroiliac pain SIS = SPARCC Inflammation Score SJC = swollen joint count SPARCC = Spondyloarthritis Research Consortium of Canada SPJC = self-reported painful joint count SRM = standardised response mean sTNFR = soluble tumour necrosis factor receptor TEN/POMC = tenderness/pain on movement count TGF-β = transforming growth factor beta Th1/Th2/Th17 = T helper 1/2/17 cells TJC = tender joint count TMJ = temporomandibular joint TNF-α = tumour necrosis factor alpha Treg(s) = regulatory T cell(s) US/USG = ultrasound/ultrasonography VAS = visual analogue scale VEGFA = vascular endothelial growth factor A VRS = verbal rating scale VWF = von Willebrand factor XR = X-ray*

**Table S3.** Composite disease activity scores (e.g., JADAS, cJADAS)

| First author/Year | Design | Sample | Specific comparison | Timepoint | Analysis | Relationship | Summary of author findings |
| --- | --- | --- | --- | --- | --- | --- | --- |
| Brandon 2017 | Cross-sectional | 265 | PI vs JADAS3 | Concurrent | Mann-Whitney test | **Concordant** | Pain interference discriminated between active and inactive disease for both child and parent reports. |
| Dekker 2010 | Cross-sectional | 34 | Pain VAS overall vs JADAS71 Pain VAS feet vs JADAS71 | Concurrent | Spearman correlation | **Concordant** | VAS overall body pain vs JADAS71 r=0.8 VAS pain feet vs JADAS71 r=0.7. |
| Esbersen 2018 | Prospective observational | 93 | Pain VAS vs JADAS-27 | Concurrent | Not reported | **Concordant** | JADAS-27 was significantly positively correlated to pain intensity |
| Khanom 2022 | Cross-sectional analysis of secondary data (CAPS) | 97 | Pain CHU9D vs cJADAS71 | Concurrent (within 2 months) | Spearman correlation; Hierarchical linear regression | **Mixed** Limited explanatory power | Moderate pain–cJADAS association (r = 0.39; β = 0.34), but disease activity explained only 15% of pain variance, suggesting anti-inflammatory treatment alone may be insufficient. |
| Kovalchuk 2018 | Cross-sectional | 60 | Child Pain VAS vs DAS/DAS28 Child Pain VAS vs SDAI/ CDAI Parent Pain VAS vs DAS/DAS28 Parent Pain VAS vs SDAI/CDAI | Concurrent | Spearman correlation | **Concordant** | Moderate correlations between pain and disease activity measures (ρ = 0.48–0.62) |
| Mannion 2022 | Registry based (CARRA) | 6,235 | PI vs cJADAS ID PI vs cJADAS LDA | JADAS at BL, PI at 6 months FU | Descriptive stats; relative change based on MCID | **Mixed** Limited explanatory power | Although cJADAS ID and cJADAS were associated with relative change in pain interference, similar worsening frequency of PI across all groups at FU (1/3 PI improved, 1/3 PI worse, 1/3 PI unchanged). |
| Min 2020 | Registry establishment | 112 | Pain VAS vs cJADAS | Concurrent | Spearman correlation; Kruskal-Wallis; Descriptive stats | **Concordant** | Pain VAS demonstrated a positive correlation with disease activity (*R*=0.58). |
| Miyamae 2019 | Case series report | 3 | Pain (narrative/clinical) vs DAS28-ESR | Concurrent | Narrative only | **Discordant** Pain < Disease activity | Active disease without pain, potentially due to neurodevelopmental differences in pain perception and reporting in early childhood, leading to delayed diagnosis. |
| Mountain 2025 | Cross-sectional using CAPS data | 187 | Pain VAS vs cJADAS | Concurrent | Spearman correlation; Descriptive stats | **Mixed**  Subgroup divergence | Pain showed a moderate–strong association with cJADAS-10 (ρ = 0.51), with both concordant and discordant pain–disease subgroups. High pain severity was associated with threat-based beliefs and low perceived control irrespective of disease activity. |
| Nguyen 2024 | Prospective inception cohort comparison using registry data (ReACCh-Out 2005-2010 vs CAPRI 2017-2021) | 1849 total ReACCh-Out: 1128 CAPRI: 721 | Parent pain VAS/NRS prev week vs. cJADAS10 ID Parent pain VAS/NRS prev week vs. cJADAS10 mDA | Longitudinal | Kaplan–Meier survival analysis; Multivariable Cox proportional hazards models | **Mixed** Time/outcome divergence | cJADAS10 attainment improved more than pain over 70 weeks (ID 50-69%; mDA 69-84% vs Pain <1 57-63%). Pain and global improvement lagged disease activity. Differences attributed to measurement effects, expectations, and psychosocial factors, with physician–patient/parent discordance. |
| Shoop-Worrall 2018 | Longitudinal inception cohort analysis (CAPS) | 832 | Pain VAS vs cJADAS10 status (CID vs mDA only) | Concurrent | Multilevel, multivariable regression Cross-sectionally at 1 year and annually up to 5 years following initial presentation | **Concordant** | CID defined by cJADAS10 was associated with lower pain than mDA (≈6 mm at 1 year; ≈5.5 mm over 5 years). Inclusion of patient/parent well-being captures non-inflammatory symptoms not reflected in Wallace criteria. Children meeting CID by Wallace alone showed greater disability and poorer psychosocial function. |
| Tarkiainen 2018 | Part of RCT | 60 | Pain VAS vs JADAS Pain VAS vs Cumulative time spent in ID | Concurrent | Not reported | **Concordant** | Pain decreased as disease activity improved, with JADAS and pain aligned at each time point. More time in inactive disease was linked with lower pain at 3 years. |
| Thompson 1987 | Cross-sectional validation | 23 | Pain VAS vs Disease Activity Index | Concurrent | Pearson correlation Welch’s V statistic | **Concordant** | More intense present pain ratings were associated with more active disease. A significant correlation was found for each rater: child, r = .35, mother, r = .64 and physician, r = .65. |
| Tirelli 2020 | Quasi-experimental pre–post cohort (retrospective data analysis, ReACCh-Out cohort) | 152 | Pain VAS vs cJADAS | Concurrent | Repeated-measures linear mixed-effects models (AR1 covariance) | **Mixed** Time/outcome divergence | Higher pain was associated with higher cJADAS (β 0.76), and higher cJADAS with higher pain (β 0.31). cJADAS improved over time (β –0.03 to –0.07/month), but pain did not. Incident ERA showed greater cJADAS improvement with persistent pain. Higher baseline pain predicted higher follow-up pain, consistent with residual inflammation or non-inflammatory contributors requiring non-pharmacological management. |
| Trachtman 2021 | Cross-sectional | 44 | CYP reported data (≥10 yrs) PI vs JADAS-71 PI vs JADAS-71 without patient global Parent Proxy Reported Data (Children 1–9 Years)  PI vs JADAS-71 PI vs JADAS-71 without parent global | Concurrent | Spearman correlation | **Mixed** Composite score dependency (relationship driven by subjective component) | Only the full JADAS-71 (with patient global) showed a moderate correlation with PI (r≈0.55). Removing the patient global reduced correlations to weak levels (r≈0.28–0.41), indicating the relationship was driven by the subjective patient global component. |
| Ünal 2018 | Cross-sectional | 196 | Pain VAS from CHAQ vs JADAS | Concurrent | Not reported | **Concordant** | Pain CHAQ vs JADAS r=0.499 |
| Upadhyay 2021 | Pilot observational study with multidisciplinary and neuroimaging assessments. Four clinical vignettes. | 16 | Pain intensity (NRS) vs cJADAS | Concurrent | Two-tailed t-tests (low vs high pain groups); Spearman correlations | **Discordant** | No meaningful relationship was observed between pain severity and ESR, CRP, or cJADAS, indicating frequent discordance between subjective pain and objective inflammatory activity in JIA. Pain appeared independent of inflammation and was linked to altered brain structure and neurocircuitry, supporting pain as a distinct construct beyond disease activity. |
| van Dijkhuizen 2023 | Validation | 6060 (8643 parents) | Pain VAS vs cJADAS10 | Concurrent; Longitudinal Concurrent (correlations at same clinic visit) Test–retest reliability (Pain VAS, 7–14 days apart) | Spearman correlations (validity); ICC (reliability); Bland–Altman analysis (agreement) | **Concordant** | Parent-reported Pain VAS Prev week vs:  cJADAS10: 0.71   Patient-reported Pain VAS prev week vs:  cJADAS10: 0.70 |
| Verstappen 2015 | Secondary analysis of RCT or cohort study Cross-sectional analysis at baseline | 923 | Pain VAS vs JADAS71, JADAS371 | Concurrent | Descriptive stats; ZINB, linear, median, logistic, and multinomial logistic regression. | **Mixed** Subgroup divergence (lower SES) | Pain higher in lower-SES groups despite no differences in clinical disease activity. This discordance may reflect unmeasured factors such as comorbidities or social stressors. Clinical measures did not capture full disease burden, indicating a need for greater multidisciplinary support in children with higher self-reported symptoms. |

Concordance categories in this table are assigned at the marker level. Individual studies may therefore appear as concordant for a given marker despite being classified as mixed at the study level when multiple markers were considered.

*AR1 = autoregressive order 1 BL = baseline CAPRI = Canadian Alliance of Pediatric Rheumatology Investigators CAPS = Childhood Arthritis Prospective Study CARRA = Childhood Arthritis and Rheumatology Research Alliance CHAQ = Childhood Health Assessment Questionnaire CHU9D = Child Health Utility 9D CID = clinically inactive disease cJADAS = clinical Juvenile Arthritis Disease Activity Score*  *cJADAS10 = clinical Juvenile Arthritis Disease Activity Score (10-joint version)*   *DAS = Disease Activity Score DAS28 = Disease Activity Score in 28 joints ERA = enthesitis-related arthritis ESR = erythrocyte sedimentation rate FU = follow-up ICC = intraclass correlation coefficient ID = inactive disease JADAS = Juvenile Arthritis Disease Activity Score JADAS-71 = Juvenile Arthritis Disease Activity Score (71-joint version)  LDA = low disease activity  MCID = minimal clinically important difference mDA = minimal disease activity NRS = numeric rating scale PI = pain interference PRO = patient-reported outcome RCT = randomised controlled trial 
SES = socioeconomic status SDAI = Simplified Disease Activity Index VAS = visual analogue scale ZINB = zero-inflated negative binomial*

**Table S4.** Physician global assessment (PhGA)

| First author/Year | Design | Sample | Specific comparison | Time-point | Analysis | Relationship | Summary of author findings |
| --- | --- | --- | --- | --- | --- | --- | --- |
| Abu-Saad 1995 | Cross-sectional | 33 | Pain VAS (worst prev week) vs Physician global disease index Pain VAS present vs Physician global disease index | Concurrent | Spearman correlation  Mann-Whitney U | **Mixed** Pain-measure recall period divergence | Worst pain (past week) correlated with PhGA, whereas present pain did not; present pain may underestimate disease impact. |
| Armbrust 2013 | Cross-sectional (Nordic JIA cohort) | 113 | Pain VAS vs PhGA | Concurrent | Narrative | **Mixed** Subgroup divergence | **Concordant:** Low pain with no active disease; high pain with active disease. **Discordant:** High pain overestimated disease and did not distinguish active from inactive disease; pain may be unrelated to activity and reflect biological or emotional factors requiring education rather than treatment adjustment. |
| Kovalchuk 2018 | Cross-sectional | 60 | Child Pain VAS vs PhGA Parent Pain VAS vs PhGA | Concurrent | Spearman correlation | **Concordant** | Child Pain VAS vs PhGA: 0.69 Parent Pain VAS vs PhGA: 0.57 |
| Learoyd 2019 | Retrospective, Longitudinal, Observational Cohort Study | 97 | At individual clinic visits: Pain VAS (present/current) vs PhGA   Across trajectories: Pain trajectory vs Baseline PhGA | Multiple time points (3 visits over 50 weeks) Concurrent (same-visit correlations) | Linear regression One-way ANOVA Multinomial logistic regression Chi-Square test Fisher's Exact test | **Concordant** | Single clinic visit pain associated with PhGA. Higher average PGA at study onset in variable/high pain trajectories vs low pain. |
| Malleson 2004 | Secondary analysis of RCT or cohort study | 388 | Pain VAS (prev week, worst) vs PhGA | Concurrent | Spearman's rank correlation Kruskal-Wallis tests Stepwise linear regression analyses | **Discordant** | Disease-related factors explained little pain variance. In multivariate models, PhGA accounted for only 6.5% of pain variation (5.3% for scores 2–3; 1.2% for score 1). Differences from other studies may reflect use of present vs worst pain. Pain in JRA is multifaceted and not explained by disease or demographics alone, highlighting the role of coping, mood, and family factors. |
| Mannion 2019 | Secondary analysis of cohort study (CARRA Registry) | 2164 | Pain score vs PhGA | Concurrent | Pearson correlation | **Discordant** | Weak correlation between PhGA and pain score (R=0.24) |
| Miller 2011 | Cross-sectional, retrospective | 160 | Pain NRS (prev week, average) vs PhGA | Concurrent | Spearman correlation | **Concordant** | ERA: strong 0.89 Systemic: strong 0.76 RF- poly: mod 0.55 RF+ poly: mod 0.48 Oligo-p: mod 0.45 |
| Minden 2014 | Registry based, prospective cohort, longitudinal study | 654 | Pain NRS vs PhGA | Longitudinal | Not reported | **Mixed** Subgroup / care-setting divergence | Physician–patient disease activity correlations were r = 0.56 in paediatric care and r = 0.51 in adult care. In adult care, patients reported higher pain (mean 2.8; 78% with pain) while clinicians rated disease activity lower (mean 1.9) at last follow-up. |
| Oen 2009 | Prospective cohort  Registry based (ReACCh-Out inception cohort) | 356 | Pain VAS vs PhGA | Concurrent | Spearman correlation | **Discordant** | Pain VAS vs PhGA: 0.28 Pain correlates more strongly with patient-reported outcomes (e.g., JAQQ, C-HAQ) than with physician-assessed or objective clinical measures. |
| Oen 2018 | Prospective cohort  Registry-based (ReACCh-Out) | 1249 | Pain VAS (prev week) vs PhGA (Time to reach best possible scores) | Longitudinal | Kaplan–Meier curves (time to best score) | **Mixed** Time/outcome divergence | Improvement in pain and HRQoL lagged 1–4 years behind improvement in disease activity, suggesting broader multidimensional influences on perceived well-being. |
| Palmisani 2006 | Cross-sectional | 223 | Parent pain VAS vs PhGA | Concurrent | Spearman correlation | **Concordant** | Parent pain vs PhGA: Moderate correlation across all cohorts: Early (0.42), Advanced (0.68), Longstanding (0.47) |
| Randall 2024 | Cross-sectional observational study (CARRA registry) | 355 | Pain Interference (PROMIS PI) vs PhGA | Concurrent | Spearman correlation; ANOVA; linear regression | **Discordant** | In the bivariate analysis:  Higher PI in children with active disease (PhGA >1), however, in multivariable analyses, the magnitude of these relationships was small, and most clinical/demographic variables, including disease activity and treatments, lost statistical significance. |
| Rashid 2018 | Prospective inception cohort (CAPS) with pain trajectories | 851 | Pain VAS trajectories vs PhGA at presentation and over time | BL AJC; pain followed for 3 years | Multinomial logistic regression; Kruskal–Wallis tests | **Mixed**  Trajectory divergence | Low pain group had low PhGA, but PhGA (and AJC) did not predict consistently high pain trajectories. Persistently high pain likely reflects non-inflammatory mechanisms, altered pain processing, age at onset, disease duration, and psychosocial factors rather than disease activity alone. |
| Ravelli 1997 | Cross-sectional | 55 | Parent pain (prev week) vs PhGA | Concurrent | Pearson correlation | **Discordant** | Parent pain VAS was not strongly correlated with articular variables or lab markers Parent pain vs PhGA r = 0.35 Colinearity is common within categories of disease activity measures (e.g., subjective, functional, articular, laboratory), but uncommon among variables from different categories |
| Ross 1989 | Cross-sectional Validation | 101 | Pain VAS vs: Joint inflammation (physician)  Pain VAS vs: PhGA  Pain VAS vs morning stiffness (parent/child reported)  Pain VAS vs composite of the above | Concurrent | ANOVA  Spearman correlation  Multiple regression | **Concordant** | Reported pain was a valid indicator of clinical state, especially in polyarticular JRA. Contrary to prior assumptions, younger children with pauciarticular disease reported higher pain than older children, and age was not a significant predictor. |
| Rypdal 2024 | Prospective longitudinal cohort study; 18-year follow-up of the Nordic JIA cohort | 427 | At 18 year follow up: Pain VAS vs PhGA | Concurrent | Pearson correlation | **Concordant** | At 18 year follow up, pain VAS was moderately correlated with PhGA (r = 0.61) |
| Schanberg 1997 | Cross-sectional | 56 | Oucher vs PhGA  Pain VAS vs PhGA  Pain location (body map) vs PhGA | Concurrent | Spearman correlation  Hierarchical regression (controlling for age, disease activity, disease duration) | **Concordant** | Current disease activity explained a significant proportion of variance in pain thermometer ratings (28%), Oucher scale ratings (33%), and number of pain locations (13%)  Higher levels of disease activity were related to higher pain ratings and a greater number of reported pain locations. |
| Schanberg 2003 | Longitudinal | 41 | Pain VAS vs PhGA Percentage of pain days vs PhGA | PhGA at BL; pain measured daily over 2 months | Pearson correlations Independent samples t-tests General linear models Multilevel mixed-effects models | **Concordant** | Higher PhGA was associated with greater pain, including higher daily pain (r = 0.39), a higher proportion of pain days in active disease (85% vs 58%), and higher average pain intensity on pain days compared with minimal disease activity. |
| Sgarlat 2011 | Cross-sectional secondary analysis of registry data (CARRAnet, 2010-2011) | 2618 | Pain NRS vs PhGA | Concurrent | Pearson correlation | **Discordant** | Pain NRS showed a modest association with PhGA (c = 0.43), which was substantially weaker than the association with patient/parent global assessment (c = 0.75), indicating limited concordance between pain and physician-rated disease activity. |
| Shoop-Worrall 2021 | Multicentre prospective longitudinal study with joint trajectories | 1184 | Pain VAS vs PhGA trajectory profiles | BL and annual data over 3 years | Latent profile analysis; multivariate group-based trajectory modelling | **Mixed** Trajectory divergence | 78% showed parallel change in pain and PhGA; 22% (Low-Persistent and High-Persistent) had persistent high pain despite low or improving PhGA. Discordant groups may have non-inflammatory or extra-articular pain and greater social disadvantage; anti-rheumatic therapy alone may not address symptoms. |
| Spekking 2023 | RCT with longitudinal follow-up over 24 months Multicentre, single-blind, three-arm RCT comparing different treat-to-target strategies | 92 | Pain VAS (prev week) at FU vs Baseline PhGA | PhGA at BL; Pain at FU | Linear mixed models (random intercept & slope) Multivariable model (prediction) Univariable analysis Multiple imputation | **Discordant** | Lower baseline PhGA was counterintuitively associated with higher follow-up pain (β = –0.31), indicating that some children continue to experience pain despite low or inactive disease activity, highlighting the importance of addressing patient-reported outcomes in addition to inflammatory disease control. |
| Thompson 1987 | Validation | 23 | Pain VAS vs PhGA (disease activity index) | Concurrent | Pearson correlation  Welch’s V statistic | **Concordant** | More intense present pain ratings were associated with more active disease |
| Trachtman 2021 | Secondary analysis of RCT or cohort study Nested cohort study within a clinical trial | 120 | Pain VAS (prev week) vs PhGA | Longitudinal | Spearman correlations Multivariable logistic regression Area under the curve (AUC) | **Mixed** Time / outcome divergence | Discordant prior to flare, with higher pain despite minimal PhGA; concordant at flare onset. Pain increased before flare while PhGA remained near zero (mean PhGA 0.0 vs pain 0.4), whereas at flare pain and PhGA were correlated (r = 0.45). Predictive value for flare was weak for both measures (AUC 0.51–0.58) |
| Tucker 2009 | Prospective longitudinal inception cohort study Part of the ReACCh-Out study across 16 Canadian pediatric rheumatology centres | 321 | Pain VAS vs PhGA | Concurrent (baseline and 6 months) | Spearman correlations  Regression analysis | **Concordant** | Physician and patient/parent global assessments were strongly associated with pain Objective measures of disease activity were the strongest predictors of pain, while demographic and socioeconomic variables did not influence pain in this cohort |
| Tupper 2013 | Secondary analysis of prospective observational studies. Longitudinal study (data collected 3 times per day for 7 days) | 85 | Pain variability vs PGADS | PGADS at BL clinic exam; pain diaries 3×/day for 7 days FU | Generalized estimating equations (GEE) Logistic regression Linear regression | **Concordant** | Higher disease severity (PGADS) predicted greater pain variability (β = 0.022), with a 25% increase in odds per 10-unit increase, highlighting pain variability as an important consideration in both research design and clinical education. |
| van Dijkhuizen 2023 | Validation | 6060 JIA 8643 parents | Pain VAS vs PhGA | Concurrent | Spearman correlations (validity) Intraclass correlation coefficient (ICC) (reliability) Bland–Altman approach (reliability) | **Concordant** | Parent-reported Pain VAS vs PhGA 0.57  Patient-reported Pain VAS vs PhGA 0.56 |
| Vandvik 1990 | Cross-sectional | 57 | Present pain VAS vs PhGA Worst pain VAS vs PhGA | Concurrent | Spearman correlation | **Discordant** | No significant association was found between physician-rated disease severity and either present pain (r = 0.06) or worst pain (r = −0.06), suggesting discordance that may reflect differing physician and patient perspectives on disease severity. |
| Varni 1987 | Validation | 25 | Child, Parent Pain VAS (present pain) vs PhGA | Concurrent | Correlations  Paired t-tests | **Concordant** | As physician-rated disease activity increased, there was a corresponding increase in rated present pain intensity by the child, parent, and physician |
| Verstappen 2015 | Secondary analysis of RCT/cohort (baseline cross-sectional) | 923 | Pain VAS vs PhGA across socioeconomic groups | Concurrent | Descriptive statistics; regression models for discordance vs SES | **Mixed** Subgroup divergence (lower SES) | Low-SES children reported higher pain despite similar PhGA/AJC and other disease activity scores. Clinical scores did not fully capture symptom burden in low-SES children; unmeasured comorbidities or social stressors may contribute. |
| Weiss 2012 | Retrospective cross-sectional analysis of 2,571 children with JIA from the CARRA Registry | 2571 | Pain intensity vs PhGA | Concurrent | Multivariable linear regression (pain & function) Ordinal logistic regression (health status) Correlations (PGA & patient-reported outcomes) | **Discordant**  Pain > Disease activity | Pain intensity was weakly associated with PhGA (r = 0.43), with most children reporting pain despite low disease activity, indicating that clinical measures alone do not fully capture children’s pain experience. |

Concordance categories in this table are assigned at the marker level. Individual studies may therefore appear as concordant for a given marker despite being classified as mixed at the study level when multiple markers were considered.

*AJC = active joint count ANOVA = analysis of variance AUC = area under the curve BL = baseline CAPS = Childhood Arthritis Prospective Study CARRA = Childhood Arthritis and Rheumatology Research Alliance CARRAnet = Childhood Arthritis and Rheumatology Research Alliance network ERA = enthesitis-related arthritis FU = follow-up HRQoL = health-related quality of life ICC = intraclass correlation coefficient JAQQ = Juvenile Arthritis Quality of Life Questionnaire JIA = juvenile idiopathic arthritis JRA = juvenile rheumatoid arthritis NRS = numeric rating scale PGADS = physician global assessment of disease severity PhGA = physician global assessment PI = pain interference PROMIS = Patient-Reported Outcomes Measurement Information System RCT = randomized controlled trial ReACCh-Out = Research in Arthritis in Canadian Children Emphasizing Outcomes RF = rheumatoid factor SES = socioeconomic status VAS = visual analogue scale*

**Table S5.** Active joint count (AJC)

| First author/Year | Design | Sample | Specific comparison | Time-point | Analysis | Relationship | Summary of author findings |
| --- | --- | --- | --- | --- | --- | --- | --- |
| Alongi 2022 | Cross-sectional pooled registry data (incl. EPOCA) | 9966 | Parent pain VAS vs inactive joints (AJC = 0) | Concurrent | Multivariable logistic regression; dominance analysis | **Discordant** Pain > Disease activity | Pain VAS > 0 was the strongest driver of non-zero PhGA during periods when AJC = 0. |
| Bandeira 2006 | Cross-sectional analysis of two JIA cohorts | 121 | Parent pain VAS vs AJC (simple and weighted) | Concurrent | Spearman correlation | **Mixed** Disease-duration subgroup divergence | Correlation weak in Cohort 1 (DD ≈ 3.6 yrs) but moderate in Cohort 2 (DD ≈ 7.7 yrs); weighted counts aligned better with pain. |
| Chaplin 2018 | Cross-sectional | 85 | Pain intensity (APPT) vs AJC | Concurrent | Spearman correlation; hierarchical multiple linear regression | **Discordant** | AJC only modestly associated with pain (rs≈0.27) and effect largely mediated by illness perceptions. Illness beliefs (identity and consequences) accounted for most of the AJC–pain association; clinical activity alone did not explain pain. |
| Coulton 1987 | Cross-sectional validation study | 77 | Pain dimension (AIMS) vs AJC; pain vs active/inactive JRA classification | Concurrent | Correlation coefficients; t-tests; discriminant analysis | **Concordant** | Higher AJC associated with higher pain in active disease (r≈0.35); pain discriminated active vs inactive groups. |
| de Vos 2023 | Retrospective clinical data | 778 | Pain VAS vs AJC (AJC > 0 vs AJC = 0) | Rheumatologist assessment within 0–14 days of JAMAR (near-concurrent) | χ² tests; t-tests; Kruskal–Wallis tests | **Concordant** | Higher pain in children with AJC > 0 |
| Guzman 2017 | Prospective multicentre inception cohort (ReACCh-Out) | 609 | Pain VAS (prev week) vs AJC in Severe Controlled vs Severe Persisting subgroups | Concurrent and longitudinal | Spearman correlation (post-hoc) | **Mixed** Subgroup divergence | Stronger concordance in Severe Controlled (r≈0.46); weaker in Severe Persisting (r≈0.30) |
| Hogewe 1995 | Cross-sectional | 57 | Pain VAS (prev week) vs AJC | Concurrent | Pearson correlations | **Concordant** | Moderate positive correlation (r≈0.43) |
| Kovalchuk 2018 | Cross-sectional (reactive arthritis vs controls) | 30 | Child and parent pain VAS vs AJC | Concurrent | Spearman correlation | **Discordant** | Low correlations (child r≈0.36; parent r≈0.31) |
| Learoyd 2019 | Retrospective longitudinal observational cohort | 97 | Present pain VAS vs AJC at visits; pain trajectory vs baseline and average AJC | Three clinic visits over 50 weeks | Linear regression; ANOVA; multinomial logistic regression; χ²/Fisher tests | **Mixed** Trajectory divergence | Baseline AJC associated with high pain trajectories, but pain at individual visits not linked to concurrent AJC; some high-pain trajectories had fewer active joints. Findings support a role for prior inflammation and individual pain sensitivity (priming/central sensitisation) rather than current AJC alone in explaining persistent high pain. |
| Malleson 2004 | Secondary analysis of cohort/RCT | 388 | Pain VAS (worst prev week) vs AJC | Concurrent | Spearman correlation; stepwise linear regression | **Discordant** | AJC correlated with pain (r≈0.42) but explained only a small proportion of variance; not an independent predictor in older adolescents. Pain is multifaceted and cannot be explained solely by disease factors; coping, mood, and family context are likely important. |
| McGrath 2023 | Case–control nested in ReACCh-Out | 149 | Pain VAS trajectory vs baseline active joint and enthesitis counts | BL AJC vs longitudinal pain | Logistic regression; linear mixed models | **Concordant** | Higher baseline AJC and enthesitis in the persisting pain group; enthesitis remained strongest independent predictor. Female sex, widespread enthesitis, and lower quality of life identified children at risk of persisting pain; authors call for early pain management in addition to inflammation control. |
| Miller 2011 | Cross-sectional, retrospective | 160 | Pain NRS (prev week, average) vs AJC | Concurrent | Spearman correlation | **Discordant** | Correlations between pain scores and joint counts in all JIA subtypes tended to be lower than other correlations |
| Oen 2009 | Prospective cohort (ReACCh-Out) | 356 | Pain VAS vs AJC | Concurrent | Spearman correlation; multivariable models | **Discordant** | AJC had the strongest correlation with pain among objective markers (r≈0.38) but still only weak. Pain correlated more strongly with patient-reported outcomes than with physician or laboratory measures. |
| Oen 2018 | Prospective registry-based cohort (ReACCh-Out) | 1249 | Time to best pain VAS vs time to best AJC | Longitudinal | Kaplan–Meier curves | **Mixed** Time/outcome divergence | Improvement in pain and HRQoL lagged 1–4 years behind improvement in disease activity, suggesting broader multidimensional influences on perceived well-being. |
| Palmisani 2006 | Cross-sectional across disease-duration strata | 223 | Parent pain score vs number of active joints (AJC) | Concurrent | Spearman correlation | **Mixed**  Subgroup divergence | Low correlation in early disease (r=0.35); moderate in advanced (0.54) and longstanding (0.41) |
| Randell 2024 | Cross-sectional observational study (CARRA registry) | 355 | Pain Interference (PROMIS PI) vs AJC | Concurrent | Spearman correlation; ANOVA; linear regression | **Discordant** | PI increased with number of active joints (R²≈0.09) but disease activity explained little variance in multivariable models. Functional and psychosocial factors were more strongly related to pain interference than clinical measures such as AJC. |
| Rapoff 1991 | Cross-sectional validation study | 31 | Parent-reported pain complaints vs AJC | Concurrent | Pearson correlation; multiple regression | **Concordant** | Parent pain complaints correlated with AJC r≈0.46. |
| Rashid 2018 | Prospective inception cohort (CAPS) with pain trajectories | 851 | Pain VAS trajectories vs AJC at presentation and over time | BL AJC; pain followed for 3 years | Multinomial logistic regression; Kruskal–Wallis tests | **Mixed**  Trajectory divergence | Low pain group had low AJC, but AJC (and PhGA) did not predict consistently high pain trajectories. Persistently high pain likely reflects non-inflammatory mechanisms, altered pain processing, age at onset, disease duration, and psychosocial factors rather than AJC alone. |
| Ravelli 1997 | Cross-sectional | 55 | Parent pain (prev week) vs AJC | Concurrent | Pearson correlation | **Discordant** | Parent pain VAS was not strongly correlated with articular variables or lab markers. Parent pain vs AJC r = 0.26 Colinearity is common within categories of disease activity measures (e.g., subjective, functional, articular, laboratory), but uncommon among variables from different categories |
| Rypdal 2024 | Prospective longitudinal Nordic cohort (18-year follow-up) | 427 | Pain VAS vs AJC at 18-year follow-up | Concurrent | Pearson correlation | **Mixed** Subgroup divergence | Moderate correlation (r≈0.56), though some groups had low AJC with higher perceived disease burden. High PaGA despite low AJC suggests contributions from damage, disability, non-inflammatory pain, fatigue, and psychosocial factors; multidisciplinary care is recommended. |
| Schanberg 2003 | Longitudinal daily diary study | 41 | Baseline AJC vs daily pain VAS and % pain days | BL AJC; pain over 2 months | Pearson correlation; GLM; mixed-effects models | **Concordant** | Higher baseline AJC associated with more daily pain (r≈0.36) and higher proportion of pain days. |
| Shiff 2018 | Prospective longitudinal cohort with latent pain trajectories | 1062 | Pain VAS trajectories vs baseline AJC | BL AJC; pain over 5 years | Latent trajectory analysis; t-tests; χ² tests; logistic regression | **Mixed** Trajectory divergence | Higher baseline AJC predicted several higher-pain trajectories, but disease activity explained only a small proportion of pain variance and some trajectories showed paradoxical AJC–pain patterns. Chronic moderate and mild-increasing pain trajectories may reflect psychosocial comorbidities or poorer coping rather than AJC alone. |
| Shoop-Worrall 2021 | Multicentre prospective longitudinal study with joint trajectories | 1184 | Pain VAS vs AJC trajectory profiles | BL and annual data over 3 years | Latent profile analysis; multivariate group-based trajectory modelling | **Mixed** Trajectory divergence | 78% showed parallel change in pain and AJC; 22% (Low-Persistent and High-Persistent) had persistent high pain despite low or improving AJC. Discordant groups may have non-inflammatory or extra-articular pain and greater social disadvantage; anti-rheumatic therapy alone may not address symptoms. |
| Spekking 2023 | Multicentre RCT with 24-month treat-to-target follow-up | 92 | Baseline AJC vs Pain VAS over follow-up; Pain VAS vs inactive disease (Wallace) | BL AJC; longitudinal pain over 24 months | Linear mixed models; multivariable prediction models | **Mixed** Trajectory divergence | Higher baseline AJC predicted higher pain, but 11% had moderate/severe pain despite achieving inactive disease. Some children experience pain despite inactive disease, possibly due to central sensitisation; authors advocate a dual target (inflammation + symptom control). |
| Tarakci 2011 | Cross-sectional observational study | 52 | Pain VAS vs number of active joints (AJC) | Concurrent | Pearson correlation | **Discordant** | Significant but weak correlation r≈0.30 |
| Tucker 2009 | Prospective longitudinal inception cohort (ReACCh-Out) | 321 | Pain VAS vs AJC; change in pain vs change in AJC at 6 months | Baseline and 6-month follow-up | Spearman correlation; regression analyses | **Concordant** | AJC independently predicted pain at baseline and 6 months; decreases in AJC associated with decreases in pain. Objective measures (especially AJC and ESR) were the strongest predictors of pain; demographic and socioeconomic variables were not influential. |
| van Dijkhuizen 2023 | Validation study of remote monitoring PROs | 6060 | Child and parent pain VAS (prev week) vs AJC | Concurrent | Spearman correlation; ICC for reliability | **Concordant** | Moderate correlations between pain and AJC (ρ≈0.43–0.45 for child and parent reports). |
| Verstappen 2015 | Secondary analysis of RCT/cohort (baseline cross-sectional) | 923 | Pain VAS vs AJC across socioeconomic groups | Concurrent | Descriptive statistics; regression models for discordance vs SES | **Mixed** Subgroup divergence (lower SES) | Low-SES children reported higher pain despite similar AJC and other disease activity scores. Clinical scores (including AJC) did not fully capture symptom burden in low-SES children; unmeasured comorbidities or social stressors may contribute. |
| Vuorimaa 2008 | Cross-sectional cluster analysis | 145 | Pain frequency vs AJC across psychological clusters | Concurrent | Cluster analysis; between-cluster comparisons | **Mixed** Subgroup / phenotype divergence | Some clusters showed high pain with high AJC, others high pain with low AJC or low pain with fairly high AJC. Relationship between disease parameters and pain is “not straightforward”; self-efficacy and emotional factors (anxiety, depression) mediate how AJC relates to pain. |
| Weiss 2012 | Retrospective cross-sectional registry analysis (CARRA) | 2571 | Pain intensity vs AJC (whole cohort and ERA subgroup) | Concurrent | Multivariable linear regression; correlation | **Mixed** Subgroup / phenotype divergence (not in ERA) | AJC was independently associated with pain in the full cohort but not in ERA once enthesitis and SI tenderness were included; ERA-specific features and psychosocial factors likely contribute to pain beyond AJC |

Concordance categories in this table are assigned at the marker level. Individual studies may therefore appear as concordant for a given marker despite being classified as mixed at the study level when multiple markers were considered.

*AJC = active joint count AIMS = Arthritis Impact Measurement Scales ANOVA = analysis of variance APPT = Adolescent Pediatric Pain Tool BL = baseline CAPS = Childhood Arthritis Prospective Study CARRA = Childhood Arthritis and Rheumatology Research Alliance CHAQ = Childhood Health Assessment Questionnaire DD = disease duration ERA = enthesitis-related arthritis ESR = erythrocyte sedimentation rate FU = follow-up GLM = general linear model HRQoL = health-related quality of life ICC = intraclass correlation coefficient ID = inactive disease JAMAR = Juvenile Arthritis Multidimensional Assessment Report JIA = juvenile idiopathic arthritis JRA = juvenile rheumatoid arthritis NRS = numeric rating scale PaGA = patient/parent global assessment PhGA = physician global assessment PI = pain interference PRO = patient-reported outcome PROMIS = Patient-Reported Outcomes Measurement Information System RCT = randomised controlled trial SES = socioeconomic status SI = sacroiliac VAS = visual analogue scale*

**Table S6.** Palpation tenderness

| First author/Year | Design | Sample | Specific comparison | Time-point | Analysis | Relationship | Summary of author findings |  |
| --- | --- | --- | --- | --- | --- | --- | --- | --- |
| Bandeira 2006 | Cross-sectional analysis of two cohorts from the Italian national database (cohort 1= consecutive pts seen in clinic; cohort 2= disease duration >5 years) | 121 Cohort 1: 60 Cohort 2: 61 | Parent Pain VAS vs TEN/POMC (simple and weighted) | Concurrent | Spearman correlation | **Concordant** | Cohort 1 reported higher pain VAS despite similar median swollen, painful, and active joint counts across cohorts. Weighted joint counts correlated more strongly with parent-reported pain than unweighted counts, suggesting better alignment with functional impact, particularly for functionally important joints. |  |
| Elliott 1997 | Case report | 1 | Pain VAS vs TJC | Concurrent | Narrative | **Concordant** | Pain and TJC both unchanged (while other inflammatory markers improved) |  |
| Hogewe 1995 | Cross-sectional | 57 69 controls | Pain VAS vs Ritchie Index | Concurrent | Pearson correlation | **Concordant** | Pain VAS was significantly correlated with the Ritchie Index (r = 0.47, *P* < 0.01). |  |
| McGrath 2023 | Case-control study nested in a prospective cohort (ReACCh-Out) | 31 cases (persisting pain) 118 controls (decreasing pain) | Pain VAS over time vs Baseline enthesitis count | Time-lagged: Objective BL → Pain FU | Logistic regression (univariate & multivariable, stepwise); Linear mixed models | **Concordant** | Higher enthesitis and AJC were seen in the persistent pain group; enthesitis remained a strong independent predictor. |  |
| Palmisani 2006 | Cross-sectional | 223 | Parent Pain score vs TEN/POMC | Concurrent | Spearman correlation | **Concordant** | Moderate correlation across all cohorts: Early (0.49), Advanced (0.59), Longstanding (0.47). Notably, this was the sole articular variable correlated with parent pain in early disease. |  |
| Rumsey 2020 | Secondary analysis of prospective cohort data (ReACCh-Out), longitudinal | 1371 214 with enthesitis | Pain VAS (prev week, average) vs Entheseal tenderness  Pain VAS (prev week, average) vs Number of affected enthesis sites | Concurrent | Linear mixed models | **Concordant** | Children with entheseal tenderness had worse pain throughout the follow-up compared to those without enthesitis, after adjusting for JIA category and other covariates. |  |
| Weiss 2012 | Retrospective cross-sectional analysis of 2,571 children with JIA from the CARRA Registry | 2571 | Pain NRS (prev week) vs SIJ tenderness (specifically in ERA patients | Concurrent | Multivariable linear regression | **Concordant** | Active sacroiliac tenderness exhibited a strong, significant, and independent association with increased pain intensity in ERA patients, directly correlating this objective finding with the pain experience. |  |

Concordance categories in this table are assigned at the marker level. Individual studies may therefore appear as concordant for a given marker despite being classified as mixed at the study level when multiple markers were considered.

*AJC = active joint count BL = baseline CARRA = Childhood Arthritis and Rheumatology Research Alliance ERA = enthesitis-related arthritis FU = follow-up JIA = juvenile idiopathic arthritis NRS = numeric rating scale POMC = painful or limited joint count ReACCh-Out = Research in Arthritis in Canadian Children emphasizing Outcomes SIJ = sacroiliac joint TEN/POMC = tenderness/pain on movement count TJC = tender joint count VAS = visual analogue scale*

**Table S7.** Imaging

| First author/Year | Design | Sample | Specific comparison | Region | Time-point | Method | Relationship | Summary of author findings | |
| --- | --- | --- | --- | --- | --- | --- | --- | --- | --- |
| Collin 2024 | Prospective longitudinal cohort study | 54 | Pain vs CBCT | TMJ | Concurrent and history of pain vs CT at FU | Fisher's exact test  Binomial logistic regression  Multinomial logistic regression | **Mixed** Time / outcome divergence | Concordant association observed between concurrent TMJ pain and TMJ deformity, with higher deformity prevalence in children reporting TMJ pain; self-reported TMJ pain and dysfunction predicted baseline TMJ deformity. However, baseline TMJ pain or dysfunction did not predict longitudinal development of TMJ changes. Limited morphological change, sparse assessment time points, and high disease control with bDMARDs may have contributed to the lack of longitudinal associations. | |
| Algergawy 2011 | Cross-sectional | 20 | Pain VAS vs US (synovial thickness; effusion volume) | Knee | Concurrent | Statistical test (not specified) | **Mixed** Clinical marker divergence | Concordant: Pain VAS vs effusion volume (r = 0.41) No relationship: Pain VAS vs synovial thickness (r = 0.21). | |
| Alqanatish 2011 | Case series | 5 | Pain (narrative/clinical) vs MRI/US (infrapatellar bursal swelling) | Deep  infra-patellar bursa | Concurrent | Narrative | **Discordant** Pain < Disease activity | Bursal swelling often painless despite clear objective evidence of inflammation on examination, US, and MRI. | |
| Biancardi 2015 | Cross-sectional | 28 | Pain VAS (previous week, average) vs US (grey-scale and power Doppler) | UL and LL | Concurrent | Spearman correlation | **Mixed** Anatomical site | Parent pain VAS showed moderate correlations with US findings in UL (r = 0.34–0.56) but low correlations for LL (knees and ankles) (r = 0.09–0.22). | |
| Bloom 1995 | Case series (retrospective descriptive analysis) | 6 | Pain (narrative/clinical) vs US | Biceps | Concurrent | Narrative | **Discordant**  Pain < Imaging findings | Five of six cysts were painless despite visible US-confirmed synovial pathology. | |
| Eboli 2022 | Cross-sectional pilot study | 23 | Pain VAS (previous week, average) vs US | TMJ | Concurrent | Descriptive statistics | **Discordant** | No strong agreement between US and clinical data for detecting signs of TMJ involvement. Larger cohorts and MRI comparison are needed to clarify the role of US in TMJ assessment. | |
| Hara 2017 | Case series | 3 | Pain (narrative/clinical) vs PD US | Knee; shoulder; elbow; wrist | Concurrent | Narrative | **Mixed** Anatomical site/subgroup divergence | **Concordant**: wrist pain associated with moderate PD signal.  **Discordant – Pain <Imaging findings**: absent pain despite prominent PD signal in both shoulders in one case. US is sensitive for detecting arthritis findings that may be missed by examination, lab tests or XR. US findings guided DMARD change when pain was present but other markers were normal. | |
| Magni-Manzoni 2009 | Cross-sectional | 32 | Parent pain VAS vs US | 52 joints | Concurrent | Spearman correlation; kappa statistics | **Discordant** | Parent pain VAS vs US: correlations were poor (r from −0.07 to −0.18). US findings did not correlate with subjective parent ratings, which is not surprising because these assess constructs only partly related to joint inflammation. | |
| Melchiorre 2010 | Prospective cohort | 68 | Pain (narrative/clinical) vs joint effusion (JE) on US | TMJ | Concurrent | Narrative | **Discordant**  Pain < Imaging findings | Only 2/46 children (4.3%) with US evidence of JE were symptomatic; the remaining 44 (95.6%) did not report joint pain. TMJ involvement in oligoarticular JIA is often asymptomatic and easily overlooked; clinical assessment alone is insufficient. | |
| Miyamae 2019 | Case series report | 3 | Pain (narrative/clinical) vs US | Multiple | Concurrent | Narrative | **Discordant**  Pain < Imaging findings | No pain reported despite active disease on US. Neural mechanisms and developmental stage of pain perception/verbalisation in young children may explain lack of reported pain; absence of pain contributed to delayed diagnosis. | |
| Mosa 2021 | Case control | 1 | Pain (narrative/clinical) vs US | Biceps tendon | Concurrent | Narrative | **Discordant**  Pain < Disease activity | Bicipital synovial cyst presented as painless swelling despite high systemic inflammation and highlights “non-articular synovitis” as the sole musculoskeletal manifestation of sJIA at onset, with painless but active disease. | |
| Vega-Fernandez 2023 | Prospective cohort (CARRA) | 30 | Pain VAS vs US synovitis | 42-joints | Concurrent (baseline and 3-month follow-up) | Spearman correlation | **Discordant** | Pain VAS showed weak, non-significant correlations with MSUS synovitis at baseline (rs ≈ 0.27–0.28) and no correlation at follow-up (rs ≈ 0.02–0.11). Pain did not correlate with MSUS synovitis at either visit. Cognitive, emotional, and situational factors may exaggerate or minimise self-reported pain and well-being. | |
| Flatø 2002 | Longitudinal retrospective cohort | 314 | Inflammatory back pain vs radiographic sacroiliitis | SIJ | Longitudinal | Chi-square test; Fisher’s exact test | **Concordant** | Inflammatory back pain was significantly more frequent in those with radiographic sacroiliitis both at follow-up and early in disease**.** |  |
| Hara 2017 | Case series | 3 | Pain (narrative/ clinical) vs XR | Knee; shoulder; elbow; wrist | Concurrent | Narrative | **Discordant** | Pain despite normal XR (although findings were evident on US). |  |
| Leksell 2012 | Cross-sectional, case control | 41 | Severe symptoms vs XR; severe impact on daily life vs XR; facial pain > or ≤ 2 times/week vs XR; pain at chewing vs XR | TMJ; shoulder | Concurrent | Spearman correlation | **Discordant** | No statistically significant association between orofacial pain/symptoms and TMJ structural changes on X-ray. Disabling orofacial pain and dysfunction cannot be predicted based solely on objective TMJ findings. |  |
| Palmisani 2006 | Cross-sectional | 223 | Parent pain vs Poznanski score | Wrist | Concurrent | Spearman correlation | **Discordant** | Radiographic damage (Poznanski score) showed at most moderate/low negative correlations with parent-reported pain, varying by disease stage. Parent pain may be influenced by other disease factors (e.g. morning stiffness, coping, psychosocial impact) and by different recall periods for parent vs physician assessments. |  |
| Svensson 2000 | Cross-sectional | 105 | Pain at rest and on function vs XR | TMJ | Concurrent | Chi-square test; Holm’s test | **Discordant**  Pain < XR findings | Condylar lesions on X-ray were associated with stiffness at mouth opening but not with TMJ pain. Pain reporting is subjective and variable; children may report arthritis-related pain less consistently than adults. |  |
| Svensson 2001 | Clinical and histological study | 12 | TMJ pain self report + pain on palpation vs OPG | TMJ | Concurrent | Narrative only | **Discordant** Pain < XR findings | Extensive structural TMJ damage with minimal pain; pain was not a reliable indicator of TMJ destruction. TMJ destruction may be painless due to chronicity or NSAID/steroid use; lower TMJ compartments were consistently affected, and structural damage does not necessarily produce pain (similar to adult RA). |  |
| Weiss 2012 | Retrospective cross-sectional analysis (CARRA Registry) | 2571 | Pain intensity vs radiographic damage (XR) | SIJ | Concurrent | Multivariable linear regression; ordinal logistic regression; correlations | **Discordant** | Radiographic evidence of joint damage was not an independent predictor of pain intensity in the full JIA cohort or ERA subgroup. Clinical exam, PhGA, and structural damage are insufficient to explain pain; factors such as stress, mood, and emotion regulation may contribute. Issues in how PGA is assessed may also weaken associations with pain. |  |
| Yilmaz 2010 | Retrospective | 11 | Pain (narrative/ clinical) vs XR | Hip; SIJ; pelvis | Concurrent | Narrative | **Discordant**  Pain > XR findings | Patients had pain and MRI evidence of inflammation, while pelvic radiographs remained normal. Plain pelvic X-rays showed no pathology despite MRI-visible inflammatory changes; MRI is considered the modality of choice for sacroiliac/pelvic disease in juvenile-onset SpA. |  |
| Bou Antoun 2017 | Retrospective longitudinal cohort | 20 | Buttock pain and/or SI joint tenderness (narrative/clinical) vs MRI sacroiliitis; inflammatory back pain vs MRI | SIJ | Clinical and lab data within 3 months of MRI | Fisher exact test; sensitivity, specificity, PPV, NPV | **Mixed** Anatomical site | Concordant: buttock pain/SI tenderness vs MRI sacroiliitis;  No relationship: inflammatory back pain vs MRI. IBP is less common in children and sacroiliitis can be clinically silent; only a few MRI-positive patients reported IBP. |  |
| Brandon 2021 | Retrospective longitudinal cohort | 46 | Change in pain (VAS/NRS) vs change in SPARCC inflammation score | SIJ | Longitudinal | Spearman correlation | **Discordant** | Change in pain showed only weak correlation with change in MRI inflammation. History of inflammatory back pain and pain to palpation have low PPV in children, so objective measures such as SPARCC SIS are important. |  |
| Carlsson 2023 | Case control | 45 | Pain VAS vs MRI | TMJ | Pain at baseline; MRI median 68 days after clinical exam | Mann–Whitney U; Spearman correlation (with multiple-testing correction) | **Mixed** Objective-measure divergence | Pain was related to TMJ bone marrow oedema and erosion, but many MRI abnormalities occurred without clear symptoms or psychosocial differences. TMJ MRI findings overlapped between JIA and controls; minor changes may reflect normal variation, growth, trauma, or degeneration. Well-controlled JIA and modern treatment likely reduced overt TMJ symptoms. |  |
| Ferrazzo 2019 | Case report | 1 | Pain vs MRI and CT | TMJ | Not reported | Narrative | **Discordant**  Pain < MRI findings | No pain despite important morphological changes in the right mandibular condyle, fossa and articular eminence. |  |
| Hospach 2014 | Retrospective | 13 | Pain (narrative/clinical) vs MRI | Cervical spine | Longitudinal | Descriptive statistics; narrative | **Discordant**  Pain < MRI findings | Persistent MRI abnormalities and skeletal changes despite resolution of pain. Limited range of motion was universal at onset and aligned better with MRI change than pain; pain alone is not a sufficient criterion for cervical spine involvement. |  |
| Katsicas 2018 | Cross-sectional | 30 | Pain VAS (average previous week) vs MRI; sacroiliac pain vs MRI; lumbar pain vs MRI | SIJ | Concurrent | ICC for readers; Kruskal–Wallis ANOVA with post-hoc tests | **Discordant** | No significant association between any pain measures and MRI sacroiliitis. |  |
| Küseler 2005 | Prospective longitudinal cohort (2-year) | 15 | Pain (narrative/clinical) vs MRI | TMJ | Concurrent (same day) and longitudinal | Linear correlation (total clinical score vs MRI); descriptive/narrative for symptoms vs MRI | **Discordant** | Low symptom reporting but high rates of synovial enhancement. TMJ symptoms and signs are not characteristic of JIA; TMJ inflammation may stay subclinical and active for years without symptoms. |  |
| Mahmood 2014 | Retrospective | 43 | Pain (narrative/clinical) vs MRI | Cervical spine | Not reported/unclear | Narrative | **Mixed** Subgroup divergence | A subset of patients had no pain despite abnormal cervical MRI findings. Cervical spine involvement may be clinically silent until advanced; early MRI is important to guide timely intervention. |  |
| Malattia 2010 | Cross-sectional | 22 | Pain VAS (average previous week) vs MRI | Hip; wrist | Concurrent | Spearman correlation | **Mixed** Anatomical site | Concordant for wrist (dynamic MRI parameter REE correlated with pain); No relationship for hip (pain did not correlate with MRI parameters).Hip joints showed lower inflammatory activity on MRI; clinical examination may miss low-grade hip inflammation, risking structural damage despite apparent clinical control. |  |
| McLellan 2023 | Cross-sectional | 57 | Pain (narrative/clinical) vs MRI | TMJ | Concurrent | Narrative | **Mixed** Subgroup divergence | 78% of TMJ scans were abnormal with 53% indicating joint damage. 29% of patients did not have pain, but other symptoms (restricted movement, jaw swing). |  |
| Mohammed 2012 | Cross-sectional, case control | 40 | Pain VAS vs total MRI score; TMJ symptoms (yes/no) vs MRI abnormalities | TMJ | Concurrent | Pearson correlation; t-tests; chi-square; ANOVA | **Mixed** Objective-measure divergence | No relationship: presence/absence of TMJ symptoms vs MRI Pain < Disease activity: many asymptomatic joints Concordant: in those with pain, higher pain VAS correlated with worse MRI scores. TMJ involvement is often asymptomatic and difficult to examine; symptoms underestimate inflammation. Normal symptoms or exam do not exclude TMJ disease; clinical findings together with MRI give a better picture. |  |
| Nistala 2007 | Retrospective | 34 | Hip pain (history/clinical) vs MRI | Hip | Clinical assessment and MRI within 3 months | Pearson correlation; kappa; chi-square; multiple regression | **Discordant** | Hip pain did not significantly predict MRI activity scores in multivariable models. Inflammation without clinical signs reflects MRI’s high sensitivity; clinical assessment of active hip arthritis has a limited relationship with MRI, especially when damage is present |  |
| Pedersen 2008 | Prospective longitudinal cohort (2-year) | 15 | TMJ pain at rest/movement vs MRI/OPG | TMJ; cervical spine | Longitudinal | Narrative | **Discordant** Pain < MRI findings | Inflammatory TMJ changes on MRI and OPG often occurred without pain. Subjective symptoms underestimate inflammatory TMJ findings; inflammation may start early without symptoms and later lead to dysfunction as remodelling and resorption progress. |  |
| Pinto 2018 | Case report | 1 | Pain (narrative/clinical) vs MRI | TMJ | Longitudinal | Narrative | **Discordant**  Pain < MRI findings | Severe bilateral condylar destruction on imaging despite the absence of pain. Juvenile TMD symptoms can fluctuate and are hard to assess in young children; pain is often proxy-reported, and antirheumatic therapy may mask symptoms. |  |
| Portelli 2014 | Cross-sectional and interventional | 53 | Jaw pain (questionnaire/narrative) vs MRI | TMJ | Concurrent | Narrative | **Discordant**  Pain < MRI findings | Condylar and mandibular changes and effusion were more common in asymptomatic than symptomatic children. TMJ evaluation is challenging, especially in young children where pain is proxy-reported; antirheumatic treatments may also mask symptoms. |  |
| Stoll 2010 | Retrospective | 143 | Pain (narrative/clinical) vs Sacroiliitis on imaging (MRI/XR) | SIJ | Concurrent | Narrative | **Mixed** Objective-measure divergence | Limited sensitivity of physical examination manoeuvres and plain radiography for detecting early sacroiliitis. They emphasize that early diagnosis often requires advanced imaging, such as MRI, which can detect acute changes like bone marrow edema even before chronic changes or symptoms develop. The presence of "silent sacroiliitis" highlights that relying solely on pain or clinical signs can indeed miss the diagnosis of axial disease. |  |
| Stoustrup 2016 | Cross-sectional | 172 | Pain (TMJ specific questionnaire) vs MRI  Pain (TMJ specific questionnaire) vs CBCT | TMJ | Concurrent | Not reported | **Mixed** Subgroup divergence | 88% of patients with TMJ pain had evidence of TMJ involvement on imaging. Evidence of TMJ involvement on imaging was present in 79% of patients who reported no TMJ pain. |  |
| Sudhakar 2022 | Cross-sectional observational | 20 | Pain VAS vs MRI bone marrow oedema score | Shoulder | Concurrent | Spearman correlation | **Discordant** | Pain VAS did not correlate significantly with MRI scores; many joints without clinical arthritis showed early MRI changes. Clinical signs and symptoms underestimate inflammation in deep joints such as the shoulder. |  |
| Tripalo Batoš 2020 | Cross-sectional | 27 | Pain VAS vs MRI findings (cervical, thoracic, lumbar spine, SIJ) | Hip; spine; SIJ | Concurrent | Spearman correlation; narrative | **Discordant** | Statistical analysis: no relationship; Narrative: Pain < Disease activity, since pain was not the leading sign even with positive cervical MRI findings. Pain may not be the leading clinical sign in juvenile SpA with axial involvement, especially when cervical MRI already shows pathology; small sample and early disease stage noted. |  |
| von Schuckmann 2020 | Retrospective | 2413 | Pain at rest vs MRI; pain while chewing vs MRI | TMJ | Concurrent | Logistic regression | **Mixed** Pain-measure / context | Concordant: pain while chewing associated with MRI-confirmed TMJ involvement; No relationship: pain at rest not significantly associated with MRI changes. Pain, particularly with chewing, has high PPV for TMJ involvement, but absence of pain has low NPV because many TMJ cases are asymptomatic. |  |
| Weiss 2016 | Prospective Cross-sectional | 40 | Back pain (current/history) vs MRI sacroiliitis; inflammatory back pain vs MRI sacroiliitis | SIJ | Concurrent | Fisher’s exact; Wilcoxon rank-sum; sensitivity/specificity; PPV/NPV; logistic regression | **Discordant** | Overall weak or no association between pain patterns and MRI findings. Most MRI-positive sacroiliitis cases were asymptomatic. Active sacroiliitis in children is often asymptomatic; adult axial SpA criteria relying on inflammatory back pain perform poorly in juveniles. Peripheral arthritis and enthesitis are more prominent early features. |  |
| Yamazaki 2022 | Retrospective | 7 | Inflammatory back pain and peripheral enthesitis vs MRI | SIJ | Longitudinal | Narrative | **Discordant** | Clinical severity of inflammatory back pain or enthesitis did not track with MRI findings. ASAS adult criteria may be less sensitive in children; early sacroiliac changes can be subtle and may not be detected on MRI. |  |
| Yilmaz 2010 | Retrospective | 11 | Pain (narrative/clinical) vs MRI | Hip/pelvis; SIJ | Concurrent | Narrative | **Concordant** | MRI consistently detected sacroiliitis and enthesitis in patients with clinical pain despite normal radiographs. Pelvic X-rays were normal in all patients; MRI is considered the imaging modality of choice in children and adolescents for sacroiliac and pelvic inflammatory or destructive changes. |  |
| Zwir 2012 | Longitudinal | 75 | Pain (narrative/clinical) vs MRI | TMJ | Concurrent | Narrative | **Discordant** Pain < MRI findings | Most children were asymptomatic despite abnormal TMJ MRI findings. TMJ should always be evaluated in JIA even without TMJ signs or symptoms; condylar damage can progress silently and synovial enhancement may be present even in clinical remission. |  |

Concordance categories in this table are assigned at the marker level. Individual studies may therefore appear as concordant for a given marker despite being classified as mixed at the study level when multiple markers were considered.

*ASAS = Assessment of SpondyloArthritis international Society bDMARD = biologic disease-modifying antirheumatic drug CBCT = cone-beam computed tomography CT = computed tomography ERA = enthesitis-related arthritis FU = follow-up IBP = inflammatory back pain ICC = intraclass correlation coefficient ID = inactive disease JE = joint effusion JIA = juvenile idiopathic arthritis LL = lower limb MRI = magnetic resonance imaging MSUS = musculoskeletal ultrasound NPV = negative predictive value OPG = orthopantomogram PD = power Doppler PhGA = physician global assessment PPV = positive predictive value REE = rate of early enhancement SIJ = sacroiliac joint SIS = sacroiliac inflammation score SPARCC = Spondyloarthritis Research Consortium of Canada sJIA = systemic juvenile idiopathic arthritis TMJ = temporomandibular joint UL = upper limb US = ultrasound VAS = visual analogue scale XR = X-ray*

**Table S8.** *Erythrocyte sedimentation rate (ESR) and C-reactive protein (CRP)*

| First author/Year | Design | Sample | Specific comparison | Timepoint | Analysis | Relationship | Summary of author findings |
| --- | --- | --- | --- | --- | --- | --- | --- |
| Elliott 1997 | Case report | 1 | Pain VAS (current/present) vs ESR and CRP | Repeated concurrent | Narrative | **Discordant** | Pain > Disease activity |
| Hogewe 1995 | Cross-sectional | 57 | Pain VAS prev week vs CRP | Concurrent | Pearson correlation | **Concordant** | Pain VAS prev week vs CRP: (r = 0.58) |
| Katsicas 2017 | Cross-sectional | 29 | Lumbar pain narrative vs ESR SIJ pain narrative vs ESR | Concurrent | Mann-Whitney U test, correlation tests | **Discordant** | No relationship/association |
| Kovalchuk 2018 | Cross-sectional | 60 | Child pain VAS present vs ESR and CRP Parent pain VAS present vs ESR and CRP | Concurrent | Spearman correlation | **Discordant** | No relationship/association |
| Miyamae 2019 | Case series | 40 | Pain narrative vs ESR and CRP | Concurrent | Narrative | **Discordant** Pain < Disease activity | No pain despite evidence of active disease |
| Oen 2009 | Longitudinal cohort | 356 | Pain VAS vs ESR and CRP | Concurrent | Spearman correlation | **Discordant** | ESR 0.17; CRP 0.14 ESR and CRP weakly associated with pain; not predictive in multivariable models. Pain correlates more strongly with PRO than with physician-assessed or objective clinical measures. |
| Palmisani 2006 | Cross-sectional | 223 | Parent pain vs ESR and CRP | Concurrent | Spearman correlation | **Mixed** Subgroup divergence (disease duration) | ESR: Early 0.46; Adv 0.38; Long 0.23  CRP: Early 0.35; Adv 0.26; Long 0.34  ESR moderate in early disease, weak later CRP weak in all cohorts |
| Rapoff 1991 | Validation | 31 | Parent pain frequency vs ESR | Concurrent | Multiple regression  Pearson correlation | **Discordant** | No relationship/association Lack of correlation between pain and ESR was expected, as ESR may not accurately reflect disease activity in JRA. ESR can be normal in severe disease or elevated in mild cases |
| Ravelli 1997 | Cross-sectional | 55 | Parent pain VAS prev week vs ESR and CRP | Concurrent | Pearson correlation | **Discordant** | ESR 0.31 CRP 0.17  Co-linearity is common within categories of disease activity measures (e.g., subjective, functional, articular, laboratory), but uncommon among variables from different categories. |
| Schanberg 2003 | Longitudinal | 41 | Pain VAS daily via diary vs Baseline ESR | ESR at BL; pain measured daily over 2 months | Pearson correlations | **Discordant** | No relationship/association Daily symptoms, including pain intensity, were unrelated to baseline ESR. Lack of relationship suggests that ESR might not capture daily fluctuations in symptoms as effectively as other measures. |
| Tucker 2009 | Prospective longitudinal inception cohort (ReACCh-Out) | 321 | Decrease in pain vs ESR at 6 months | Concurrent | Spearman correlation; regression analyses | **Concordant** | Objective measures (especially AJC and ESR) were the strongest predictors of pain; demographic and socioeconomic variables were not influential. |
| Upadhyay 2021 | Observational pilot | 16 | Pain NRS prev week vs ESR and CRP | Concurrent | Spearman correlation | **Discordant** | No relationship/association Pain in JIA can persist independently of inflammation. Important to understand and address JIA pain directly, rather than solely focusing on inflammatory markers. |
| van Dijkhuizen 2023 | Validation | 6060  (+8643 parents) | Parent pain VAS prev week vs ESR Patient pain VAS prev week vs ESR | Concurrent; VAS, 7–14 days apart) | Spearman correlation | **Discordant** | Weak/inconsistent  Parent-reported 0.22  Patient-reported 0.20  Low correlation with ESR implies that systemic inflammation, as indicated by ESR, is not a strong reflection of perceived pain intensity. |
| Zhang 2025 | Retrospective cohort | 165 | Pain VRS vs ESR and CRP | Concurrent | Kruskal–Wallis | **Discordant** | No relationship/association  Non-significant |

Concordance categories in this table are assigned at the marker level. Individual studies may therefore appear as concordant for a given marker despite being classified as mixed at the study level when multiple markers were considered.

*AJC = active joint count BL = baseline CRP = C-reactive protein ESR = erythrocyte sedimentation rate JIA = juvenile idiopathic arthritis NRS = numeric rating scale PRO = patient-reported outcome ReACCh-Out = Research in Arthritis in Canadian Children Emphasising Outcomes VAS = visual analogue scale VRS = verbal rating scale*

**Table S9.** Other blood biomarkers

| First author/Year | Biomarker | Design | Sample | Specific comparison | Timepoint | Analysis | Relationship | Summary of author findings |
| --- | --- | --- | --- | --- | --- | --- | --- | --- |
| Elfving 2023 | Proteomics panel | Clinical explorative; Longitudinal | 51 | Only significant correlations: Pain VAS vs IL-18R1, TGFβ, GDNF, FGF21, VEGFA, S100A12 | Concurrent | Spearman correlation; Mann-Whitney U; Wilcoxon signed-rank; T-tests | **Mixed** Clinical marker divergence | Concordant: Unadjusted: GDNF 0.33; TGFβ 0.34; IL-18R1 0.37 GDNF correlated with pain but not JADAS27 All Lost significance after correction No relationship: IL-6, S100A12, TNFα, and IL-1 |
| Elliott 1997 | Cytokines and related proteins: SAA, IL-6, sTNFR p55 and p75, IL-1rα, TNF-α | Case report | 1 | Pain VAS (current/present) vs SAA, IL-6, sTNFR p55 and p75, IL-1rα, TNF-α | Repeated concurrent | Narrative | **Discordant** | Pain > Disease marker activity: SAA, IL-6, sTNFR p55/75, IL-1ra Pain<Disease marker activity: TNF-α |
| Gorczyca 2017 | MRP8/14 | Cross-sectional | 44 | Pain VAS vs MRP8/14 | Concurrent | Not reported | **Concordant** | MRP8/14 positively correlated with pain VAS. |
| Hara 2017 | MMP-3 | Case series | 3 | Pain (narrative/clinical) vs MMP-3 | Concurrent | Narrative | **Discordant** Pain > Disease activity | Wrist pain but normal MMP-3. |
| Hu 2019 | Histone | Longitudinal, laboratory-based | 26 | Pain (narrative/clinical) vs Histone | Concurrent | Chi-square tests | **Discordant** | Histone levels significantly affected other clinical manifestations (fever, rash, hepatosplenomegaly, serositis) but not joint pain. |
| Katsicas 2017 | Th1/17 (T-helper immune pathway markers) | Cross-sectional | 29 | Lumbar pain narrative vs Th1/17 SIJ pain narrative vs Th1/17 | Concurrent | Mann-Whitney U | **Mixed** Clinical marker divergence | Concordant: Lumbar pain vs Th17 0.83  No relationship: Lumbar pain vs Th1; SIJ pain vs Th1/17 Lumbar Pain (LP): Th17 cells showed a strong correlation with lumbar pain despite not correlating with AJC. |
| Kovalchuk 2018 | Cytokines, immune markers, antibodies: CD3, CD4, CD8, CD16, CD22, IgA, IgM, IgG, IgE, TNF-α, IL-4, IL-8 | Cross-sectional | 60 | Pain VAS (current/present) Child pain VAS vs CD3/4+ Parent pain VAS vs CD3/CD4+  Only significant correlations reported (all other biomarkers not significant) | Concurrent | Spearman correlation | **Discordant** | Weak/Inconsistent: Child Pain VAS vs CD3+ -0.34; Child Pain VAS vs CD4+ -0.36 No Relationship/Association: Parent pain VAS vs CD3+ Parent pain VAS vs CD4+ CD8, CD16, CD22, IgA, IgM, IgG, IgE, TNF-α, IL-4, IL-8 |
| Miyamae 2019 | MMP-3 | Case series | 40 | Pain (narrative/clinical) vs MMP-3 | Concurrent | Narrative | **Discordant**  Pain < Disease activity | Neural mechanisms underlying pain in early childhood, particularly the developmental stage of pain perception and verbalization in younger children, may explain the lack of reported pain despite active disease. |
| Quilis 2024 | Cytokines and immune markers: IL-4, IL-6, IL-10, TGF-β, IFN-γ, Th1, Th2 | Cross-sectional with prospective follow up | 87 | Pain VAS prev week (JAMAR): Pain VAS vs Th1; Th2 Pain VAS vs IL-4, IL-6, IL-10, TGF-β, IFN- | Concurrent | Spearman correlation | **Discordant** | Weak/Inconsistent: TGF-β -0.327 borderline significant; IL-10- 0.220 No relationship/association: Treg, Th1, Th2, IFN-γ, IL-6 or IL-4 IL-10, and TGF-β may reflect an anti-inflammatory state and contribute to subjective well-being and reduced pain. |
| Radziszewska 2019 | Cytokines: IL-6, IL-12, IL-17, IL-23, IL-27, TNFα, IFN-γ | Cross-sectional | 14 | Pain VAS (night pain and past week) vs IL-6, IL-12, IL-17, IL-23, IL-27, TNF-α, IFN-γ | Concurrent | Not reported | **Discordant** | No correlation found between cytokine levels and pain scores (night or past week). |
| Spadaro 1996 | Cytokine and immune marker: IL-6, sIL-2R | Cross-sectional | 24 | Pain VAS vs IL-6, sIL-2R | Concurrent | Spearman correlation | **Mixed** Clinical marker divergence | Concordant: IL-6 .487  No relationship/association: sIL-2R 0.159 non-significant IL-6 is significantly correlated with both clinical disease activity and subjective pain severity (VAS), suggesting a role in the inflammatory process and the pain experience in JCA. sIL-2R, while elevated in some patients, did not show a significant correlation. |
| Van Der Heijden 2022 | Proteomics panel | Cross-sectional | 15 | Pain NRS prev week vs serum protein levels | Concurrent | Spearman correlation; Student t-test | **Mixed**  Clinical marker divergence | Proteomic analysis of 306 serum proteins identified 14 associated with pain severity, including PRG4, CST3, and VWF. Some proteins correlated with ESR while others did not, and several were associated with CNS structures, suggesting both inflammatory and non-inflammatory mechanisms of pain not captured by clinical disease activity measures. |
| Viswanath 2011 | MMP-3, TIMP-1 | Longitudinal cohort | 54 | Patient Pain VAS vs MMP-3 | Concurrent | Spearman correlation | **Discordant** | Pain VAS vs MMP-3 r = 0.28 |
| Xu 2016 | Cytokines: IL-17, IL-6, IL-21, IFN-γ, IL-4 | Cross-sectional | 60 | Pain VAS vs IL-17, IL-6, IL-21, IFN-γ, IL-4 | Concurrent | Spearman correlation | **Mixed** Clinical marker divergence | Pain VAS vs IL-17 (r = 0.524, p = 0.003)  IL-6 not significantly correlated with VAS |
| Zhang 2025 | Cytokines IL-6, IL-10, TNFα; Procalcitonin | Retrospective cohort | 165 | Pain VRS vs IL-10, IL-6, TNF-α, Procalcitonin (PCT) | Concurrent | Descriptive statistics; multivariate logistic regression; LTB & BCH methods; one-way ANOVA; Kruskal–Wallis; χ² tests with Bonferroni correction | **Mixed** Clinical marker divergence | Concordant for IL-10 only Higher IL-10 levels were associated with milder pain phenotypes, with increased odds of C2 (mild intermittent pain with extra-articular symptoms) versus C1 (moderate–severe inflammation-related pain). IL-10 may be a biomarker of pain modulation in sJIA, supporting early pain-focused evaluation. |

Concordance categories in this table are assigned at the marker level. Individual studies may therefore appear as concordant for a given marker despite being classified as mixed at the study level when multiple markers were considered.

*AJC = active joint count; BCH = Bolck–Croon–Hagenaars method; CNS = central nervous system; CRP = C-reactive protein; ESR = erythrocyte sedimentation rate; FGF21 = fibroblast growth factor-21; GDNF = glial cell line-derived neurotrophic factor; IFN-γ = interferon gamma; IL-1ra = interleukin-1 receptor antagonist; IL-2R = interleukin-2 receptor; IL-4 = interleukin-4; IL-6 = interleukin-6; IL-10 = interleukin-10; IL-12 = interleukin-12; IL-17 = interleukin-17; IL-18R1 = interleukin-18 receptor-1; IL-21 = interleukin-21; IL-23 = interleukin-23; IL-27 = interleukin-27; JAMAR = Juvenile Arthritis Multidimensional Assessment Report; JADAS = Juvenile Arthritis Disease Activity Score; MMP-3 = matrix metalloproteinase-3; MRP8/14 = myeloid-related protein-8/14; NRS/VAS/VRS = numeric/visual analogue/verbal rating scale; PCT = procalcitonin; PRG4 = proteoglycan-4; S100A12 = S100 calcium-binding protein A12; SAA = serum amyloid A; sIL-2R = soluble interleukin-2 receptor; sTNFR = soluble tumour necrosis factor receptor; Th = T-helper cell; TGF-β = transforming growth factor beta; TIMP-1 = tissue inhibitor of metalloproteinases-1; TNF-α = tumour necrosis factor alpha; VEGFA = vascular endothelial growth factor A; VWF = von Willebrand factor.*

**Table S10.** Disease activity markers used in studies reporting directional discordance between pain and disease activity in JIA

| Marker category | Low pain **Pain < disease activity marker**, n (%) | High pain **Pain > disease activity marker**, n (%) |
| --- | --- | --- |
| Imaging only | **16 (67)** | 0 (0) |
| Imaging + other markers | 4 (17) | 0 (0) |
| Any imaging included | **20 (83)** | 0 (0) |
| Physician-assessed clinical or composite measures | 2 (8) | **8 (67)** |
| Non-imaging multi-marker studies (clinical ± composite ± bloods) | 1 (4) | 3 (25) |
| Blood biomarkers only | 1 (4) | 1 (8) |
| Total studies | 24 (100) | 12 (100) |

*Directional discordance was defined as low or absent pain relative to disease activity markers (Pain < disease activity marker) or high or persistent pain relative to low disease activity markers (Pain > disease activity marker). Imaging-based studies most commonly examined joints that are frequently clinically silent, such as the temporomandibular and sacroiliac joints.*

References

1.	Abu-Saad HH and Uiterwijk M. Pain in children with juvenile rheumatoid arthritis: A descriptive study. *Pediatr Res* 1995; 38: 194-197. DOI: 10.1203/00006450-199508000-00010.

2.	Algergawy S, Haliem T and Al-Shaer O. Clinical, laboratory, and ultrasound assessment of the knee in juvenile rheumatoid arthritis. *Clin Med Insights Arthritis Musculoskelet Disord* 2011; 4: 21-27. DOI: 10.4137/CMAMD.S4371.

3.	Alongi A, Giancane G, Naddei R, et al. Drivers of non-zero physician global scores during periods of inactive disease in juvenile idiopathic arthritis. *RMD Open* 2022; 8: e002042. DOI: 10.1136/rmdopen-2021-002042.

4.	Alqanatish JT, Petty RE, Houghton KM, et al. Infrapatellar bursitis in children with juvenile idiopathic arthritis: A case series. *Clin Rheumatol* 2011; 30: 263-267. DOI: 10.1007/s10067-010-1620-2.

5.	Armbrust W, Kaak JG, Bouma J, et al. Assessment of disease activity by patients with juvenile idiopathic arthritis and the parents compared to the assessment by pediatric rheumatologists. *Pediatr Rheumatol* 2013; 11: 48. DOI: 10.1186/1546-0096-11-48.

6.	Arnstad ED, Rypdal V, Peltoniemi S, et al. Early Self-Reported Pain in Juvenile Idiopathic Arthritis as Related to Long-Term Outcomes: Results From the Nordic Juvenile Idiopathic Arthritis Cohort Study. *Arthritis Care Res* 2019; 71: 961-969. DOI: 10.1002/acr.23715.

7.	Arnstad ED, Iversen JM, Uglem M, et al. Pain sensitivity in young adults with juvenile idiopathic arthritis: a quantitative sensory testing study. *Arthritis Res Ther* 2020; 22: 262. DOI: 10.1186/s13075-020-02345-2.

8.	Bandeira M, Falcone A, Pistorio A, et al. Weighting improves the information provided by joint counts on the severity of arthritis and its impact on patients' well-being in juvenile idiopathic arthritis. *Rheumatology (Oxford)* 2006; 45: 343-347. DOI: 10.1093/rheumatology/kei161.

9.	Biancardi C, Borzani I, Ravelli A, et al. Which is the clinical relevance of ultrasound examination in the assessment of established juvenile idiopathic arthritis? *Ann Rheum Dis* 2015; 74: 381-382. DOI: 10.1136/annrheumdis-2015-eular.3825.

10.	Bloom BJ, Tucker LB, Miller LC, et al. Bicipital synovial cysts in juvenile rheumatoid arthritis: Clinical description and sonographic correlation. *J Rheumatol* 1995; 22: 1953-1955.

11.	Bou Antoun M, Adamsbaum C, Semerano L, et al. Clinical predictors of magnetic resonance imaging-detected sacroiliitis in children with enthesitis related arthritis. *Joint Bone Spine* 2017; 84: 699-702. DOI: 10.1016/j.jbspin.2017.07.003.

12.	Brandon TG, Becker BD, Bevans KB, et al. Patient‐Reported Outcomes Measurement Information System Tools for Collecting Patient‐Reported Outcomes in Children With Juvenile Arthritis. *Arthritis Care Res* 2017; 69: 393-402. DOI: 10.1002/acr.22937.

13.	Brandon TG, Xiao R, Peterson RG, et al. Changes over time in inflammatory and structural lesions at the sacroiliac joint in children with spondyloarthritis exposed and unexposed to tumor necrosis factor inhibitor. *Pediatr Rheumatol* 2021; 19: 167. DOI: 10.1186/s12969-021-00647-6.

14.	Carlsson AD, Wahlund K, Klintstrom E, et al. Juvenile idiopathic arthritis and the temporomandibular joint: a case-control study of magnetic resonance imaging findings in relation to clinical and psychosocial factors. *Eur J Paediatr Dent* 2023; 24: 69-76. DOI: 10.23804/ejpd.2023.24.01.12.

15.	Chaplin H, Ioannou Y, Sen D, et al. Exploring a biopsychosocial approach to pain in adolescents and young adults with juvenile idiopathic arthritis (young investigator award winner 2018). *Rheumatology (Oxford)* 2018; 57: iii34-iii35. DOI: 10.1093/rheumatology/key075.207.

16.	Collin M, Christidis N, Hagelberg S, et al. Temporomandibular involvement in children and adolescents with juvenile idiopathic arthritis: a 2-year prospective cohort study. *Sci Rep* 2024; 14: 5512. DOI: 10.1038/s41598-024-56174-3.

17.	Coulton CJ, Zborowsky E, Lipton J, et al. Assessment of the reliability and validity of the arthritis impact measurement scales for children with juvenile arthritis. *Arthritis Rheum* 1987; 30: 819-824. DOI: 10.1002/art.1780300713.

18.	De Oliveira Sato J, Corrente JE and Saad-Magalhães C. Progression of articular and extraarticular damage in oligoarticular juvenile idiopathic arthritis. *Clin Exp Rheumatol* 2011; 29: 871-877.

19.	De Vos ADW, Vastert B, Van Nieuwenhove E, et al. Does patient reported absence of swollen and painful joints really mean that the active joint count is zero? *Pediatr Rheumatol* 2023; 21. DOI: 10.1186/s12969-023-00879-8.

20.	Dekker M, Hoeksma AF, Dekker JHM, et al. Strong relationships between disease activity, foot-related impairments, activity limitations and participation restrictions in children with juvenile idiopathic arthritis. *Clin Exp Rheumatol* 2010; 28: 905-911.

21.	Eboli C, Conti EA, Chironi F, et al. Understanding the role of ultrasound in detecting temporomandibular joint involvement in juvenile idiopathic arthritis: A pilot study. *Pediatr Rheumatol* 2022; 20. DOI: 10.1186/s12969-022-00729-z.

22.	Elfving A, Harila-Saari A, Nilsson L, et al. An explorative study on proteomic analyses related to inflammation and pain in children with juvenile idiopathic arthritis. *BMC Pediatr* 2023; 23: 365. DOI: 10.1186/s12887-023-04181-0.

23.	Elliott MJ, Woo P, Charles P, et al. Suppression of fever and the acute-phase response in a patient with juvenile chronic arthritis treated with monoclonal antibody to tumour necrosis factor-α (cA2). *Br J Rheumatol* 1997; 36: 589-593.

24.	Esbersen S, Herlin T and Mahler BT. Daily physical activity and sleep in children with juvenile idiopathic arthritis and healthy children in relation to pain. *Pediatr Rheumatol* 2018; 16. DOI: 10.1186/s12969-018-0265-6.

25.	Flatø B, Smerdel A, Johnston V, et al. The influence of patient characteristics, disease variables, and HLA alleles on the development of radiographically evident sacroiliitis in juvenile idiopathic arthritis. *Arthritis Rheum* 2002; 46: 986-994. DOI: 10.1002/art.10146.

26.	Ferrazzo VA, Guerino P, Assaf DC, et al. Juvenile idiopathic arthritis with severe unilateral temporomandibular joint involvement. *J Oral Pathol Med* 2019; 48: 66. DOI: https://onlinelibrary.wiley.com/doi/10.1111/jop.12796.

27.	Gestler G, Rocha AM, Len CA, et al. Residual complaints in juvenile idiopathic arthritis patients with inactive disease. *Pediatr Rheumatol* 2022; 20. DOI: 10.1186/s12969-022-00729-z.

28.	Gorczyca D, Postepski J, Czajkowska A, et al. Serum levels of myeloid-related proteins 8 and 14 in oligoarticular and polyarticular onset of Juvenile Idiopathic Arthritis. *Pediatr Rheumatol* 2017; 15. DOI: 10.1186/s12969-017-0141-9.

29.	Guzman J, Henrey A, Loughin T, et al. Predicting which children with juvenile idiopathic arthritis will have a severe disease course: Results from the ReACCh-Out cohort. *J Rheumotol* 2017; 44: 230-240. DOI: 10.3899/jrheum.160197.

30.	Hara T, Kishi T, Tani Y, et al. Usefulness of joint ultrasonography in the treatment of juvenile idiopathic arthritis. *Pediatr Rheumatol* 2017; 15.

31.	Hogewe JA, Kuis W, Huygen ACJ, et al. The pain threshold in juvenile chronic arthritis. *Rheumatology (Oxford)* 1995; 34: 61-67. DOI: 10.1093/rheumatology/34.1.61.

32.	Hospach T, Maier J, Müller-Abt P, et al. Cervical spine involvement in patients with juvenile idiopathic arthritis - MRI follow-up study. *Pediatr Rheumatol* 2014; 12: 9. DOI: 10.1186/1546-0096-12-9.

33.	Hu X, Xie Q, Mo X, et al. The role of extracellular histones in systemic-onset juvenile idiopathic arthritis. *Ital J Pediatr* 2019; 45: 14. DOI: 10.1186/s13052-019-0605-2.

34.	Ilowite NT, Walco GA and Pochaczevsky R. Assessment of pain in patients with juvenile rheumatoid arthritis: relation between pain intensity and degree of joint inflammation. *Ann Rheum Dis* 1992; 51: 343-346. DOI: 10.1136/ard.51.3.343.

35.	Katsicas M, Carrara C, Bernasconi A, et al. Enthesitis-related arthritis: Non-peripheral pattern is associated with th17 cells. *Ann Rheum Dis* 2017; 76: 403. DOI: 10.1136/annrheumdis-2017-eular.6726.

36.	Katsicas M, Galeano M, Pradier M, et al. Spondyloarthritis research consortium of Canada sacroiliac joint inflammation score in children with enthesitis-related arthritis. *Pediatr Rheumatol* 2018; 16. DOI: <https://dx.doi.org/10.1186/s12969-018-0265-6>.

37.	Khanom S. *Pain Flares in Adolescents with Inflammatory and Non-Inflammatory Musculoskeletal Disorders*. ProQuest Dissertations & Theses, 2022.

38.	Kovalchuk T, Pavlyshyn H, Boyarchuk O, et al. The difference in pain and overall well-being assessment between patients with juvenile idiopathic arthritis, their parents, and physicians in Ukraine. *Pediatr Pol* 2018; 93: 298-305. DOI: 10.5114/polp.2018.77994.

39.	Küseler A, Pedersen TK, Gelineck J, et al. A 2 year followup study of enhanced magnetic resonance imaging and clinical examination of the temporomandibular joint in children with juvenile idiopathic arthritis. *J Rheumatol* 2005; 32: 162-169.

40.	Learoyd AE, Sen D and Fitzgerald M. The pain trajectory of juvenile idiopathic arthritis (JIA): Translating from adolescent patient report to behavioural sensitivity in a juvenile animal model. *Pediatr Rheumatol* 2019; 17: 60. DOI: 10.1186/s12969-019-0360-3.

41.	Leksell E, Ernberg M, Magnusson B, et al. Orofacial pain and dysfunction in children with juvenile idiopathic arthritis: A case-control study. *Scand J Rheumatol* 2012; 41: 375-378. DOI: 10.3109/03009742.2012.675585.

42.	Lomholt JJ, Thastum M and Herlin T. Pain experience in children with juvenile idiopathic arthritis treated with anti-TNF agents compared to non-biologic standard treatment. *Pediatr Rheumatol* 2013; 11. DOI: 10.1186/1546-0096-11-21.

43.	Magni-Manzoni S, Garay S, Cugno C, et al. Responsiveness of clinical measures to flare of disease activity in juvenile idiopathic arthritis. *Clin Exp Rheumatol* 2005; 23: 421-425.

44.	Magni-Manzoni S, Epis O, Ravelli A, et al. Comparison of clinical versus ultrasound-determined synovitis in juvenile idiopathic arthritis. *Arthritis Care Res* 2009; 61: 1497-1504. DOI: 10.1002/art.24823.

45.	Mahmood K, Khan A and Clinch J. Cervical spine involvement in JIA: A clinical and radiological correlation. *Rheumatology (Oxford)* 2014; 53: i165-i166. DOI: <https://dx.doi.org/10.1093/rheumatology/keu125.007>.

46.	Malattia C, Damasio MB, Basso C, et al. Dynamic contrast-enhanced magnetic resonance imaging in the assessment of disease activity in patients with juvenile idiopathic arthritis. *Rheumatology (Oxford)* 2010; 49: 178-185. DOI: 10.1093/rheumatology/kep343.

47.	Malleson PN, Oen K, Cabral DA, et al. Predictors of Pain in Children with Established Juvenile Rheumatoid Arthritis. *Arthritis Care Res* 2004; 51: 222-227. DOI: 10.1002/art.20238.

48.	Mannion M, Xie F and Beukelman T. Investigation of inactive disease activity states among JIA patients in the Carra registry. *Arthritis Rheumatol* 2019; 71: 4815-4817. DOI: <https://dx.doi.org/10.1002/art.41108>.

49.	Mannion M, Amin S, Balevic S, et al. Outcomes of patients with juvenile idiopathic arthritis following biologic switching in the childhood arthritis and rheumatology research alliance registry. *Pediatr Rheumatol* 2022; 20. DOI: <https://dx.doi.org/10.1186/s12969-022-00729-z>.

50.	McGrath T, Guzman J, Tucker L, et al. Predictors of persisting pain in children with Juvenile Idiopathic Arthritis: a case control study nested in the ReACCh-Out cohort. *Pediatr Rheumatol* 2023; 21: 1-7. DOI: 10.1186/s12969-023-00885-w.

51.	McLellan K, Saeed N and Compeyrot-Lacassagne S. Management of Temporomandibular Joint Involvement(TMJ) in Juvenile Idiopathic Arthritis (JIA) in our Centre. *Pediatr Rheumatol* 2023; 21. DOI: <https://dx.doi.org/10.1186/s12969-023-00879-8>.

52.	Melchiorre D, Falcini F, Kaloudi O, et al. Sonographic evaluation of the temporomandibular joints in juvenile idiopathic arthritis. *J Ultrasound* 2010; 13: 34-37. DOI: 10.1016/j.jus.2009.09.008.

53.	Miller ML, Ruprecht J, Wang D, et al. Physician assessment of disease activity in JIA subtypes. Analysis of data extracted from electronic medical records. *Pediatr Rheumatol Online J* 2011; 9: 9. DOI: <https://dx.doi.org/10.1186/1546-0096-9-9>.

54.	Min M, Crotti T and Boros C. Establishment of a registry for juvenile idiopathic arthritis patients in South Australia: Focus on patient-reported outcome and experience measures (PROMS/PREMS). *Pediatr Rheumatol* 2020; 18. DOI: <https://dx.doi.org/10.1186/s12969-020-00470-5>.

55.	Minden K, Klotsche J, Niewerth M, et al. The health status of patients with juvenile idiopathic arthritis (JIA) significantly worsens after transfer from pediatric to adult care. *Arthritis Rheumatol* 2014; 66: S1268-S1269. DOI: <https://dx.doi.org/10.1002/art.38914>.

56.	Miyamae T, Tani Y and Yamanaka H. Juvenile idiopathic arthritis with no complaints of joint pain in early childhood. *Mod Rheumatol Case Rep* 2019; 3: 10-13. DOI: 10.1080/24725625.2018.1507272.

57.	Mohammed Y, Saeed O, Zaghloul N, et al. Juvenile idiopathic arthritis and the temporomandibular joint. *Alex J Med* 2012; 48: 123-129. DOI: <https://dx.doi.org/10.1016/j.ajme.2011.11.004>.

58.	Mosa DM. Bicipital synovial cyst as the sole musculoskeletal presentation of systemic juvenile idiopathic arthritis—case report. *Mod Rheumatol Case Rep* 2021; 5: 246-249. DOI: 10.1080/24725625.2020.1869509.

59.	Mountain DC, Shoop-Worrall S, Cordingley L, et al. The interplay between pain and disease activity: personal models of pain beliefs and emotional representations in children and young people with juvenile idiopathic arthritis in a UK nationwide prospective inception cohort. *J Pediatr Psychol* 2025. DOI: <https://dx.doi.org/10.1093/jpepsy/jsaf024>.

60.	Naddei R, Burrone M, Ridella F, et al. Determinants of parent/patient overall well-being poor ratings in juvenile idiopathic arthritis patients with inactive disease. *Pediatr Rheumatol* 2023; 21. DOI: <https://dx.doi.org/10.1186/s12969-023-00879-8>.

61.	Nguyen K, Barsalou J, Basodan D, et al. A decade of progress in juvenile idiopathic arthritis treatments and outcomes in Canada: results from ReACCh-Out and the CAPRI registry. *Rheumatology (Oxford)* 2024; 63: SI173-SI179. DOI: 10.1093/rheumatology/kead560.

62.	Nistala K, Babar J, Johnson K, et al. Clinical assessment and core outcome variables are poor predictors of hip arthritis diagnosed by MRI in juvenile idiopathic arthritis. *Rheumatology (Oxford)* 2007; 46: 699-702. DOI: <https://dx.doi.org/10.1093/rheumatology/kel401>.

63.	Oen K, Tucker L, Huber AM, et al. Predictors of early inactive disease in a juvenile idiopathic arthritis cohort: Results of a Canadian Multicenter, prospective inception cohort study. *Arthritis Care Res* 2009; 61: 1077-1086. DOI: 10.1002/art.24539.

64.	Oen K, Guzman J, Dufault B, et al. Health-Related Quality of Life in an Inception Cohort of Children With Juvenile Idiopathic Arthritis: A Longitudinal Analysis. *Arthritis Care Res* 2018; 70: 134-144. DOI: 10.1002/acr.23236.

65.	Oen K, Tian J, Loughin TM, et al. Causal pathways to health-related quality of life in children with juvenile idiopathic arthritis: Results from the ReACCh-Out cohort. *Rheumatology (Oxford)* 2021; 60: 4691-4702. DOI: 10.1093/rheumatology/keab079.

66.	Palmisani E, Solari N, Magni-Manzoni S, et al. Correlation between juvenile idiopathic arthritis activity and damage measures in early, advanced, and longstanding disease. *Arthritis Care Res* 2006; 55: 843-849. DOI: 10.1002/art.22357.

67.	Papsdorf V and Horneff G. Complete control of disease activity and remission induced by treatment with etanercept in juvenile idiopathic arthritis. *Rheumatology (Oxford)* 2011; 50: 214-221. DOI: 10.1093/rheumatology/keq292.

68.	Pedersen TK, Küseler A, Gelineck J, et al. A prospective study of magnetic resonance and radiographic imaging in relation to symptoms and clinical findings of the temporomandibular joint in children with juvenile idiopathic arthritis. *J Rheumatol* 2008; 35: 1668-1675.

69.	Pinto JRR, Pedron IG, Utumi ER, et al. Temporomandibular joint disorders as the only manifestation of juvenile idiopathic arthritis: a case report. *Einstein (Sao Paulo)* 2018; 16: eRC4003. DOI: 10.1590/S1679-45082018RC4003.

70.	Portelli M, Matarese G, Militi A, et al. Temporomandibular joint involvement in a cohort of patients with Juvenile Idiopatic Arthritis and evaluation of the effect induced by functional orthodontic appliance: clinical and radiographic investigation. *Eur J Paediatr Dent* 2014; 15: 63-66.

71.	Quilis N, Mesa-del-Castillo Bermejo P, Boix P, et al. Peripheral blood regulatory T cells and disease activity, quality of life, and outcomes in children with juvenile idiopathic arthritis. *Pediatr Rheumatol* 2024; 22: Article 69. DOI: 10.1186/s12969-024-01006-x.

72.	Radziszewska A, Fisher C, Bray T, et al. Cytokine signature does not correlate with pain or disease activity in well controlled ERA. *Ann Rheum Dis* 2019; 78: 1571-1572. DOI: <https://dx.doi.org/10.1136/annrheumdis-2019-eular.2827>.

73.	Randell RL, Reeve BB, Weitzman ER, et al. Pain Interference in Juvenile Idiopathic Arthritis. *J Rheumatol* 2024; 51: 1119-1124. DOI: 10.3899/jrheum.2024-0254.

74.	Rapoff MA, Lindsley CB and Purviance MR. The validity and reliability of parental ratings of disease activity in juvenile rheumatoid arthritis. *Arthritis Rheum* 1991; 4: 136-139. DOI: 10.1002/art.1790040306.

75.	Rashid A, Cordingley L, Carrasco R, et al. Patterns of pain over time among children with Juvenile Idiopathic Arthritis. *Arch Dis Child* 2018; 103: 437-443.

76.	Ravelli A, Viola S, Ruperto N, et al. Correlation between conventional disease activity measures in juvenile chronic arthritis. *Ann Rheum Dis* 1997; 56: 197-200. DOI: 10.1136/ard.56.3.197.

77.	Ross CK, Lavigne JV, Hayford JR, et al. Validity of reported pain as a measure of clinical state in juvenile rheumatoid arthritis. *Ann Rheum Dis* 1989; 48: 817-819. DOI: 10.1136/ard.48.10.817.

78.	Rumsey DG, Guzman J, Rosenberg AM, et al. Worse Quality of Life, Function, and Pain in Children With Enthesitis, Irrespective of Their Juvenile Arthritis Category. *Arthritis Care Res* 2020; 72: 441-446. DOI: 10.1002/acr.23844.

79.	Rypdal V, Glerup M, Rypdal M, et al. Disease activity trajectories from childhood to adulthood in the population-based Nordic juvenile idiopathic arthritis cohort. *RMD Open* 2024; 10. DOI: 10.1136/rmdopen-2023-003759.

80.	Schanberg LE, Lefebvre JC, Keefe FJ, et al. Pain coping and the pain experience in children with juvenile chronic arthritis. *PAIN* 1997; 73: 181-189. DOI: 10.1016/S0304-3959(97)00110-3.

81.	Schanberg LE, Anthony KK, Gil KM, et al. Daily pain and symptoms in children with polyarticular arthritis. *Arthritis Rheum* 2003; 48: 1390-1397. DOI: 10.1002/art.10986.

82.	Scott PJ, Ansell BM and Huskisson EC. Measurement of pain in juvenile chronic polyarthritis. *Ann Rheum Dis* 1977; 36: 186-187. DOI: 10.1136/ard.36.2.186.

83.	Sgarlat CM, Pelajo CF, Lopez-Benitez JM, et al. Physician versus parent/patient assessment of disease activity in a large cohort of patients with juvenile idiopathic arthritis. *Arthritis Rheum* 2011; 63.

84.	Sherry DD, Bohnsack J, Salmonson K, et al. Painless juvenile rheumatoid arthritis. *J Pediatr* 1990; 116: 921-923. DOI: 10.1016/S0022-3476(05)80652-3.

85.	Shiff NJ, Tupper S, Oen K, et al. Trajectories of pain severity in juvenile idiopathic arthritis: Results from the Research in Arthritis in Canadian Children Emphasizing Outcomes cohort. *PAIN* 2018; 159: 57-66. DOI: 10.1097/j.pain.0000000000001064.

86.	Shoop-Worrall S, Verstappen S, McDonagh J, et al. Predicting remission from one year following initial presentation in a multicentre inception cohort of patients with juvenile idiopathic arthritis. *Rheumatology (Oxford)* 2018; 57: iii37. DOI: <https://dx.doi.org/10.1093/rheumatology/key075.211>.

87.	Shoop-Worrall SJW, Hyrich KL, Wedderburn LR, et al. Patient-reported wellbeing and clinical disease measures over time captured by multivariate trajectories of disease activity in individuals with juvenile idiopathic arthritis in the UK: a multicentre prospective longitudinal study. *Lancet Rheumatol* 2021; 3: e111-e121. DOI: 10.1016/S2665-9913(20)30269-1.

88.	Spadaro A, Riccieri V, Scavalli AS, et al. Interleukin-6 and soluble interleukin-2 receptor in juvenile chronic arthritis: Correlations with clinical and laboratory parameters. *Rev Rhum Engl Ed* 1996; 63: 171-177.

89.	Spekking K, Anink J, de Boer P, et al. Significant pain decrease in children with non-systemic Juvenile Idiopathic Arthritis treated to target: results over 24 months of follow up. *Pediatr Rheumatol* 2023; 21: 1-9. DOI: 10.1186/s12969-023-00874-z.

90.	Stoll ML, Bhore R, Dempsey-Robertson M, et al. Spondyloarthritis in a Pediatric Population: Risk Factors for Sacroiliitis. *J Rheumatol* 2010; 37: 2402-2408. DOI: 10.3899/jrheum.100014.

91.	Stoustrup P, Twilt M, Koos B, et al. Temporomandibular pain in patients with Juvenile Idiopathic Arthritis. *J Rheumatol* 2016; 43: 1209-1210. DOI: <https://dx.doi.org/10.3899/jrheum.160272>.

92.	Sudhakar M, Deswal S, Sachdev N, et al. The reliability of Juvenile Arthritis Magnetic Resonance Imaging Scoring system in the evaluation of the shoulder joint in juvenile idiopathic arthritis. *Arch Rheumatol* 2022; 37: 547-558. DOI: 10.46497/ArchRheumatol.2022.9304.

93.	Svensson B, Adell R and Kopp S. Temporomandibular disorders in Juvenile Chronic Arthritis patients. A clinical study. *Swed Dent J* 2000; 24: 83-92.

94.	Svensson B, Larsson Å and Adell R. The mandibular condyle in juvenile chronic arthritis patients with mandibular hypoplasia: A clinical and histological study. *Int J Oral and Maxillofac Surg* 2001; 30: 300-305. DOI: 10.1054/ijom.2001.0058.

95.	Tarakci E, Yeldan I, Kaya Mutlu E, et al. The relationship between physical activity level, anxiety, depression, and functional ability in children and adolescents with juvenile idiopathic arthritis. *Clin Rheumatol* 2011; 30: 1415-1420. DOI: 10.1007/s10067-011-1832-0.

96.	Tarkiainen M, Tynjala P, Vahasalo P, et al. Early aggressive treatment decreases pain in patients with juvenile idiopathic arthritis. *Pediatr Rheumatol* 2018; 16. DOI: <https://dx.doi.org/10.1186/s12969-018-0265-6>.

97.	Thastum M and Herlin T. Pain-specific beliefs and pain experience in children with juvenile idiopathic arthritis: A longitudinal study. *J Rheumatol* 2011; 38: 155-160. DOI: 10.3899/jrheum.091375.

98.	Thompson KL, Varni JW and Hanson V. Comprehensive Assessment of Pain in Juvenile Rheumatoid Arthritis: An Empirical Model. *J Pediatr Psychol* 1987; 12: 241-255. DOI: 10.1093/jpepsy/12.2.241.

99.	Tirelli F, Xiao R, Brandon TG, et al. Determinants of disease activity change over time in Enthesitis related arthritis: effect of structured outcome monitoring and clinical decision support. *Pediatr Rheumatol* 2020; 18. DOI: 10.1186/s12969-020-00472-3.

100.	Trachtman R, Wang CM, Murray E, et al. PROMIS Computer Adaptive Tests and Their Correlation with Disease Activity in Juvenile Idiopathic Arthritis. *J Clin Rheumatol* 2021; 27: 131-135. DOI: 10.1097/RHU.0000000000001171.

101.	Trachtman R, Issa R, Pan S, et al. The value of the patient global health assessment in polyarticular juvenile idiopathic arthritis: a nested cohort study. *J Patient Rep Outcomes* 2021; 5: 50. DOI: <https://dx.doi.org/10.1186/s41687-021-00328-8>.

102.	Tripalo Batoš A, Potočki K, Žutelija Fattorini M, et al. Is axial magnetic resonance imaging useful in early juvenile spondyloarthritis—preliminary report. *Clin Rheumatol* 2020; 39: 3017-3025. DOI: 10.1007/s10067-020-05037-z.

103.	Tucker LB, Oen K, Yeung RSM, et al. Correlates of pain in an inception cohort of children with juvenile idiopathic arthritis (JIA): Research in arthritis in Canadian children. *J Rheumatol* 2009; 36: 2583. DOI: <https://dx.doi.org/10.3899/jrheum.090763>.

104.	Tupper SM, Rosenberg AM, Pahwa P, et al. Pain intensity variability and its relationship with quality of life in youths with juvenile idiopathic arthritis. *Arthritis Care Res* 2013; 65: 563-570. DOI: 10.1002/acr.21850.

105.	Ünal E, Batu Akal ED, Sonmez EH, et al. Biopsychosocial status of jia patients: Perspectives of daily living activities, disease activity and family impact. *Ann Rheum Dis* 2018; 77: 1855

EP - 1856. DOI: <https://dx.doi.org/10.1136/annrheumdis-2018-eular.7248>.

106.	Upadhyay J, Lemme J, Cay M, et al. A multidisciplinary assessment of pain in juvenile idiopathic arthritis. *Semin Arthritis Rheum* 2021; 51: 700-711. DOI: 10.1016/j.semarthrit.2021.05.011.

107.	Van Der Heijden H, Fatou B, Sibai D, et al. Proteomics based markers of clinical pain severity in juvenile idiopathic arthritis. *Pediatr Rheumatol Online J* 2022; 20: 3. DOI: <https://dx.doi.org/10.1186/s12969-022-00662-1>.

108.	van Dijkhuizen EHP, Ridella F, Naddei R, et al. Validity and Reliability of Four Parent/Patient–Reported Outcome Measures for Juvenile Idiopathic Arthritis Remote Monitoring. *Arthritis Care Res* 2023; 75: 391-400. DOI: 10.1002/acr.24855.

109.	Vandvik IH and Eckblad G. Relationship between pain, disease severity and psychosocial function in patients with juvenile chronic arthritis (JCA). *Scand J Rheumatol* 1990; 19: 295-302. DOI: <https://dx.doi.org/10.3109/03009749009102536>.

110.	Varni JW, Thompson KL and Hanson V. The Varni/Thompson Pediatric Pain Questionnaire. I: Chronic musculoskeletal pain in juvenile rheumatoid arthritis. *PAIN* 1987; 28: 27-38. DOI: 10.1016/0304-3959(87)91056-6.

111.	Vega-Fernandez P, Oberle EJ, Henrickson M, et al. Musculoskeletal Ultrasound and the Assessment of Disease Activity in Juvenile Idiopathic Arthritis. *Arthritis Care Res* 2023; 75: 1815-1820. DOI: <https://dx.doi.org/10.1002/acr.25073>.

112.	Verstappen SMM, Cobb J, Foster HE, et al. The Association Between Low Socioeconomic Status With High Physical Limitations and Low Illness Self‐Perception in Patients With Juvenile Idiopathic Arthritis: Results From the Childhood Arthritis Prospective Study. *Arthritis Care Res* 2015; 67: 382-389. DOI: 10.1002/acr.22466.

113.	Viswanath V, Myles A, Dayal R, et al. Levels of serum matrix metalloproteinase-3 correlate with disease activity in the enthesitis-related arthritis category of juvenile idiopathic arthritis. *J Rheumatol* 2011; 38: 2482-2487. DOI: 10.3899/jrheum.110352.

114.	von Schuckmann L, Klotsche J, Suling A, et al. Temporomandibular joint involvement in patients with juvenile idiopathic arthritis: a retrospective chart review. *Scand J Rheumatol* 2020; 49: 271-280. DOI: 10.1080/03009742.2020.1720282.

115.	Vuorimaa H, Tamm K, Honkanen V, et al. Empirical classification of children with JIA: A multidimensional approach to pain and well-being. *Clin Exp Rheumatol* 2008; 26: 954-961.

116.	Weiss PF, Beukelman T, Schanberg LE, et al. Enthesitis-related Arthritis Is Associated with Higher Pain Intensity and Poorer Health Status in Comparison with Other Categories of Juvenile Idiopathic Arthritis: The Childhood Arthritis and Rheumatology Research Alliance Registry. *J Rheumatol* 2012; 39: 2341-2351. DOI: 10.3899/jrheum.120642.

117.	Weiss PF, Xiao R, Biko DM, et al. Assessment of Sacroiliitis at Diagnosis of Juvenile Spondyloarthritis by Radiography, Magnetic Resonance Imaging, and Clinical Examination. *Arthritis Care Res* 2016; 68: 187-194. DOI: 10.1002/acr.22665.

118.	Xu LY, Zhang WM, Xia M, et al. Potential Effects of Interleukins on the Pathogenesis of Systemic Onset Juvenile Idiopathic Arthritis. *Pediatr Neonatol* 2016; 57: 396-401. DOI: 10.1016/j.pedneo.2015.09.010.

119.	Yamazaki K, Fujikawa A, Nozaki T, et al. MRI evaluation of active sacroiliitis in juvenile spondyloarthritis treated with TNF inhibitors. *Pediatr Rheumatol* 2022; 20. DOI: <https://dx.doi.org/10.1186/s12969-022-00729-z>.

120.	Yilmaz MH, Ozbayrak M, Kasapcopur O, et al. Pelvic MRI findings of juvenile-onset ankylosing spondylitis. *Clin Biomech* 2010; 29: 1007-1013. DOI: 10.1007/s10067-010-1514-3.

121.	Zhang H, Wei X, Liu W, et al. Latent class analysis identifies distinct pain phenotypes in newly diagnosed systemic juvenile idiopathic arthritis. *Arthritis Res Ther* 2025; 27: 71. DOI: 10.1186/s13075-025-03534-7.

122.	Zwir L, Terreri MT, Sousa S et al. Does intense synovial enhancement in temporomandibular joints of juvenile idiopathic arthritis patients correlate with disease activity? Ann Rheum Dis 2012;71(Suppl 3):264–5. doi: 10.1136/annrheumdis-2012-eular.2287.
